# Supplementary figures and images for: The BLM-TOP3A-RMI1-RMI2 proximity map reveals that RAD54L2 suppresses sister chromatid exchanges
Source: EMBO Rep. 2025 Jan 27;26(5):1290–314. doi: 10.1038/s44319-025-00374-z (PMC11894219; doi:10.1038/s44319-025-00374-z)

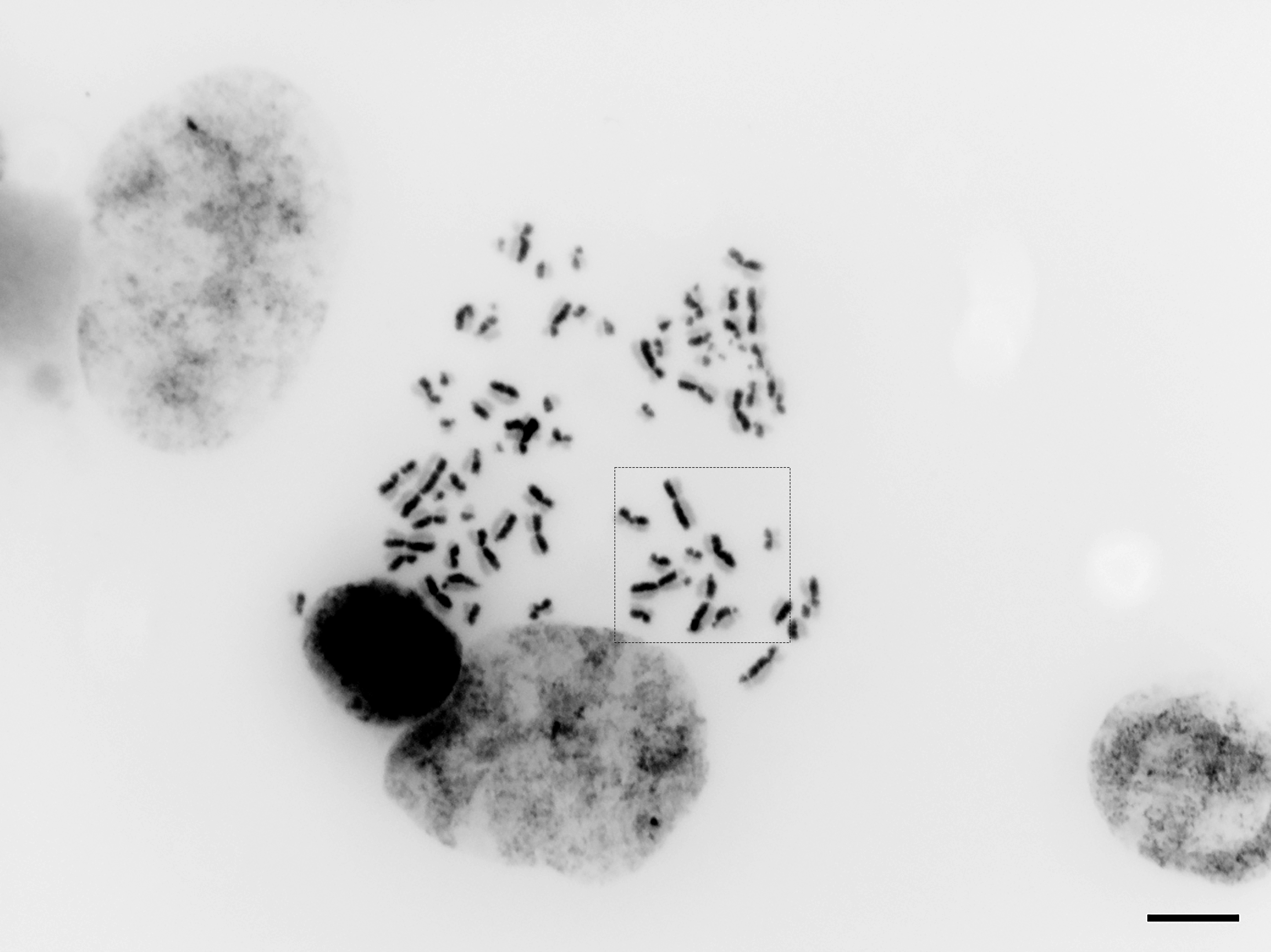

Supplement: Supplementary file 4 — Source data Fig. 3 [file 44319_2025_374_MOESM4_ESM.zip › Figure 3/SourceData3A/SourceDataForFigure3A.siCTRL.adj.tif]

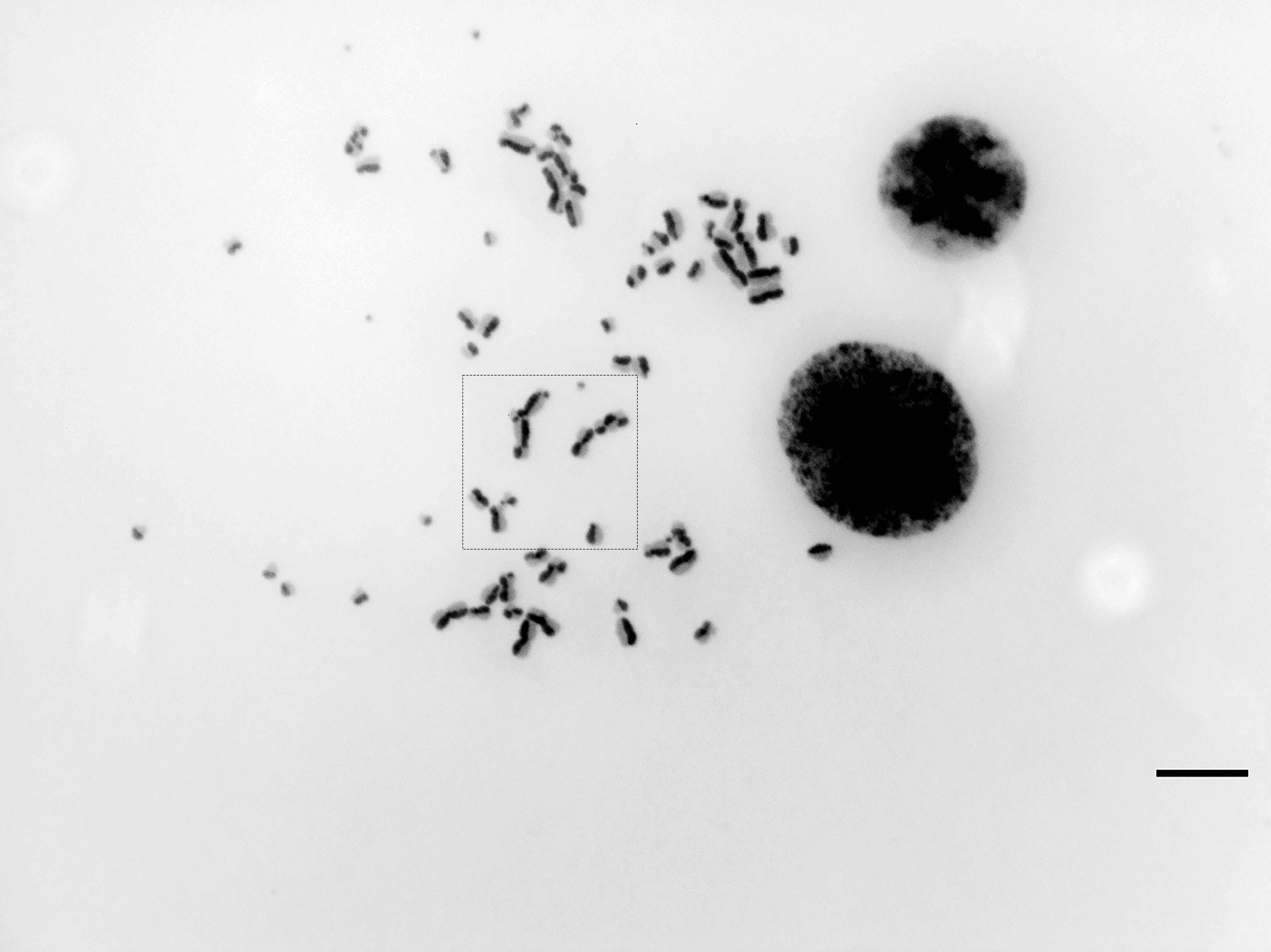

Supplement: Supplementary file 4 — Source data Fig. 3 [file 44319_2025_374_MOESM4_ESM.zip › Figure 3/SourceData3A/SourceDataForFigure3A.siBLM.adj.tif]

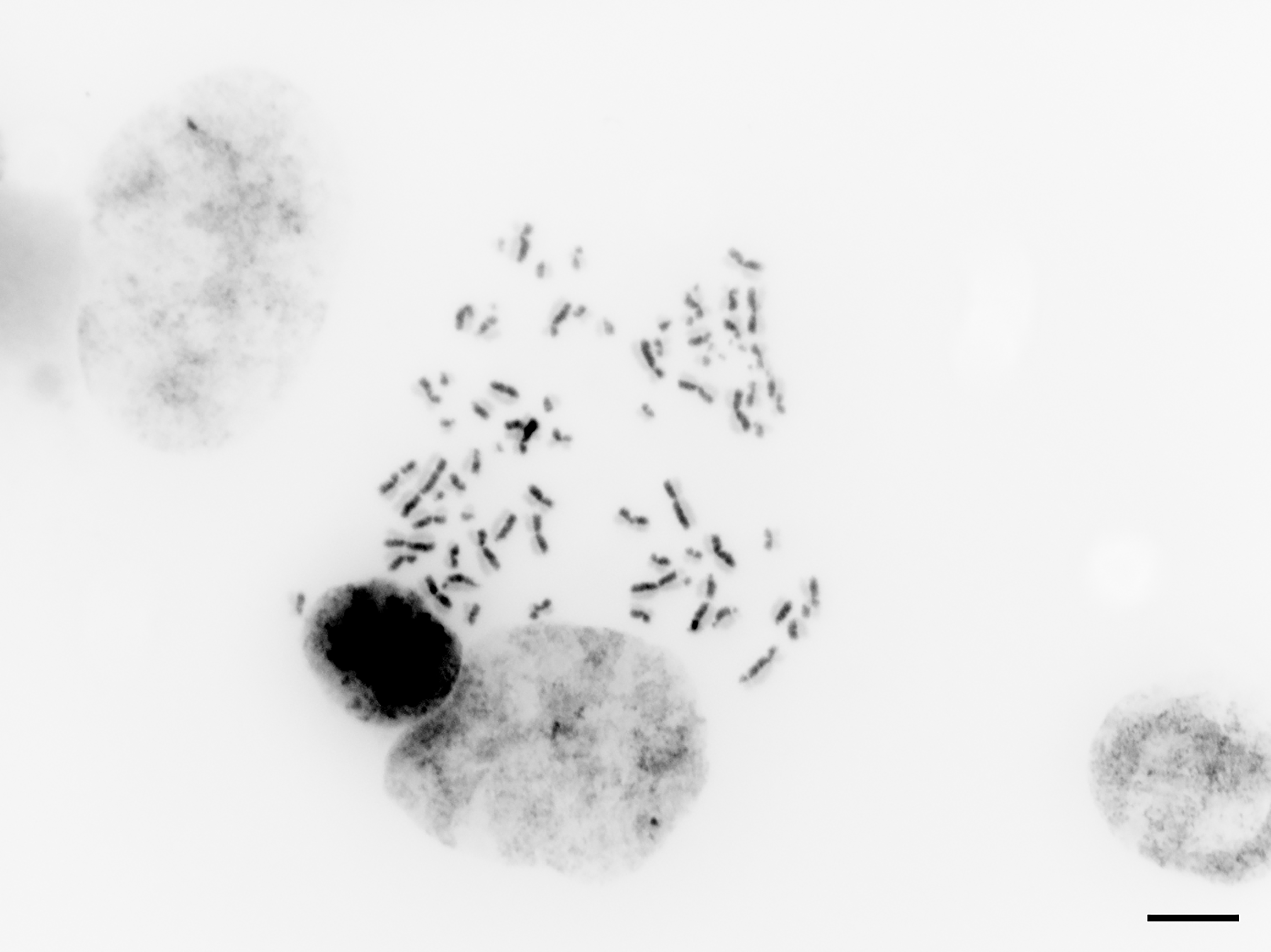

Supplement: Supplementary file 4 — Source data Fig. 3 [file 44319_2025_374_MOESM4_ESM.zip › Figure 3/SourceData3A/SourceDataForFigure3A.siCTRL.raw.tif]

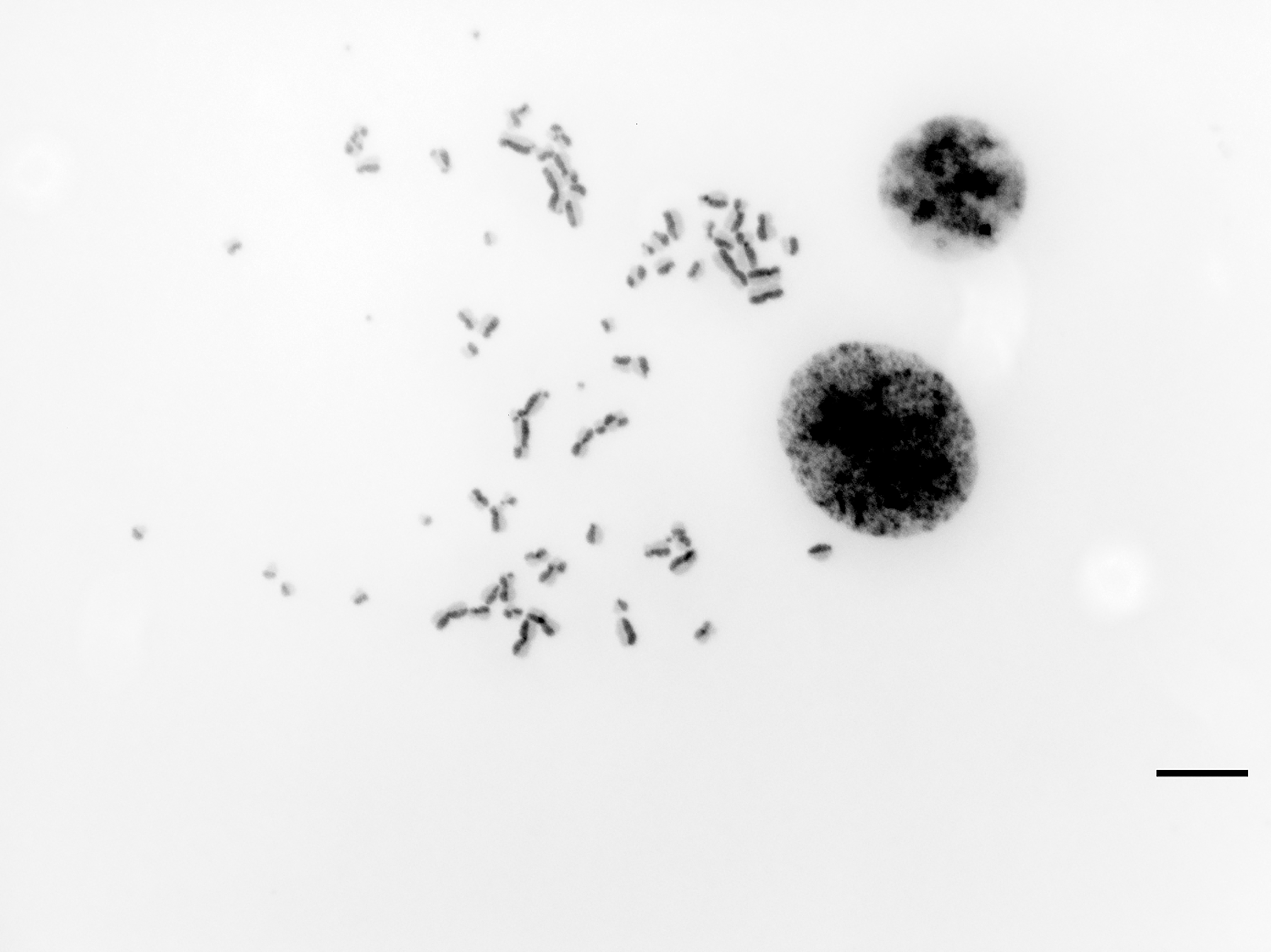

Supplement: Supplementary file 4 — Source data Fig. 3 [file 44319_2025_374_MOESM4_ESM.zip › Figure 3/SourceData3A/SourceDataForFigure3A.siBLM.raw.tif]

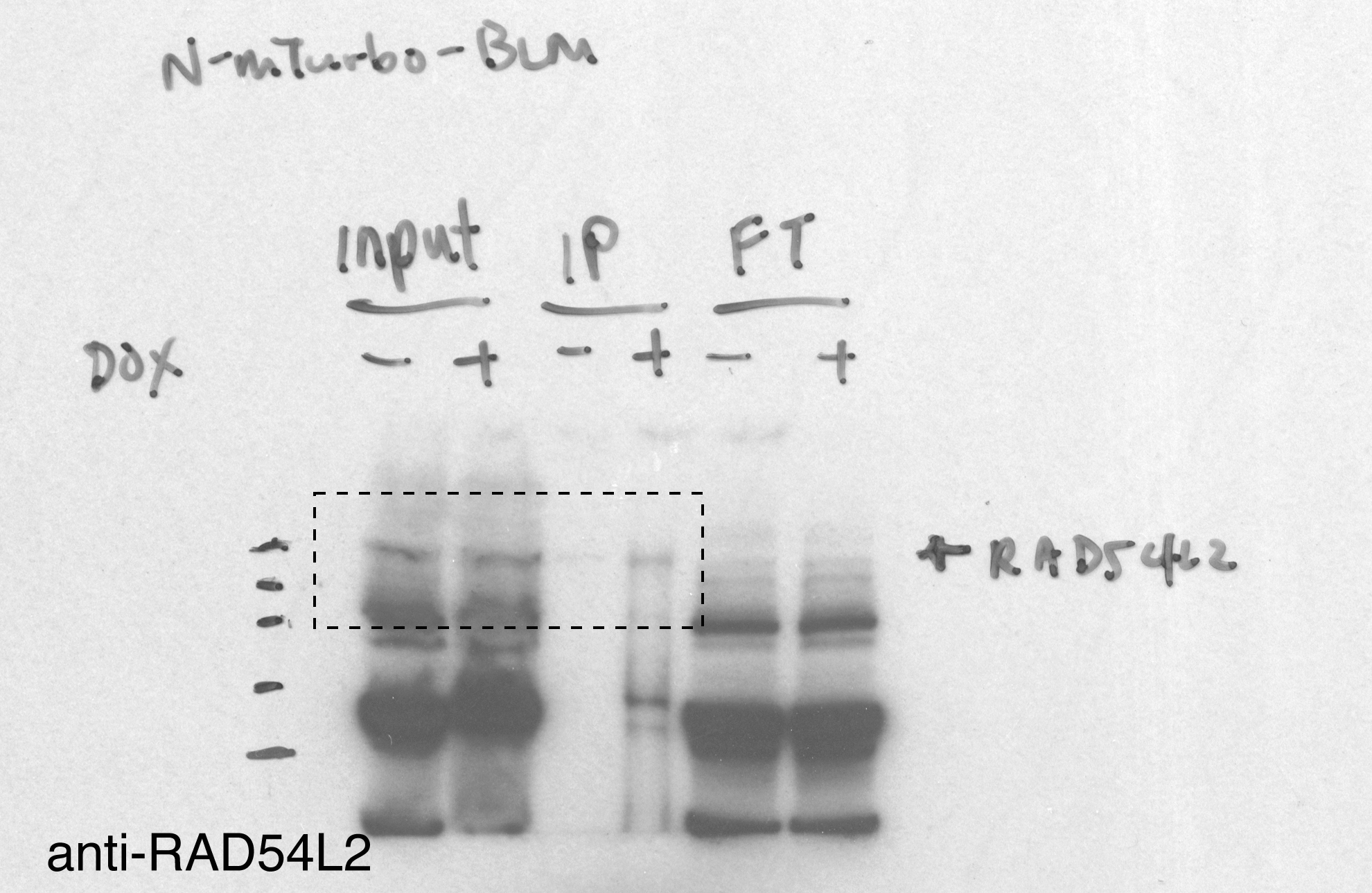

Supplement: Supplementary file 5 — Source data Fig. 4 [file 44319_2025_374_MOESM5_ESM.zip › Figure 4/SourceData4C/SourceDataForFigure4C.RAD54L2.tif]

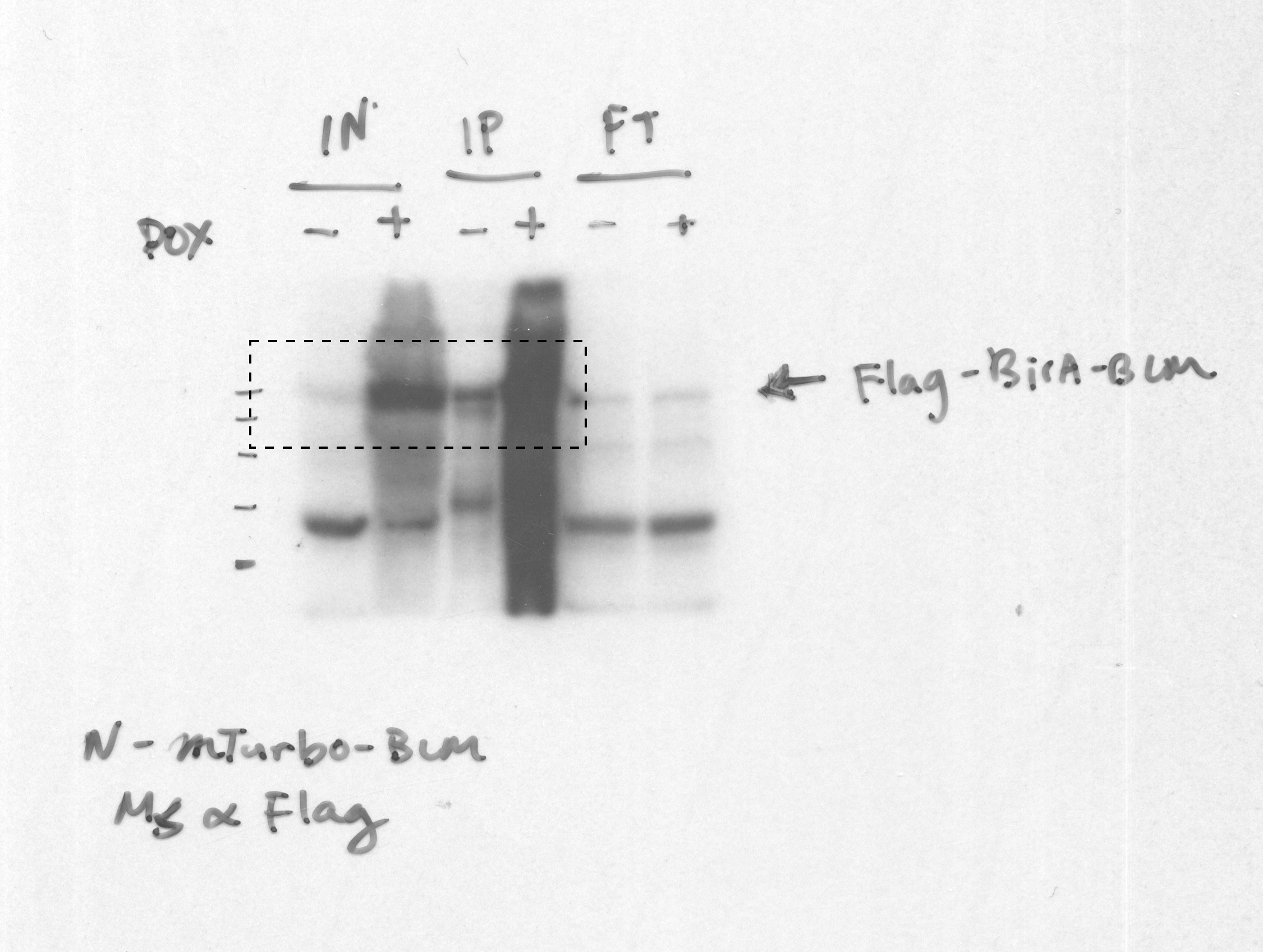

Supplement: Supplementary file 5 — Source data Fig. 4 [file 44319_2025_374_MOESM5_ESM.zip › Figure 4/SourceData4C/SourceDataForFigure4C.BLM.tif]

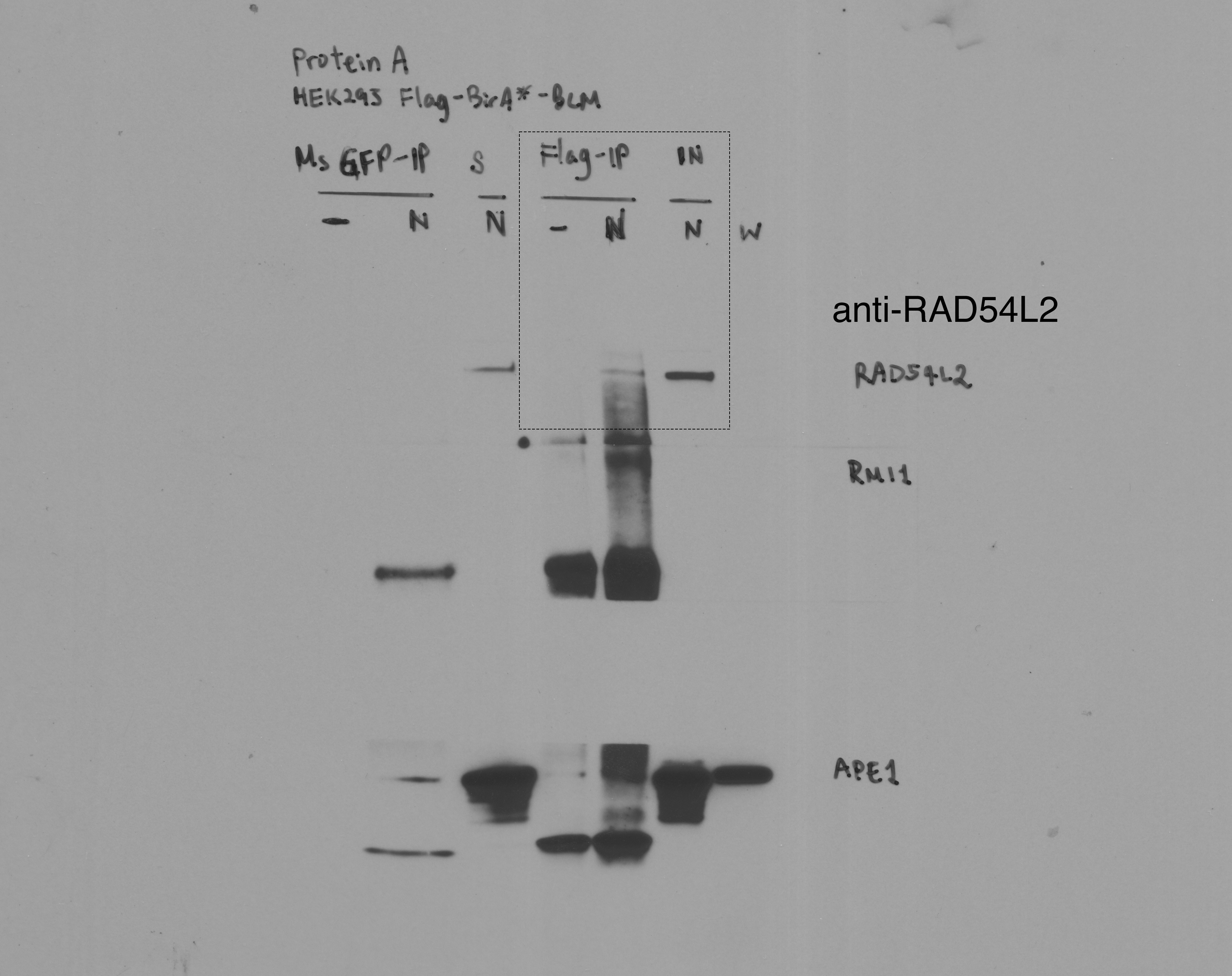

Supplement: Supplementary file 5 — Source data Fig. 4 [file 44319_2025_374_MOESM5_ESM.zip › Figure 4/SourceData4B/SourceDataForFigure4B.RAD54L2.tif]

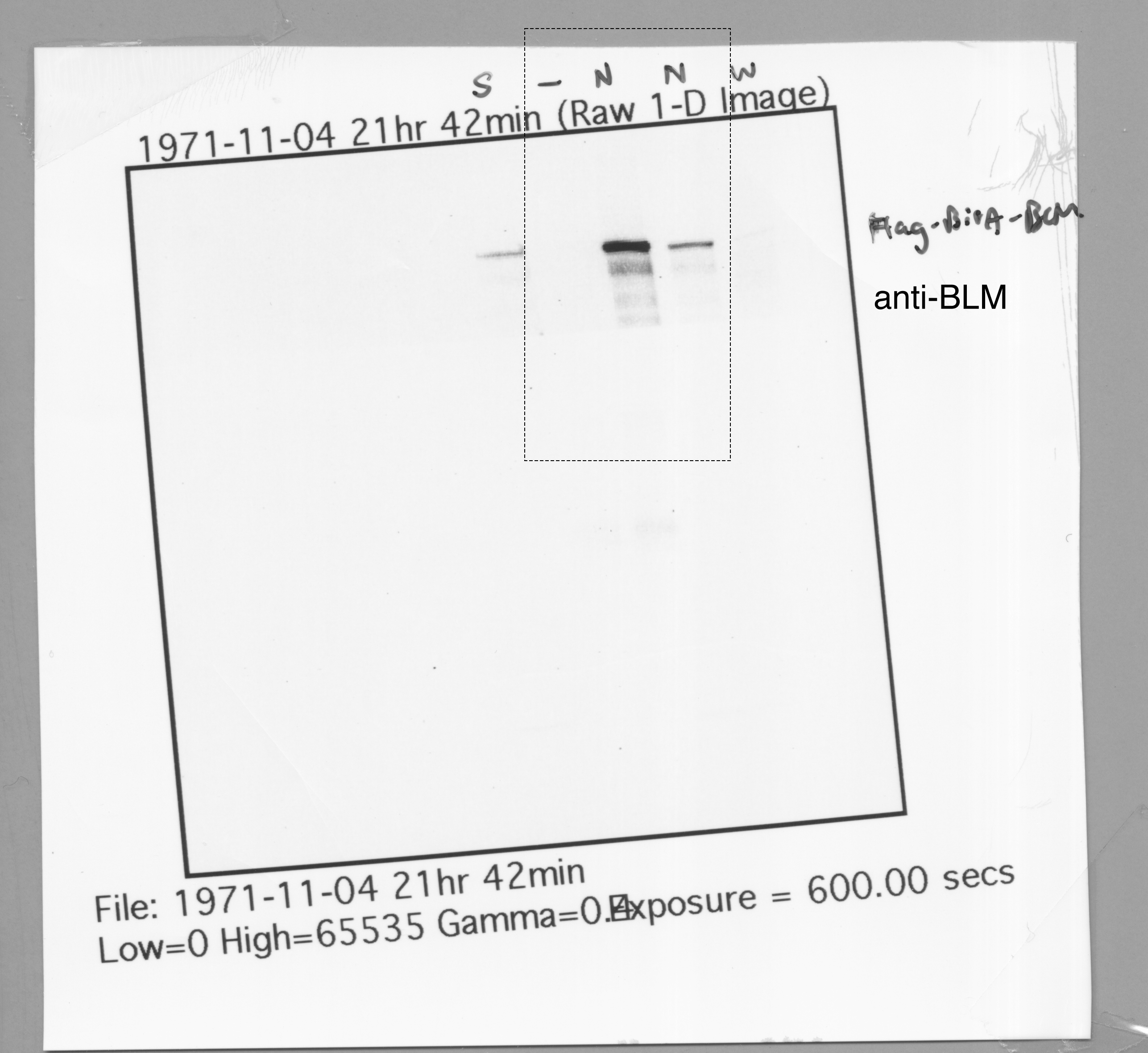

Supplement: Supplementary file 5 — Source data Fig. 4 [file 44319_2025_374_MOESM5_ESM.zip › Figure 4/SourceData4B/SourceDataForFigure4B.BLM.tif]

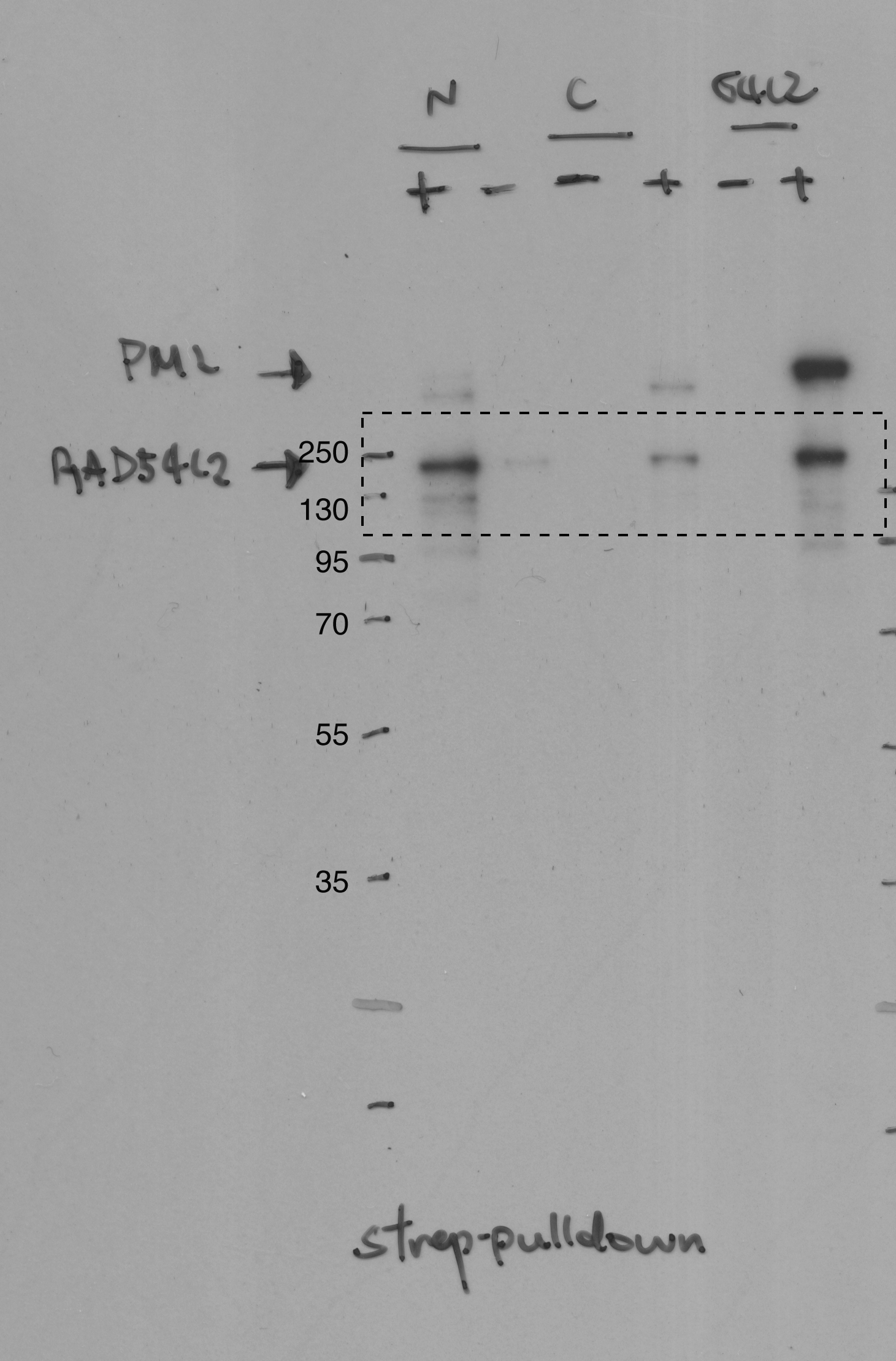

Supplement: Supplementary file 5 — Source data Fig. 4 [file 44319_2025_374_MOESM5_ESM.zip › Figure 4/SourceData4A/SourceDataForFigure4A.54L2.tif]

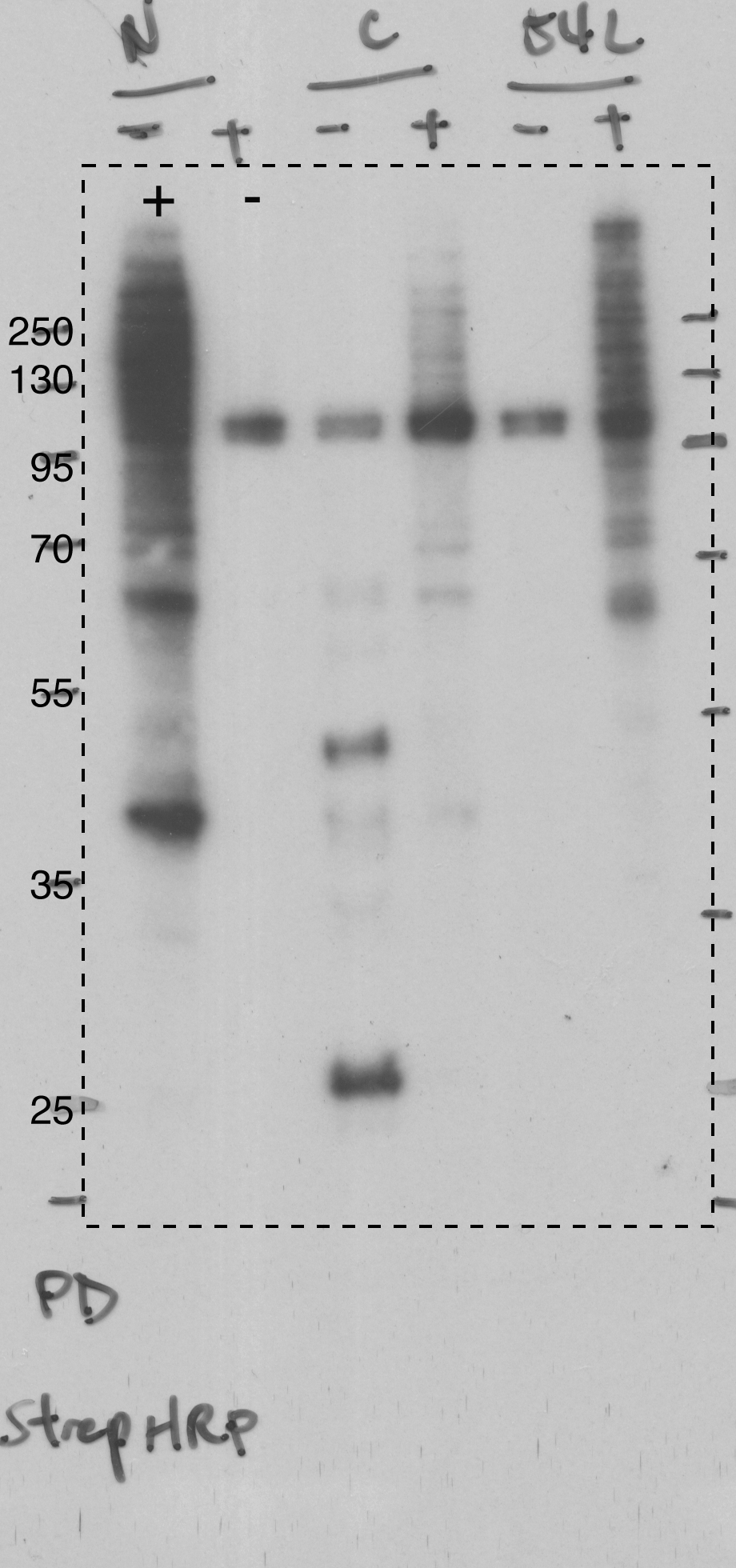

Supplement: Supplementary file 5 — Source data Fig. 4 [file 44319_2025_374_MOESM5_ESM.zip › Figure 4/SourceData4A/SourceDataForFigure4A.SAHRP.tif]

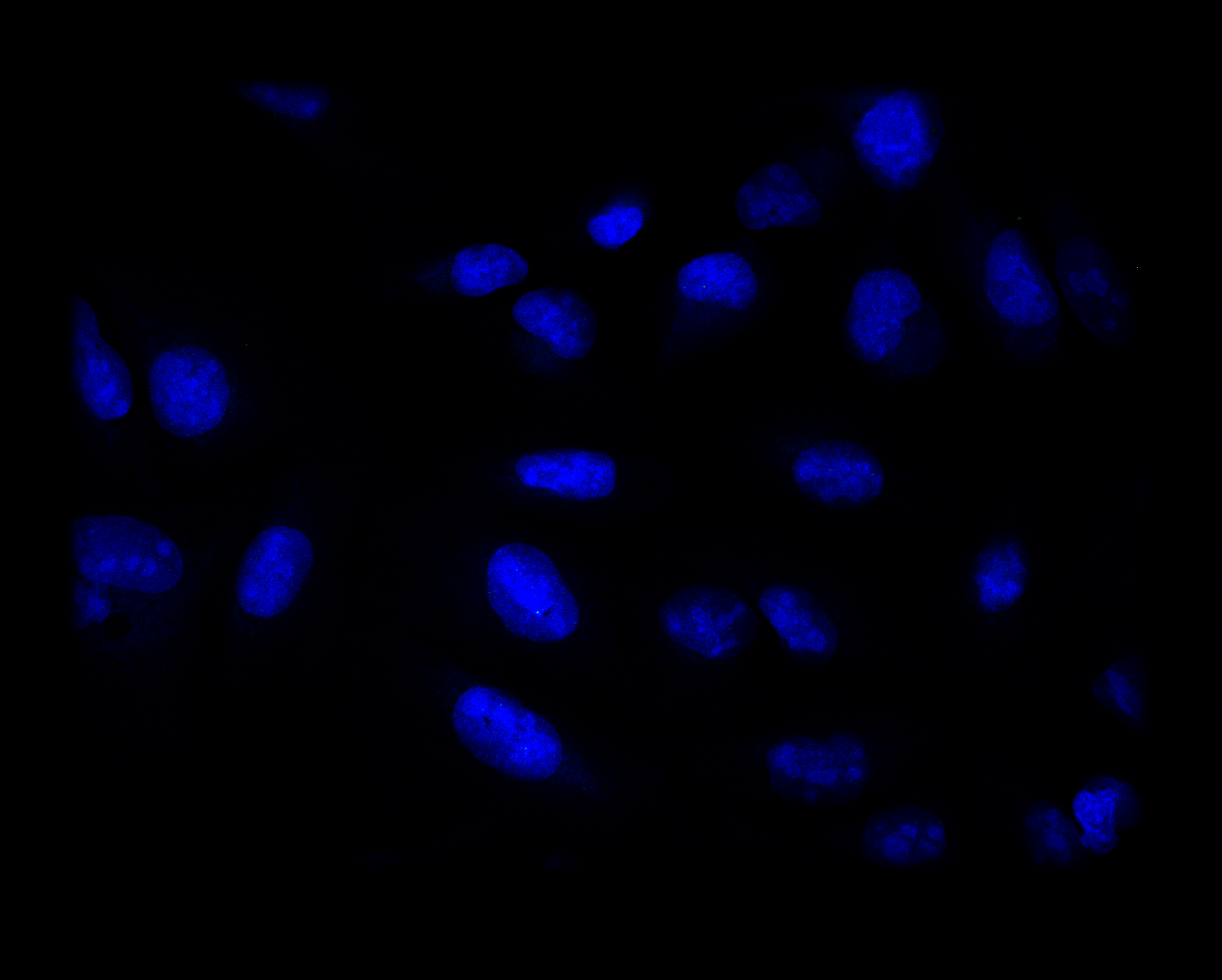

Supplement: Supplementary file 6 — Source data Fig. 5 [file 44319_2025_374_MOESM6_ESM.zip › Figure 5/SourceData5A/54L2KO1_BLM_HU_merge.tif]

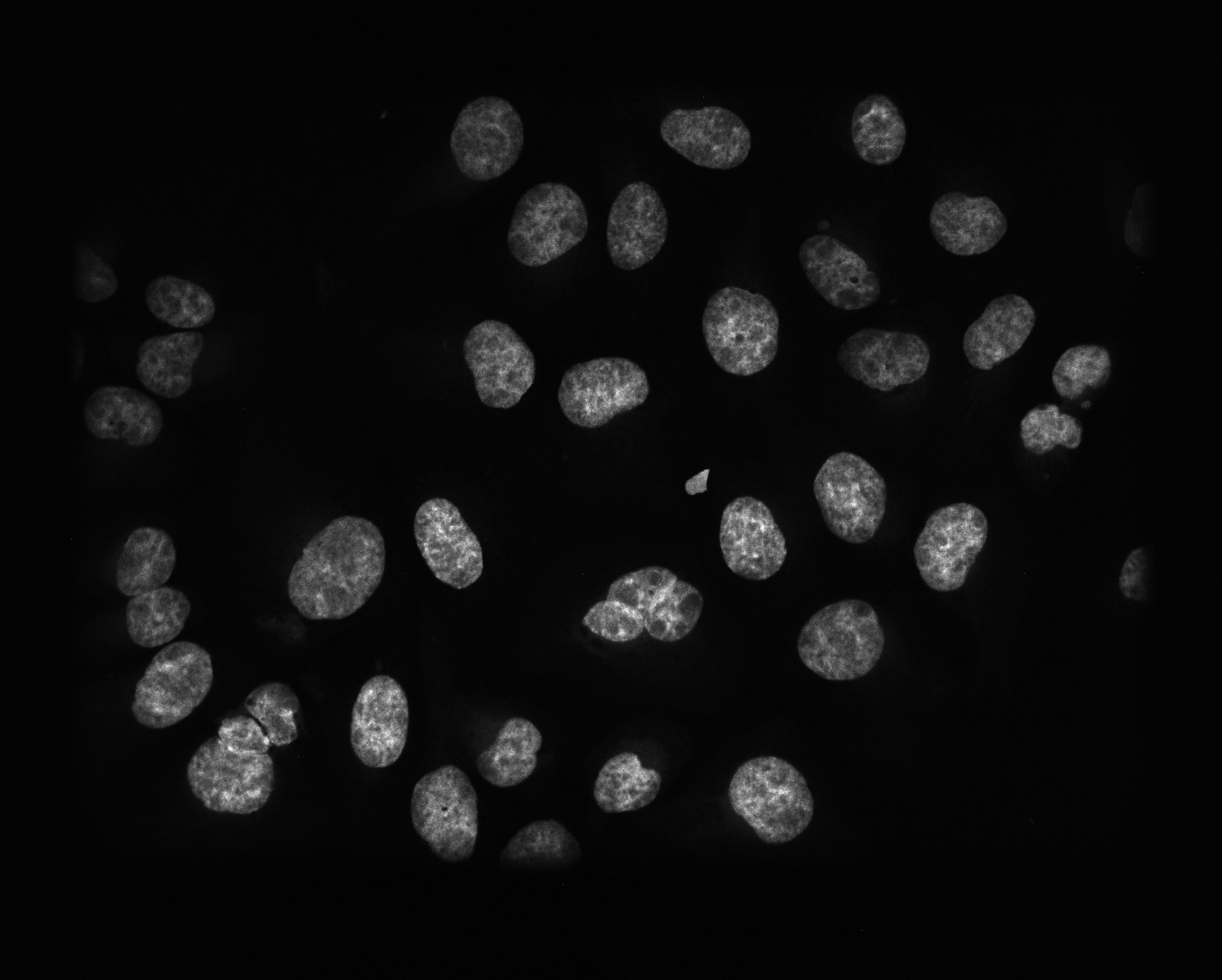

Supplement: Supplementary file 6 — Source data Fig. 5 [file 44319_2025_374_MOESM6_ESM.zip › Figure 5/SourceData5A/U2OS_BLM_UT_DAPI.tif]

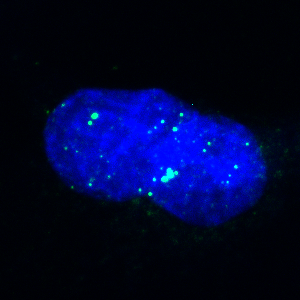

Supplement: Supplementary file 6 — Source data Fig. 5 [file 44319_2025_374_MOESM6_ESM.zip › Figure 5/SourceData5A/U2OS_BLM_HU_merge_inset.tif]

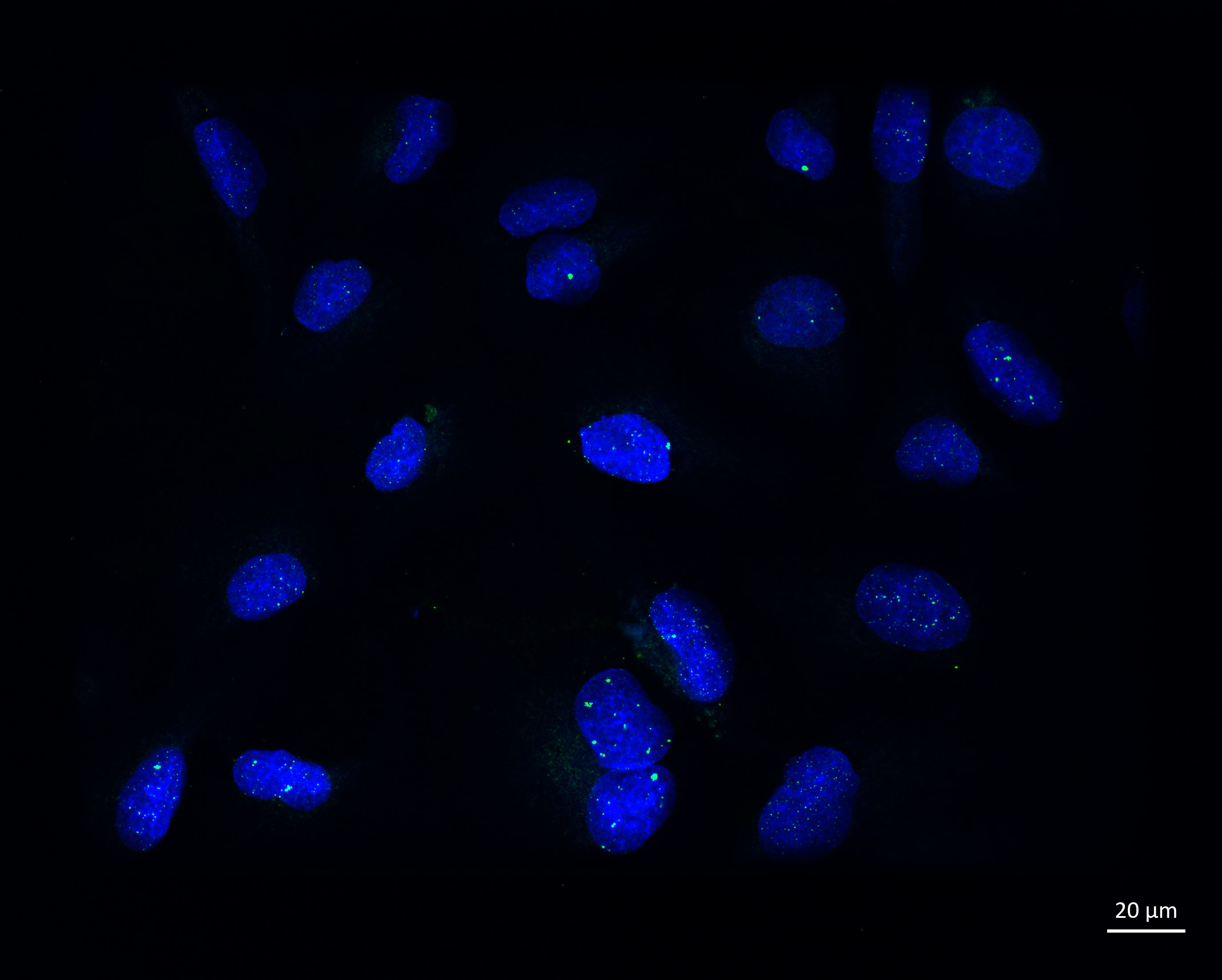

Supplement: Supplementary file 6 — Source data Fig. 5 [file 44319_2025_374_MOESM6_ESM.zip › Figure 5/SourceData5A/U2OS_BLM_HU_merge.tif]

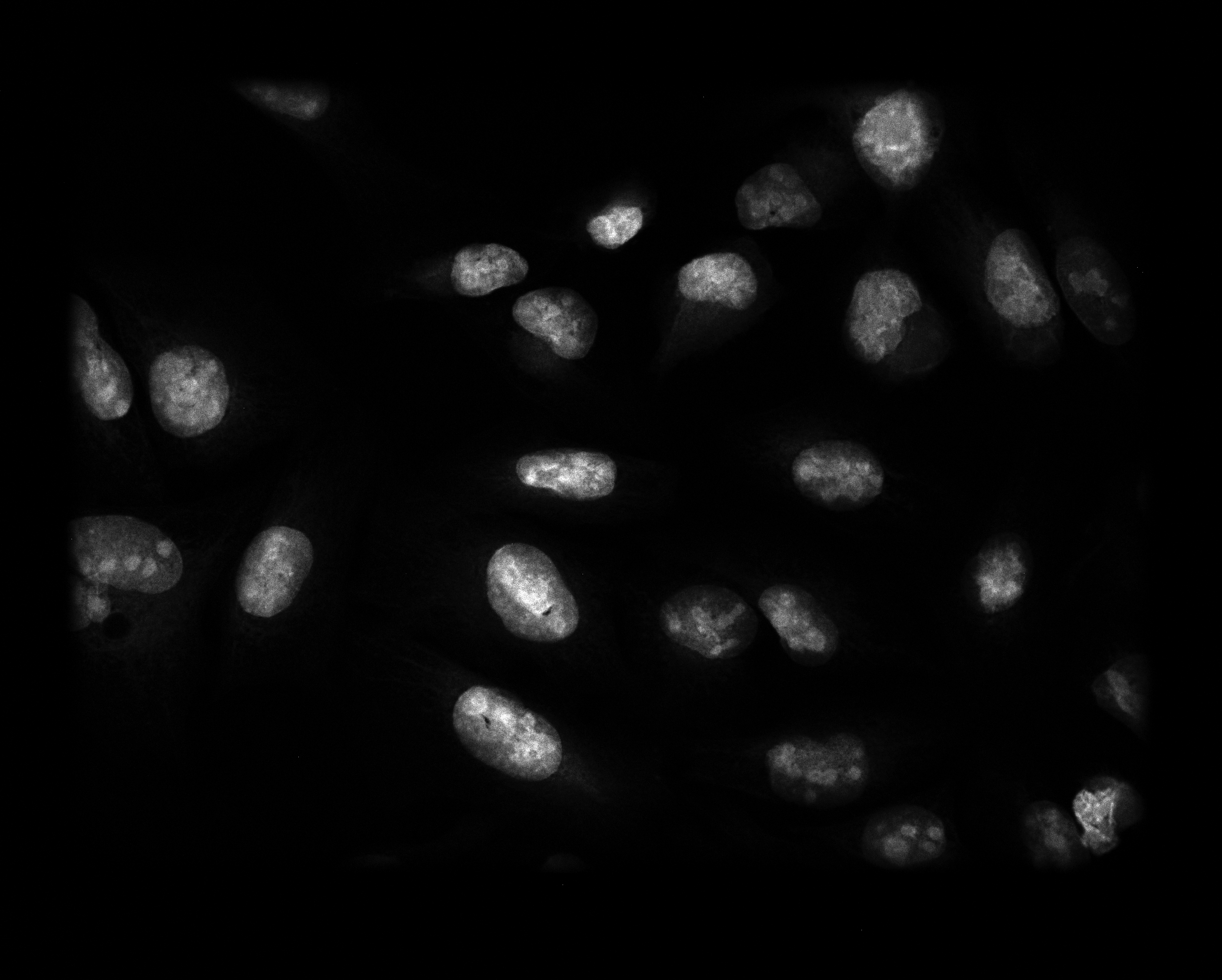

Supplement: Supplementary file 6 — Source data Fig. 5 [file 44319_2025_374_MOESM6_ESM.zip › Figure 5/SourceData5A/54L2KO1_BLM_HU_DAPI.tif]

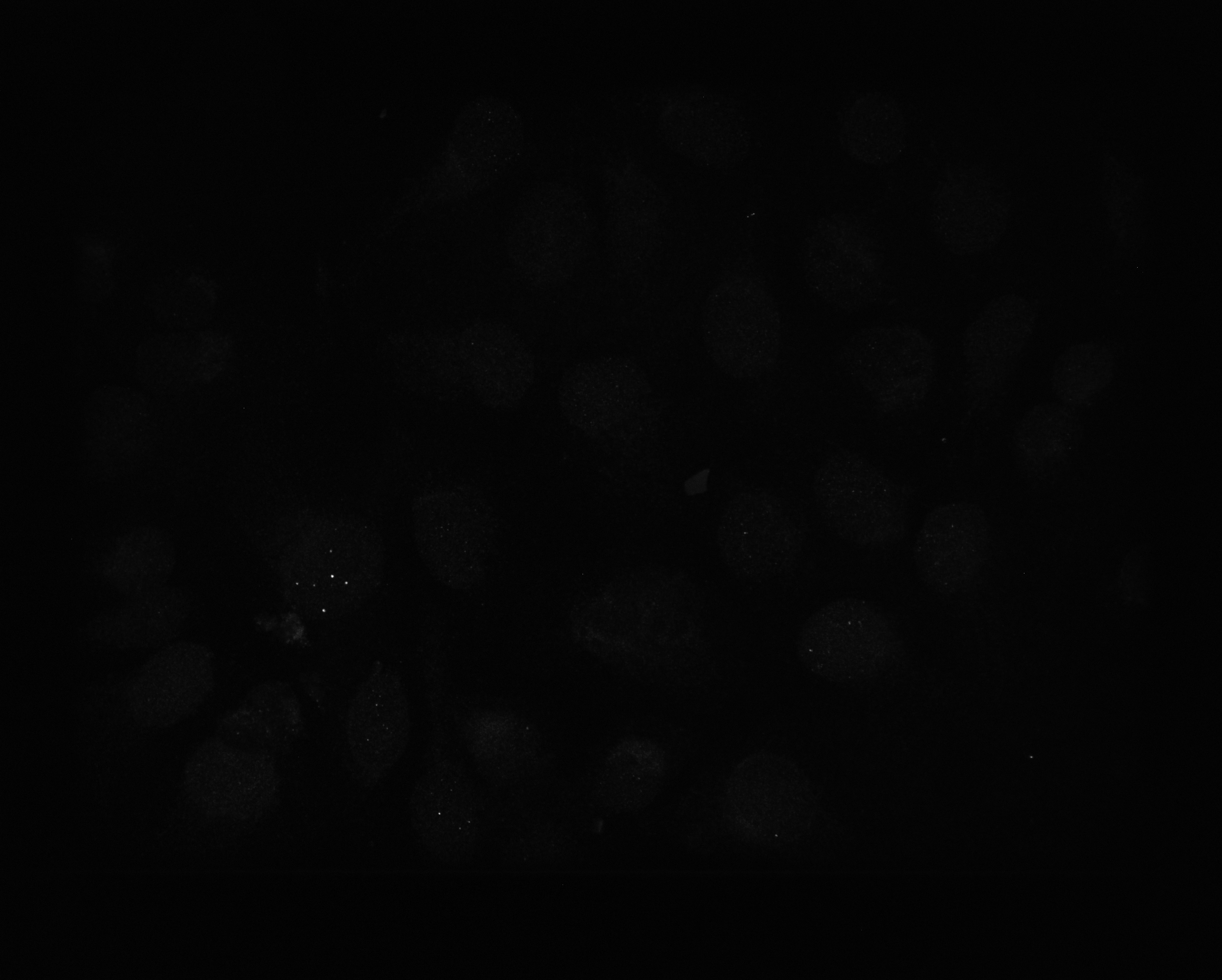

Supplement: Supplementary file 6 — Source data Fig. 5 [file 44319_2025_374_MOESM6_ESM.zip › Figure 5/SourceData5A/U2OS_BLM_UT_Alexa546.tif]

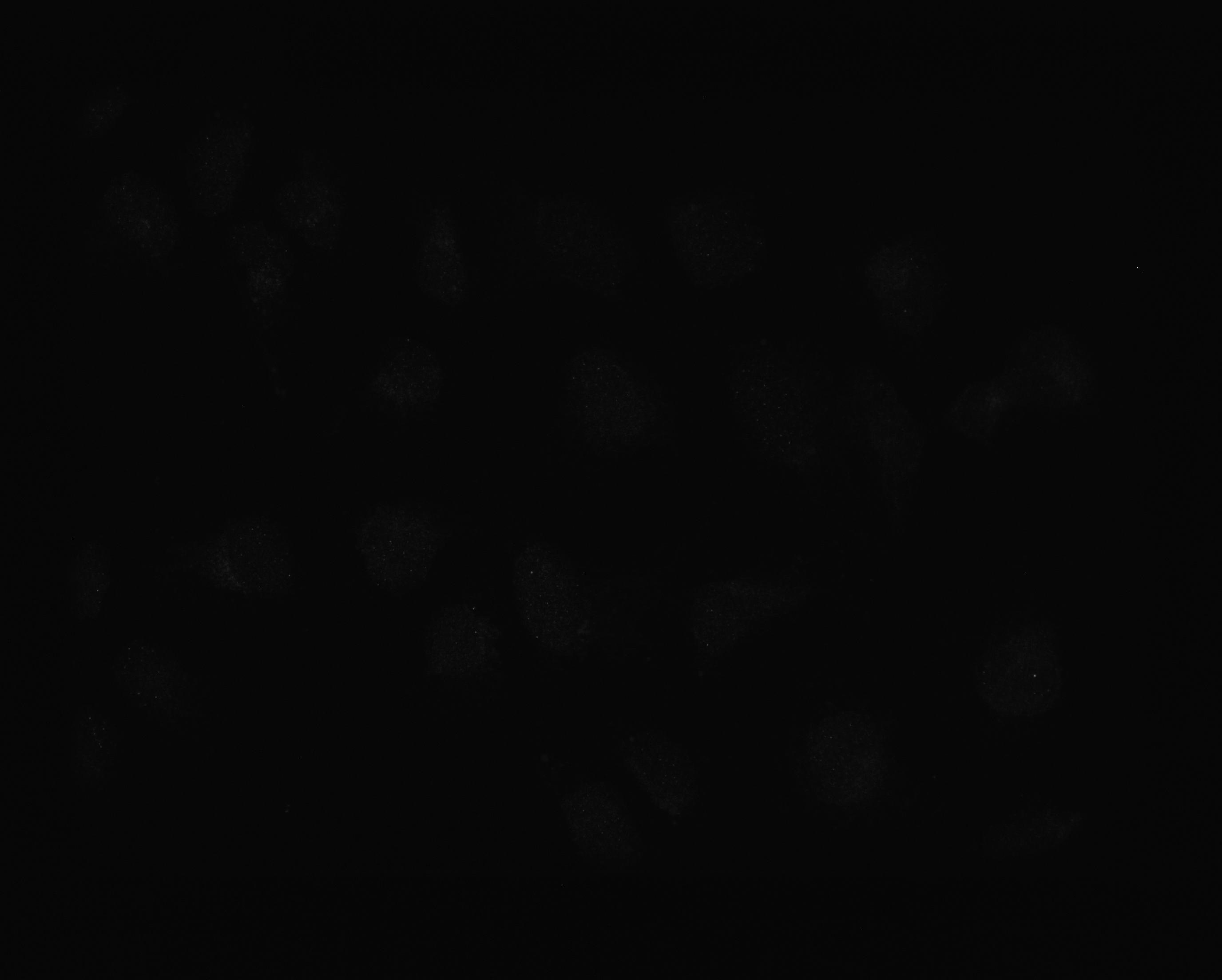

Supplement: Supplementary file 6 — Source data Fig. 5 [file 44319_2025_374_MOESM6_ESM.zip › Figure 5/SourceData5A/54L2KO1_BLM_UT_Alexa546.tif]

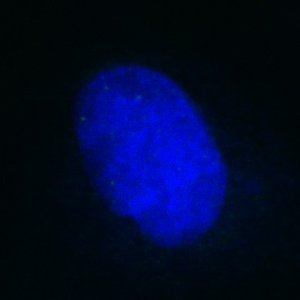

Supplement: Supplementary file 6 — Source data Fig. 5 [file 44319_2025_374_MOESM6_ESM.zip › Figure 5/SourceData5A/54L2KO1_BLM_UT_merge_inset.tif]

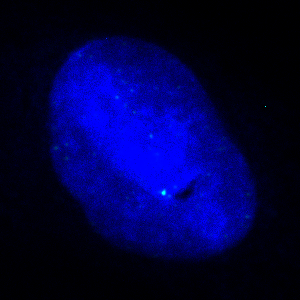

Supplement: Supplementary file 6 — Source data Fig. 5 [file 44319_2025_374_MOESM6_ESM.zip › Figure 5/SourceData5A/54L2KO1_BLM_HU_merge_inset.tif]

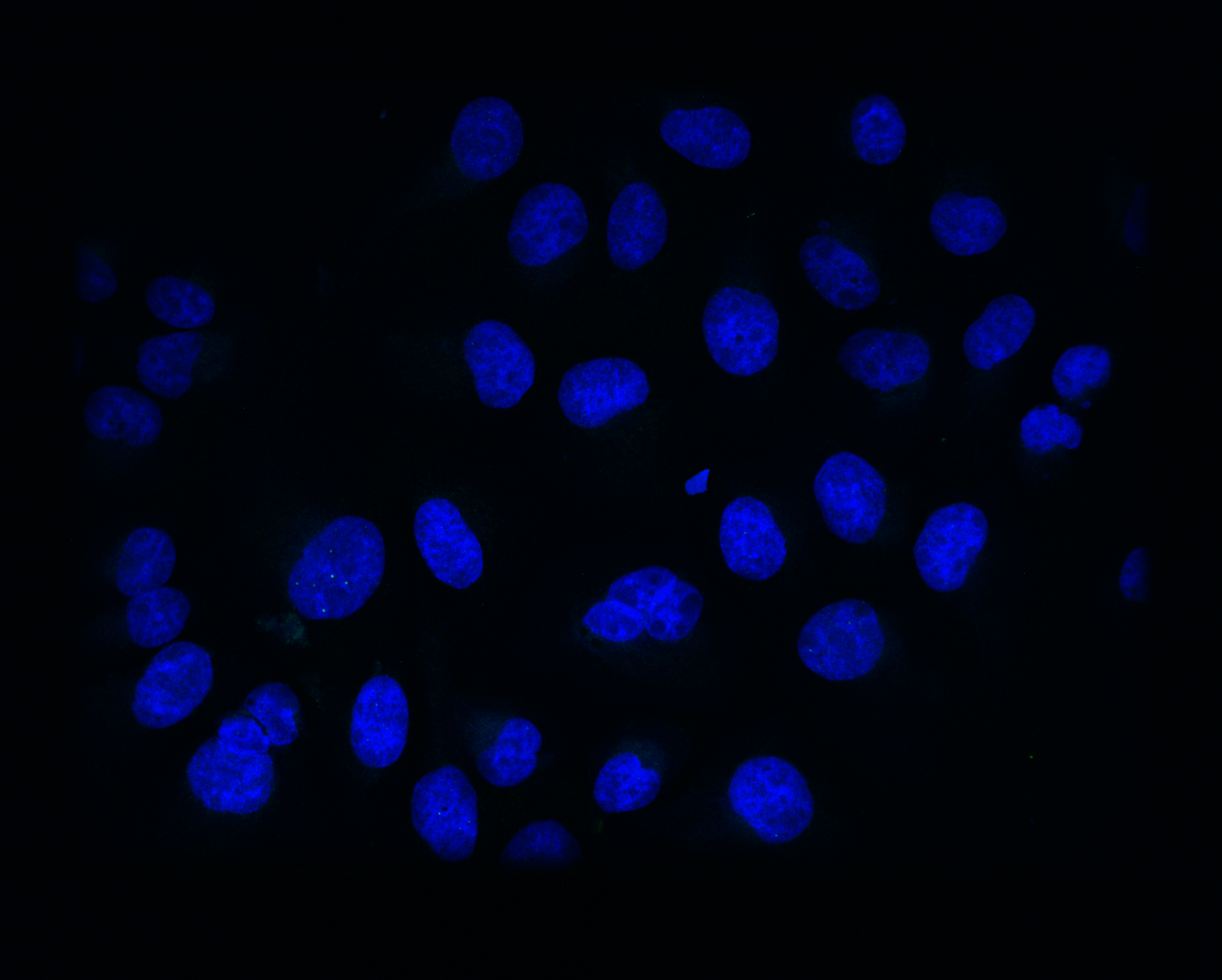

Supplement: Supplementary file 6 — Source data Fig. 5 [file 44319_2025_374_MOESM6_ESM.zip › Figure 5/SourceData5A/U2OS_BLM_UT_merge.tif]

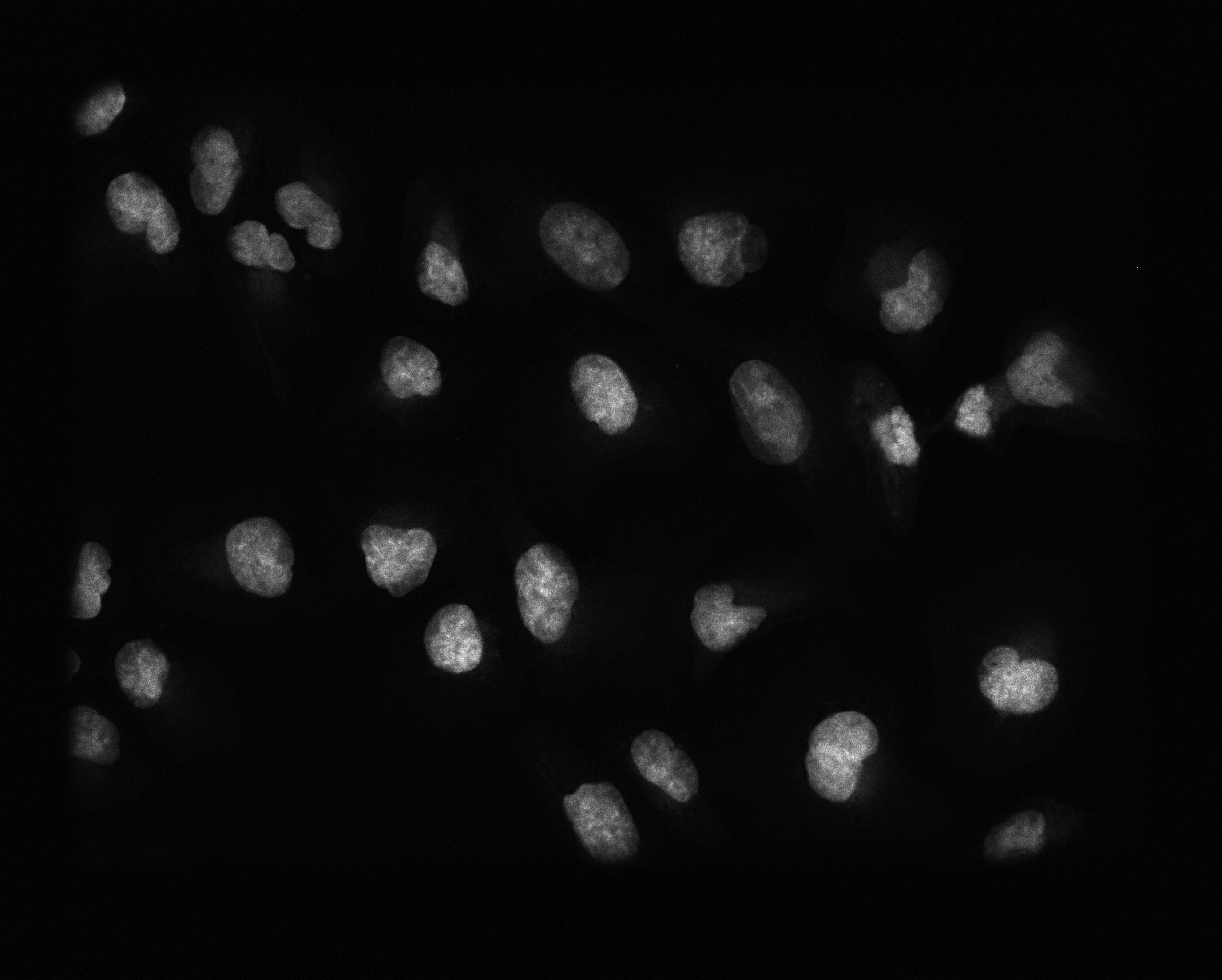

Supplement: Supplementary file 6 — Source data Fig. 5 [file 44319_2025_374_MOESM6_ESM.zip › Figure 5/SourceData5A/54L2KO1_BLM_UT_DAPI.tif]

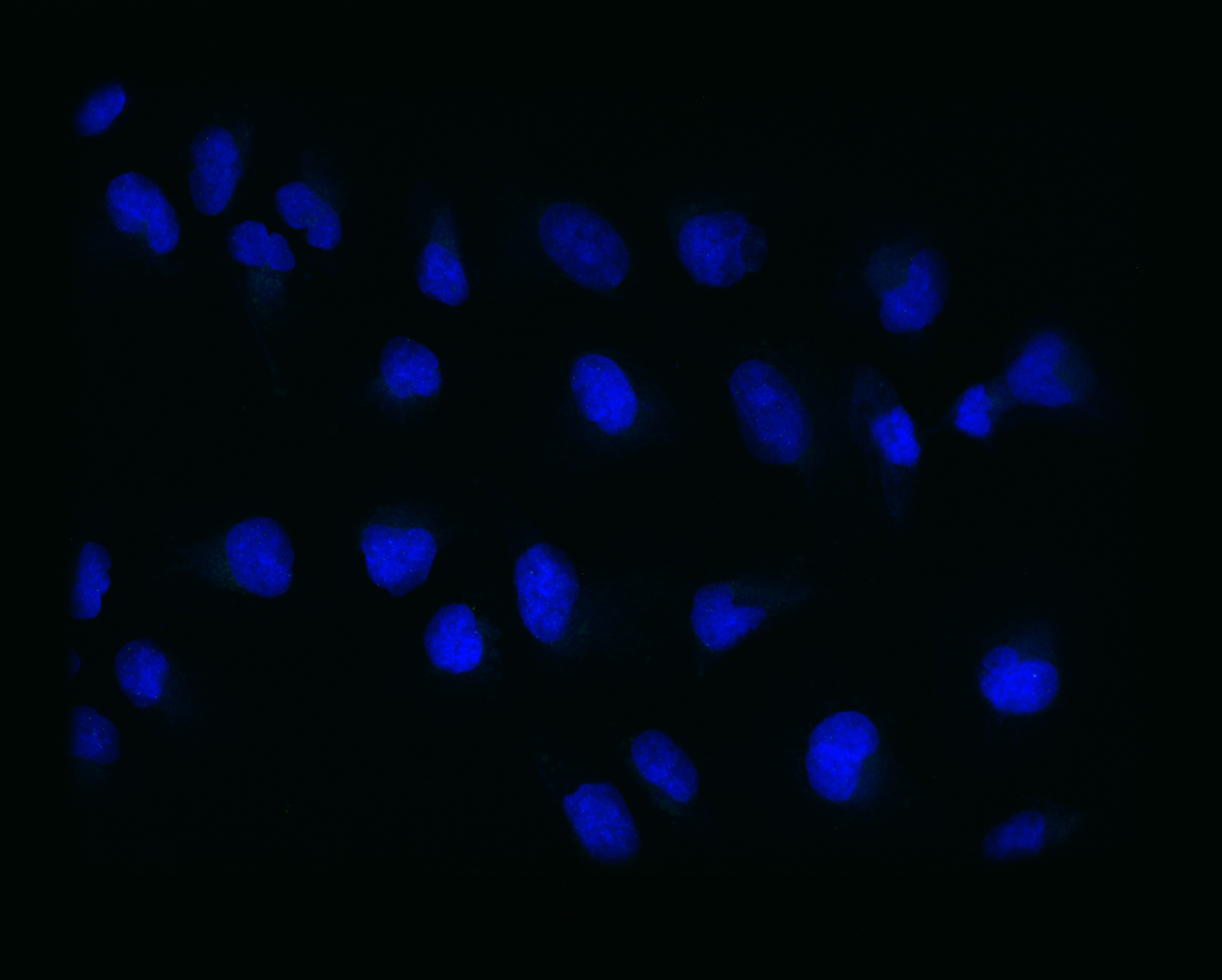

Supplement: Supplementary file 6 — Source data Fig. 5 [file 44319_2025_374_MOESM6_ESM.zip › Figure 5/SourceData5A/54L2KO1_BLM_UT_merge.tif]

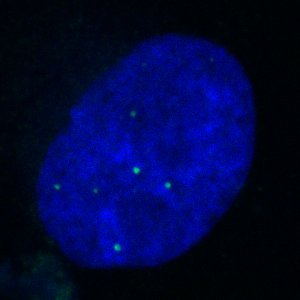

Supplement: Supplementary file 6 — Source data Fig. 5 [file 44319_2025_374_MOESM6_ESM.zip › Figure 5/SourceData5A/U2OS_BLM_UT_merge_inset.tif]

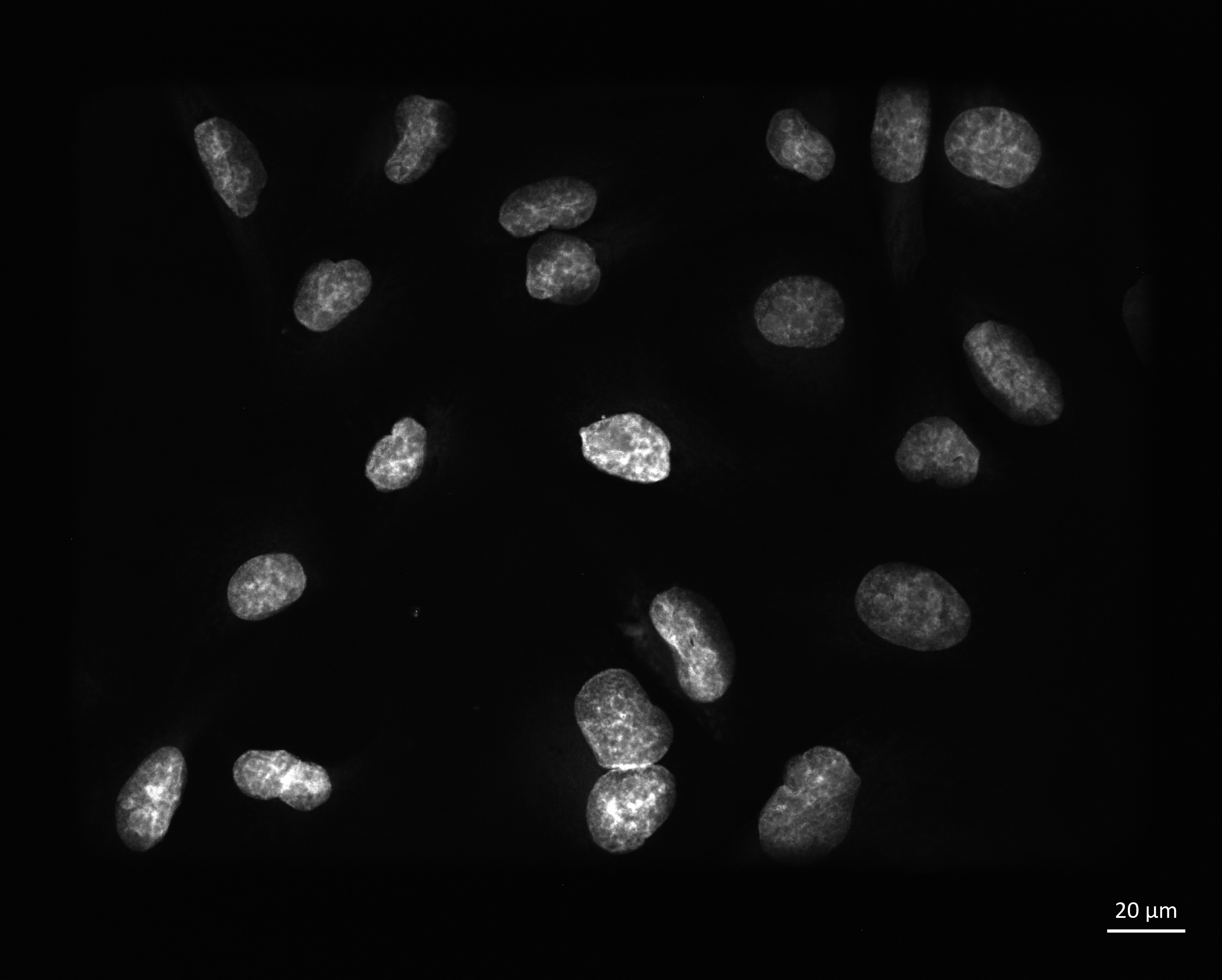

Supplement: Supplementary file 6 — Source data Fig. 5 [file 44319_2025_374_MOESM6_ESM.zip › Figure 5/SourceData5A/U2OS_BLM_HU_DAPI.tif]

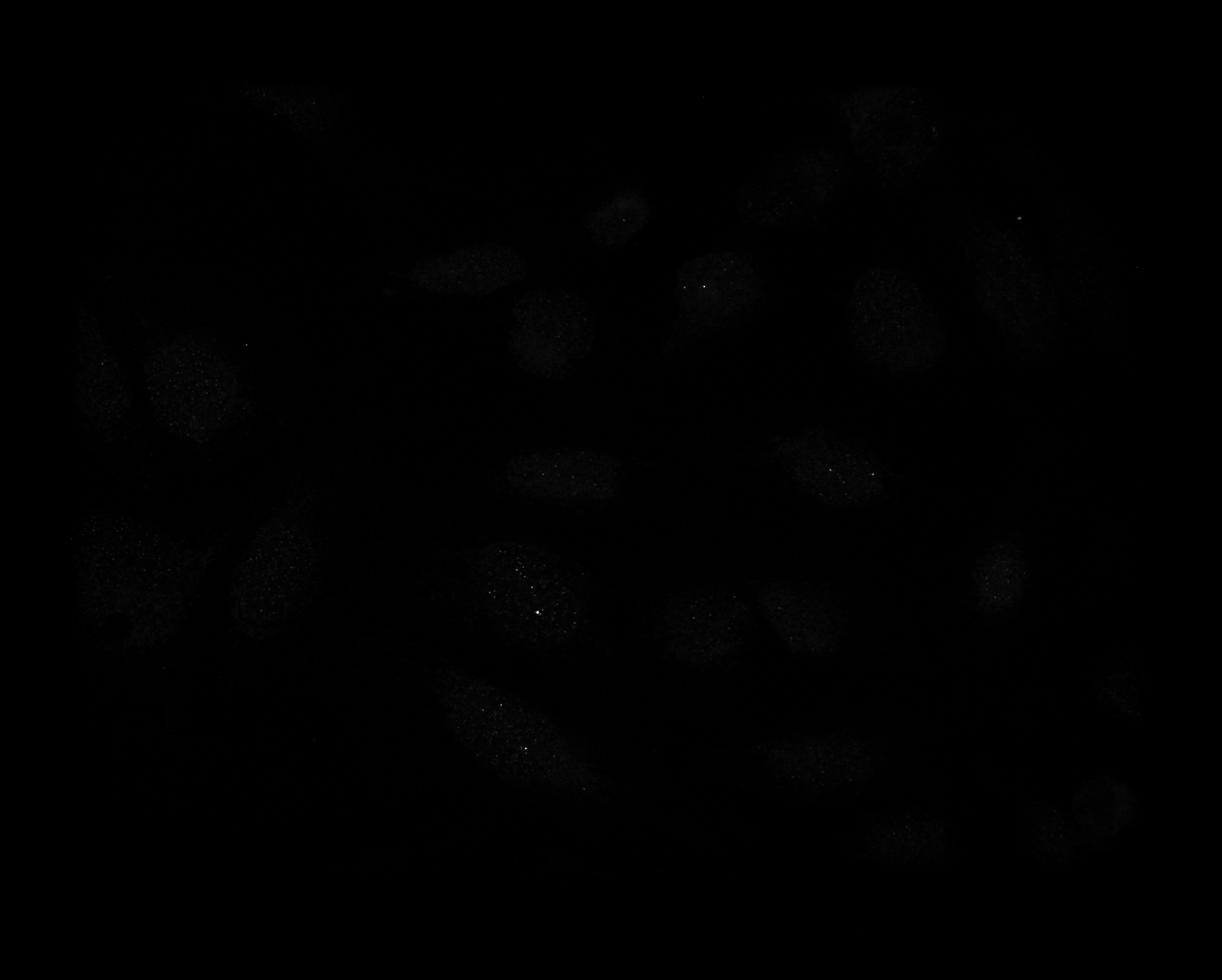

Supplement: Supplementary file 6 — Source data Fig. 5 [file 44319_2025_374_MOESM6_ESM.zip › Figure 5/SourceData5A/54L2KO1_BLM_HU_Alexa546.tif]

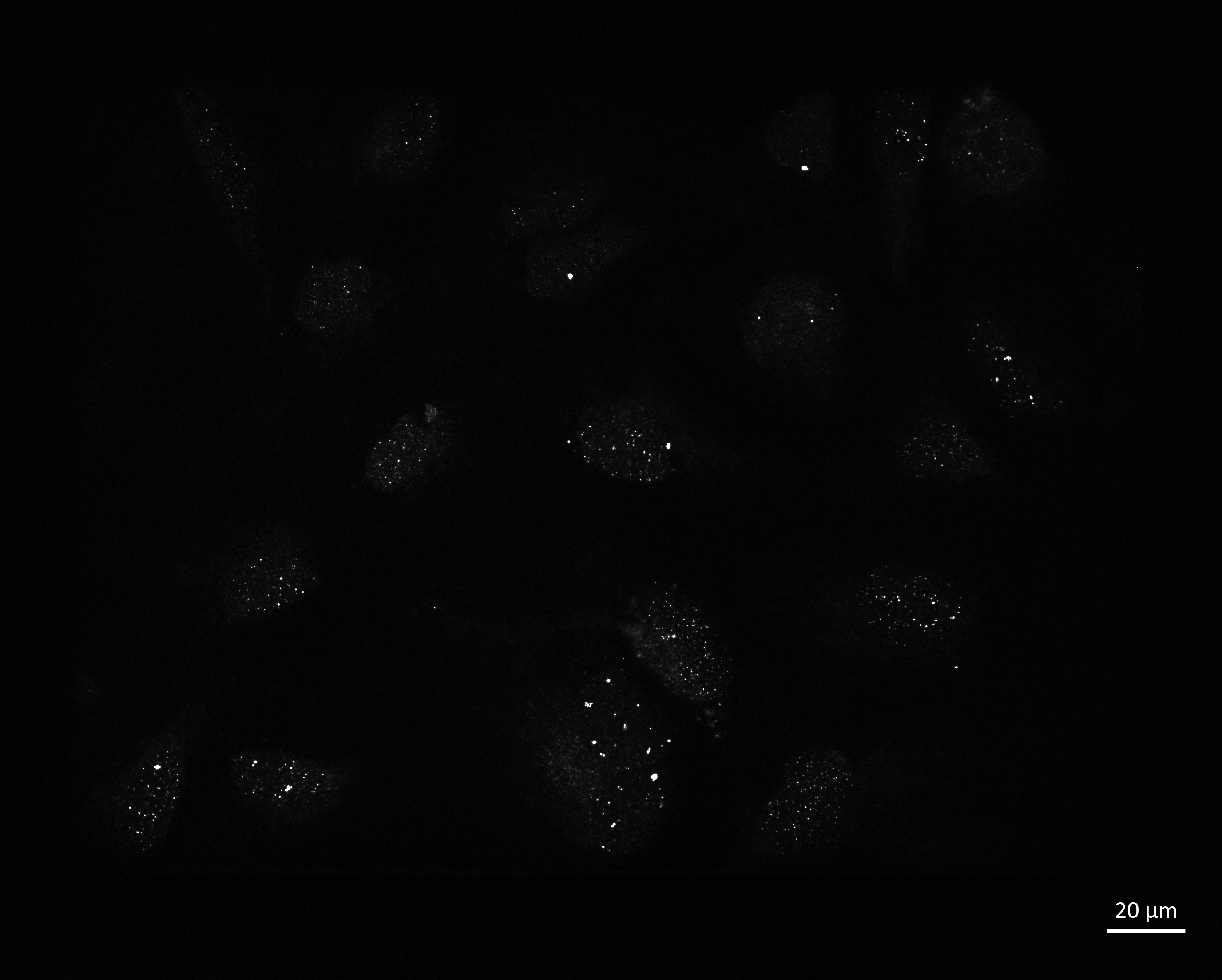

Supplement: Supplementary file 6 — Source data Fig. 5 [file 44319_2025_374_MOESM6_ESM.zip › Figure 5/SourceData5A/U2OS_BLM_HU_Alexa546.tif]

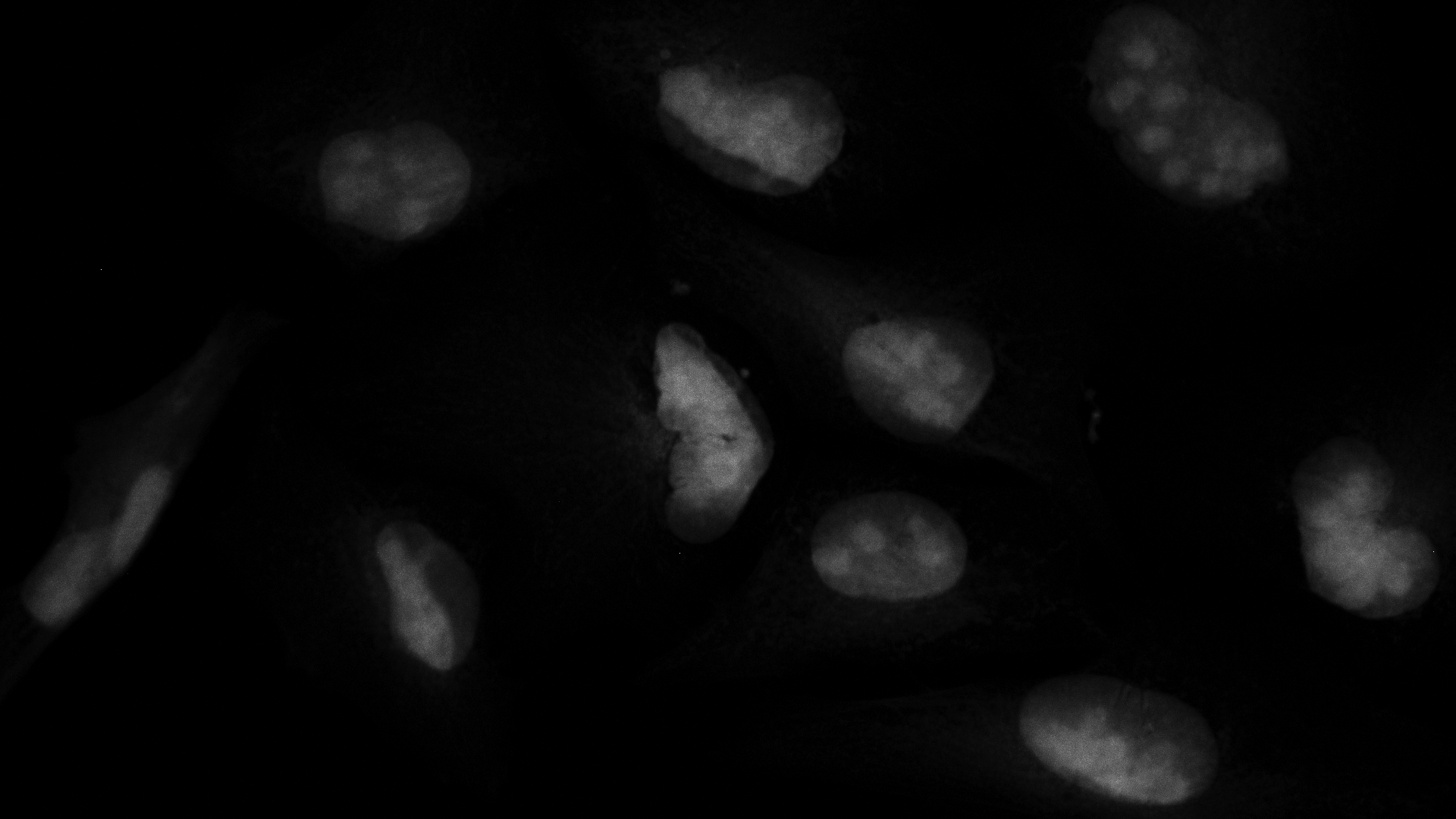

Supplement: Supplementary file 6 — Source data Fig. 5 [file 44319_2025_374_MOESM6_ESM.zip › Figure 5/SourceData5E/RAD54L2KO1_RAD51_HU_DAPI.tif]

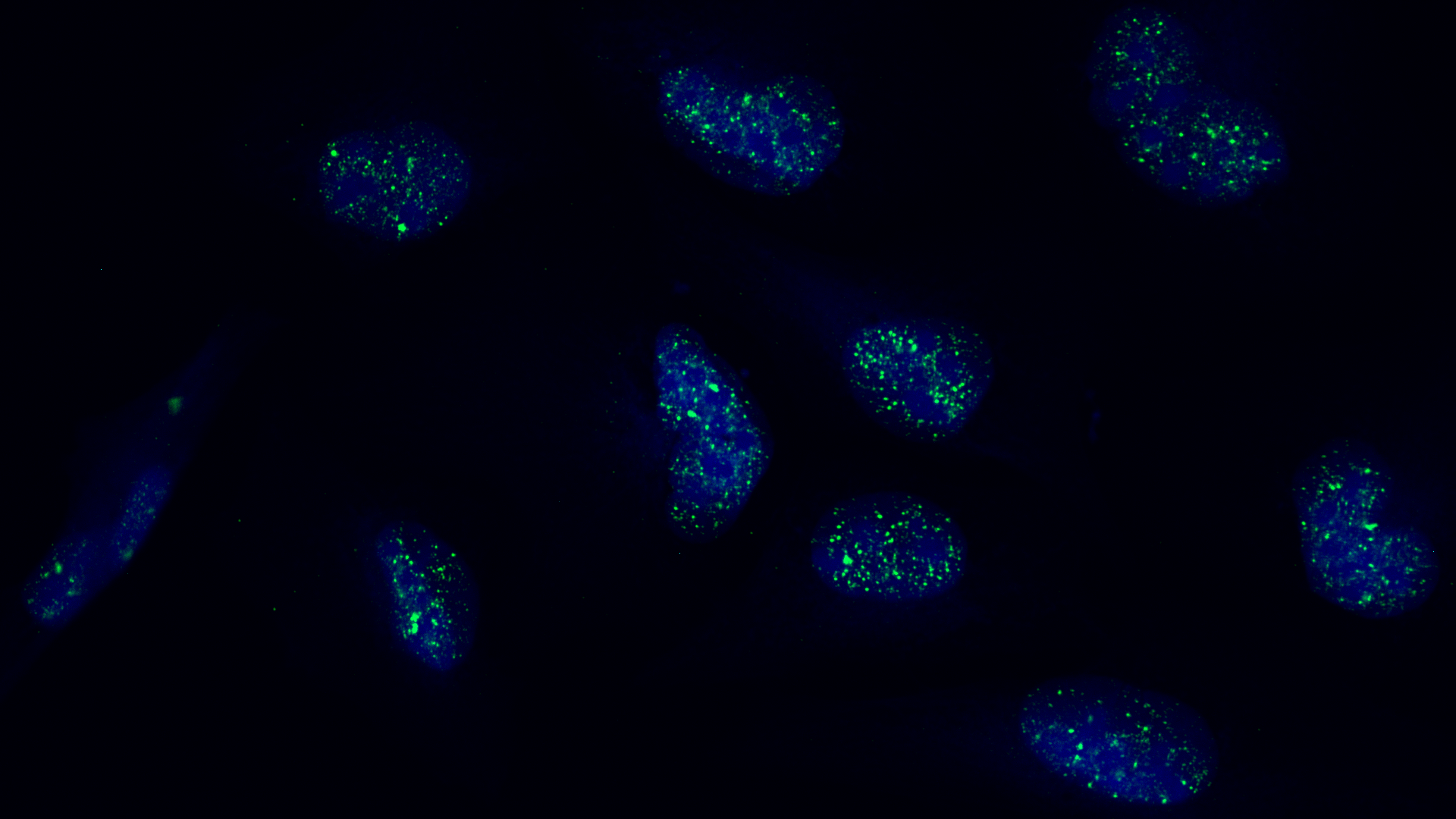

Supplement: Supplementary file 6 — Source data Fig. 5 [file 44319_2025_374_MOESM6_ESM.zip › Figure 5/SourceData5E/RAD54L2KO1_RAD51_HU_merge.tif]

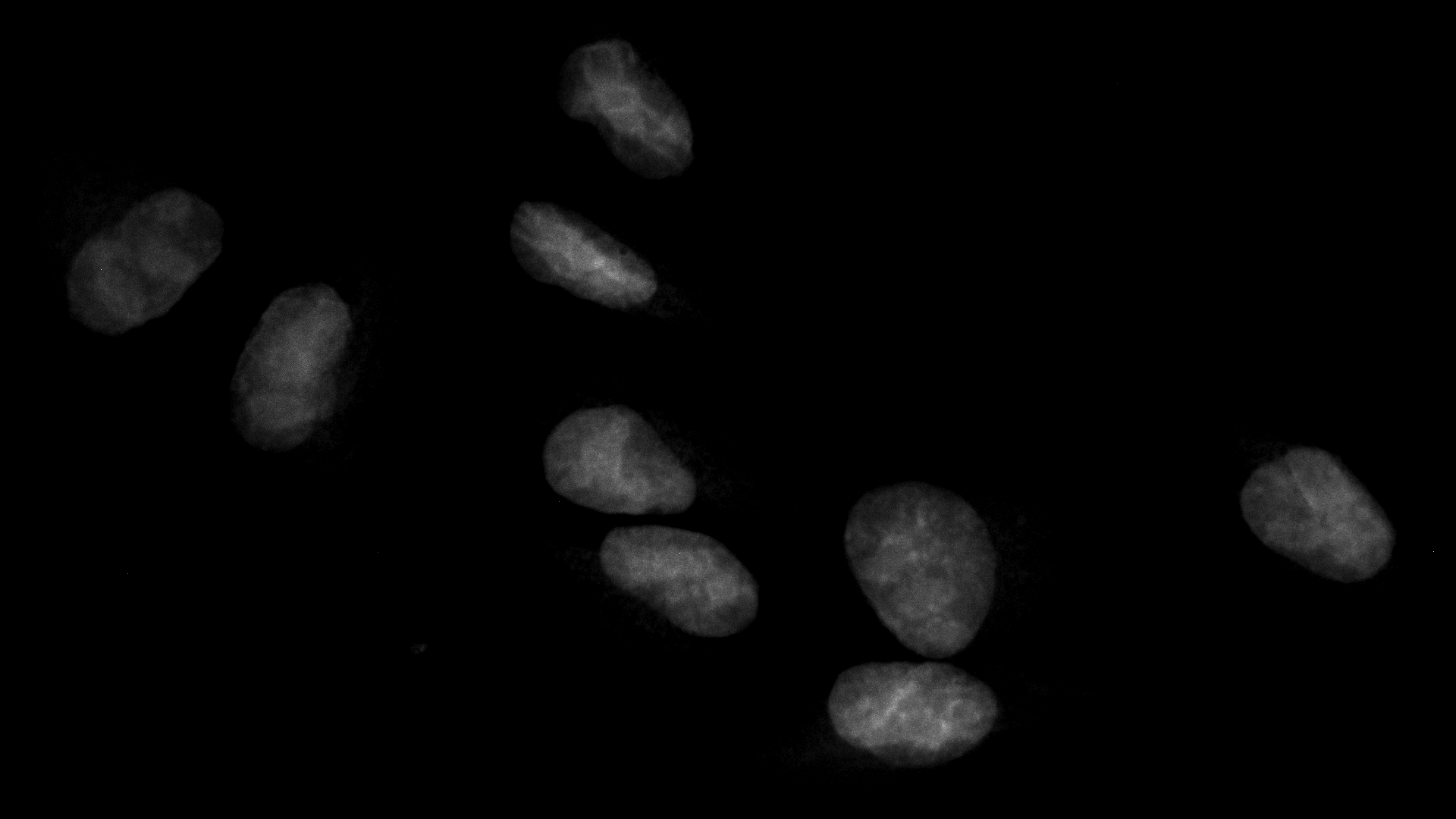

Supplement: Supplementary file 6 — Source data Fig. 5 [file 44319_2025_374_MOESM6_ESM.zip › Figure 5/SourceData5E/U2OS_RAD51_HU_DAPI.tif]

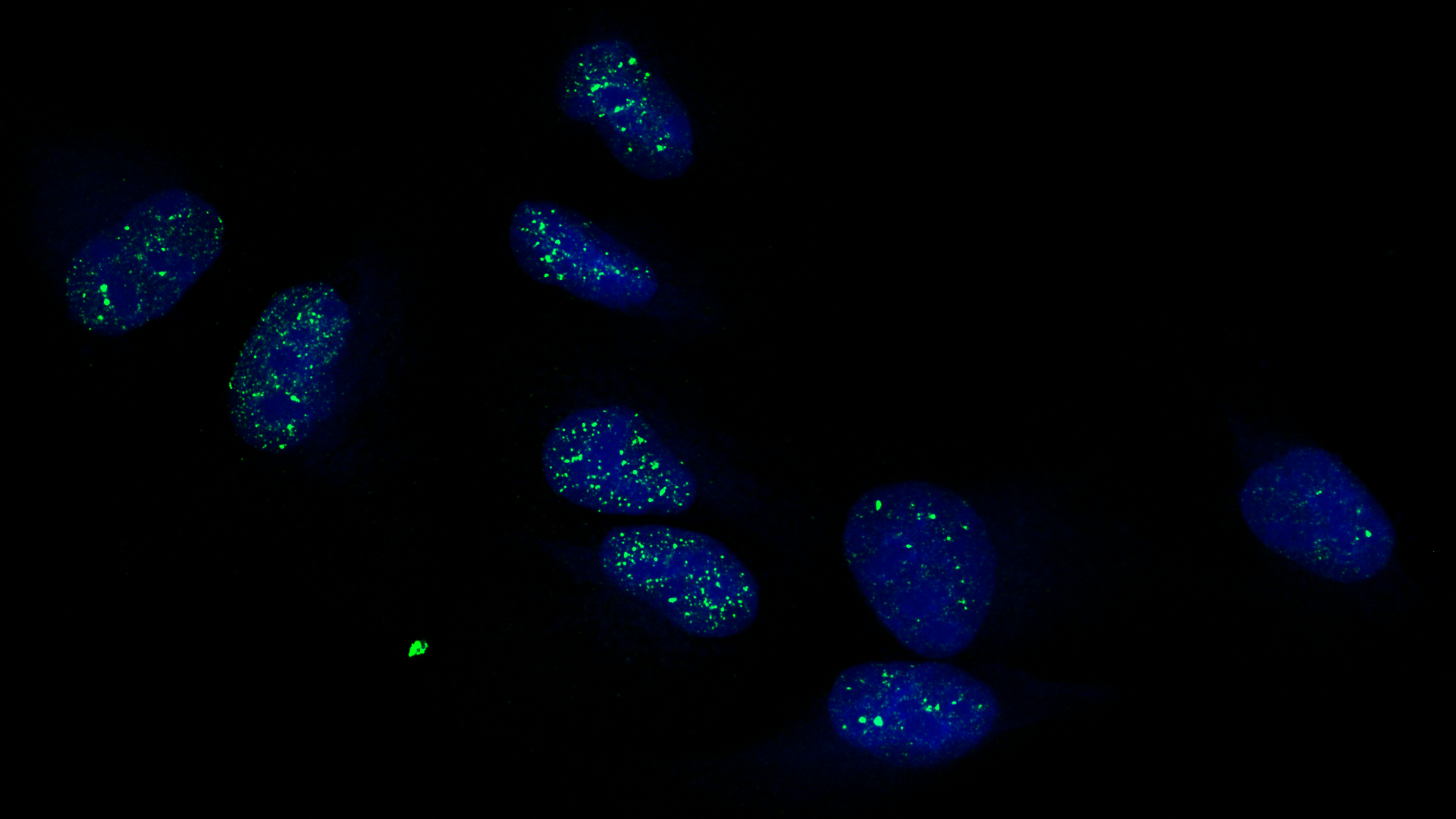

Supplement: Supplementary file 6 — Source data Fig. 5 [file 44319_2025_374_MOESM6_ESM.zip › Figure 5/SourceData5E/U2OS_RAD51_HU_merge.tif]

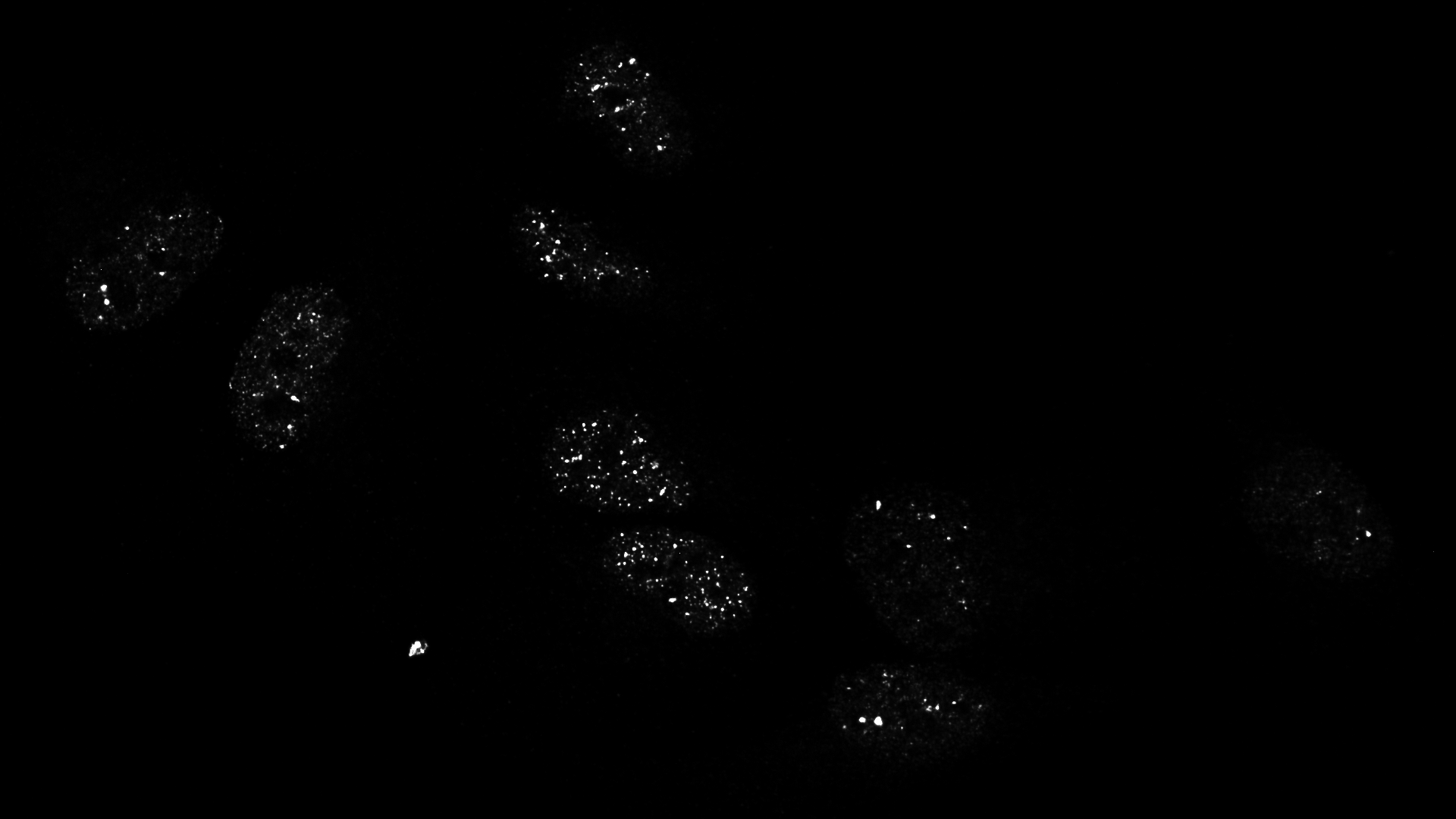

Supplement: Supplementary file 6 — Source data Fig. 5 [file 44319_2025_374_MOESM6_ESM.zip › Figure 5/SourceData5E/U2OS_RAD51_HU_Alexa546.tif]

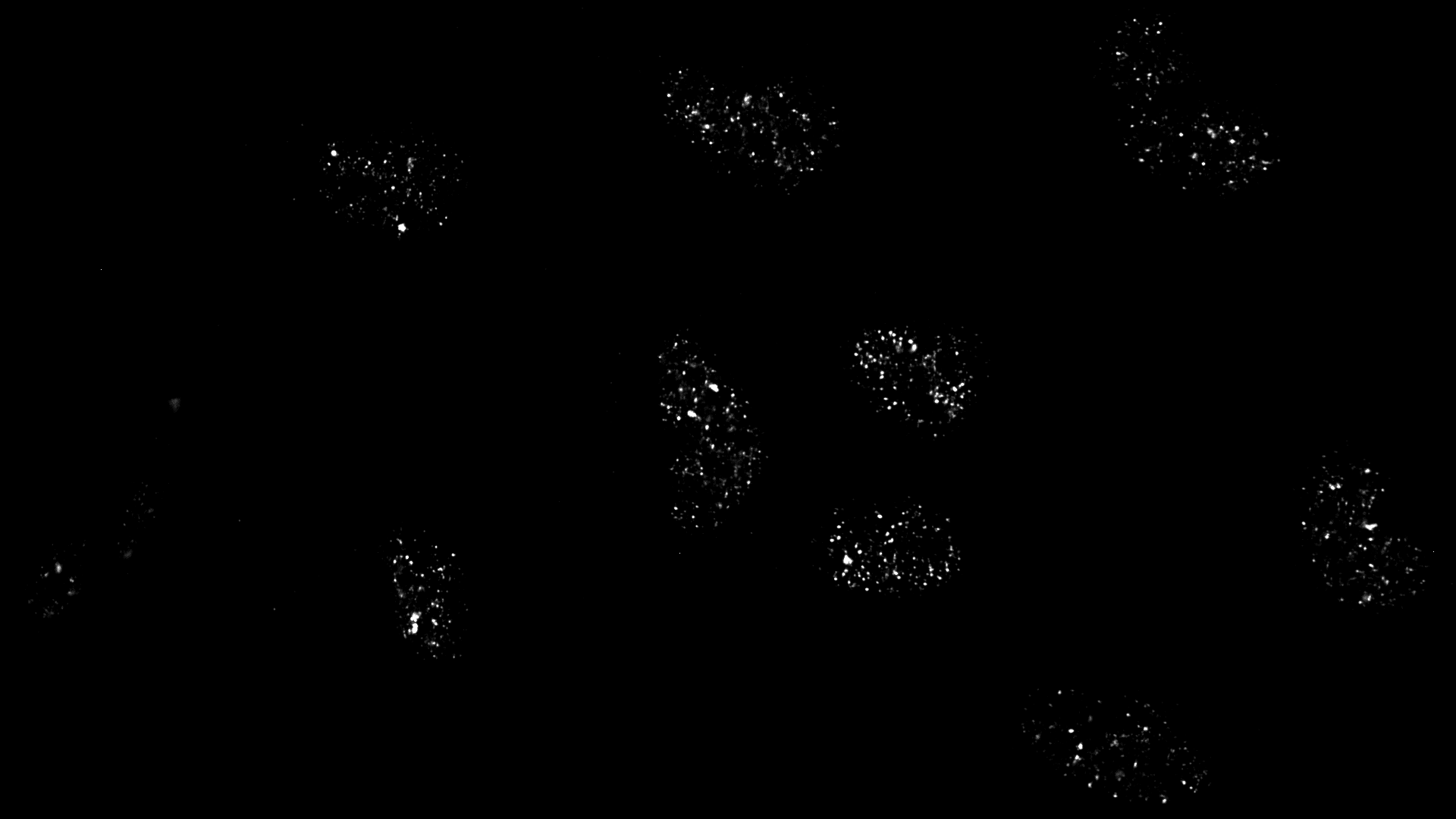

Supplement: Supplementary file 6 — Source data Fig. 5 [file 44319_2025_374_MOESM6_ESM.zip › Figure 5/SourceData5E/RAD54L2KO1_RAD51_HU_Alexa546.tif]

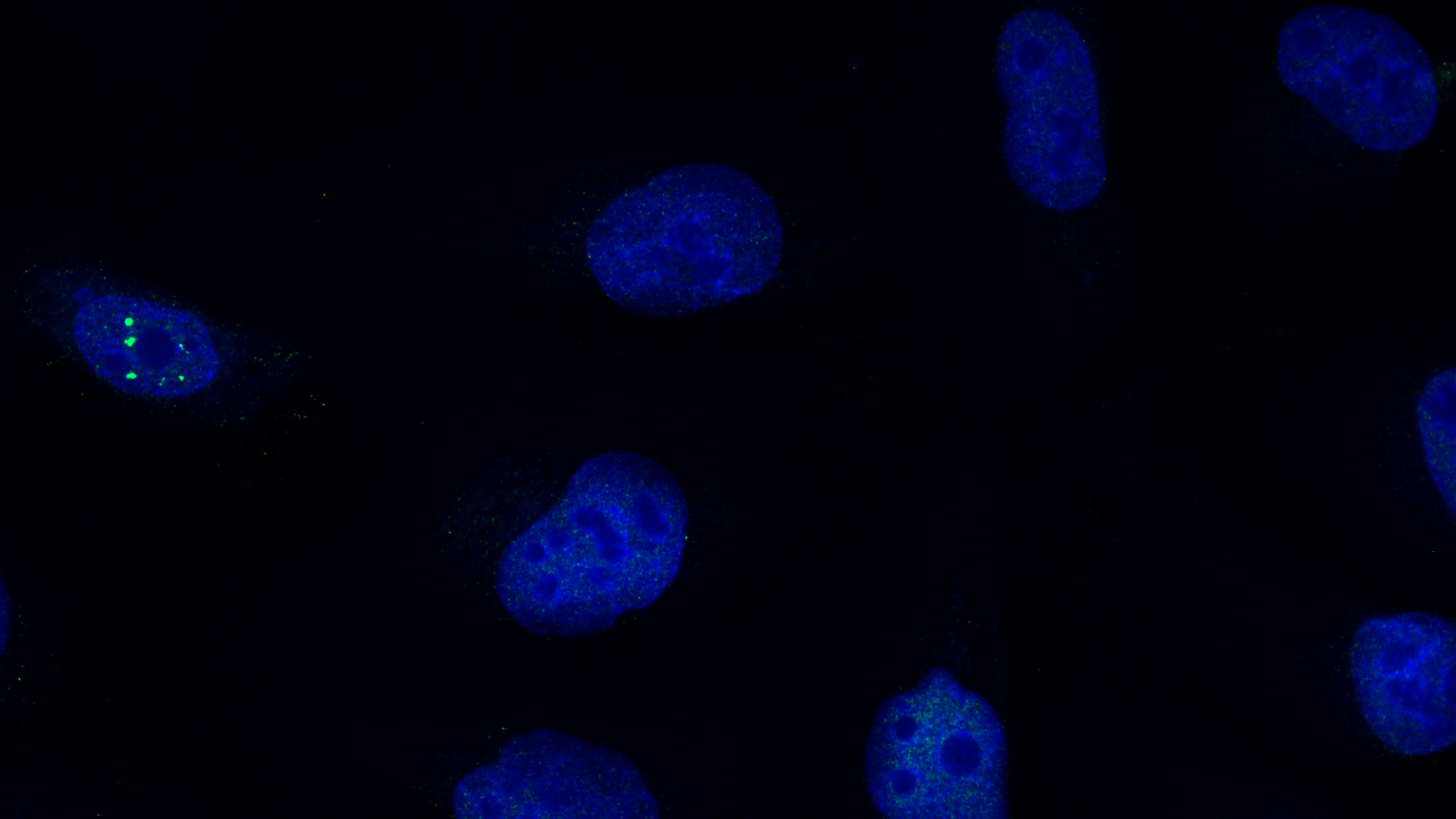

Supplement: Supplementary file 6 — Source data Fig. 5 [file 44319_2025_374_MOESM6_ESM.zip › Figure 5/SourceData5C/U2OS_MRE11_HU_merge.tif]

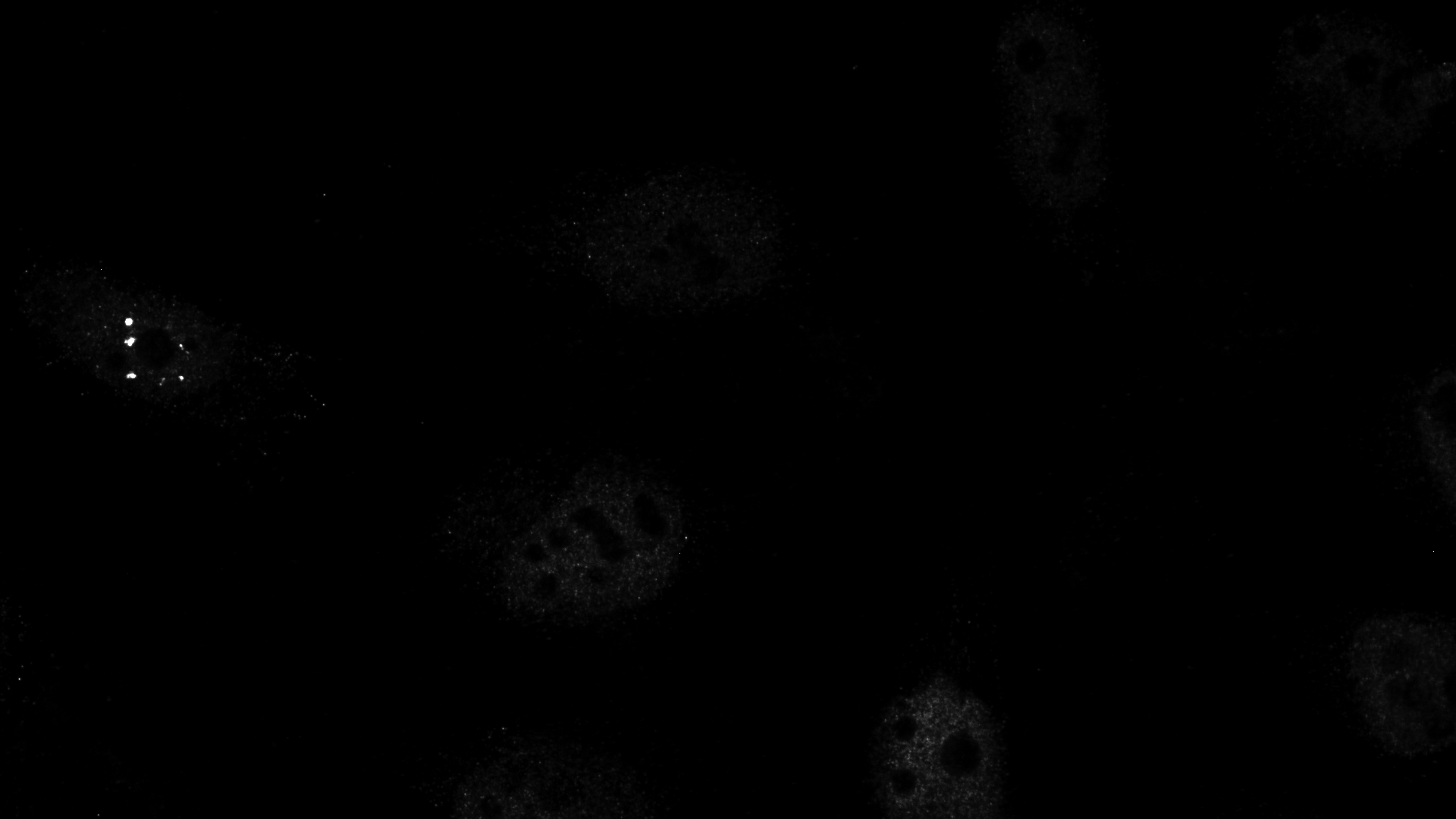

Supplement: Supplementary file 6 — Source data Fig. 5 [file 44319_2025_374_MOESM6_ESM.zip › Figure 5/SourceData5C/U2OS_MRE11_HU_Alexa546.tif]

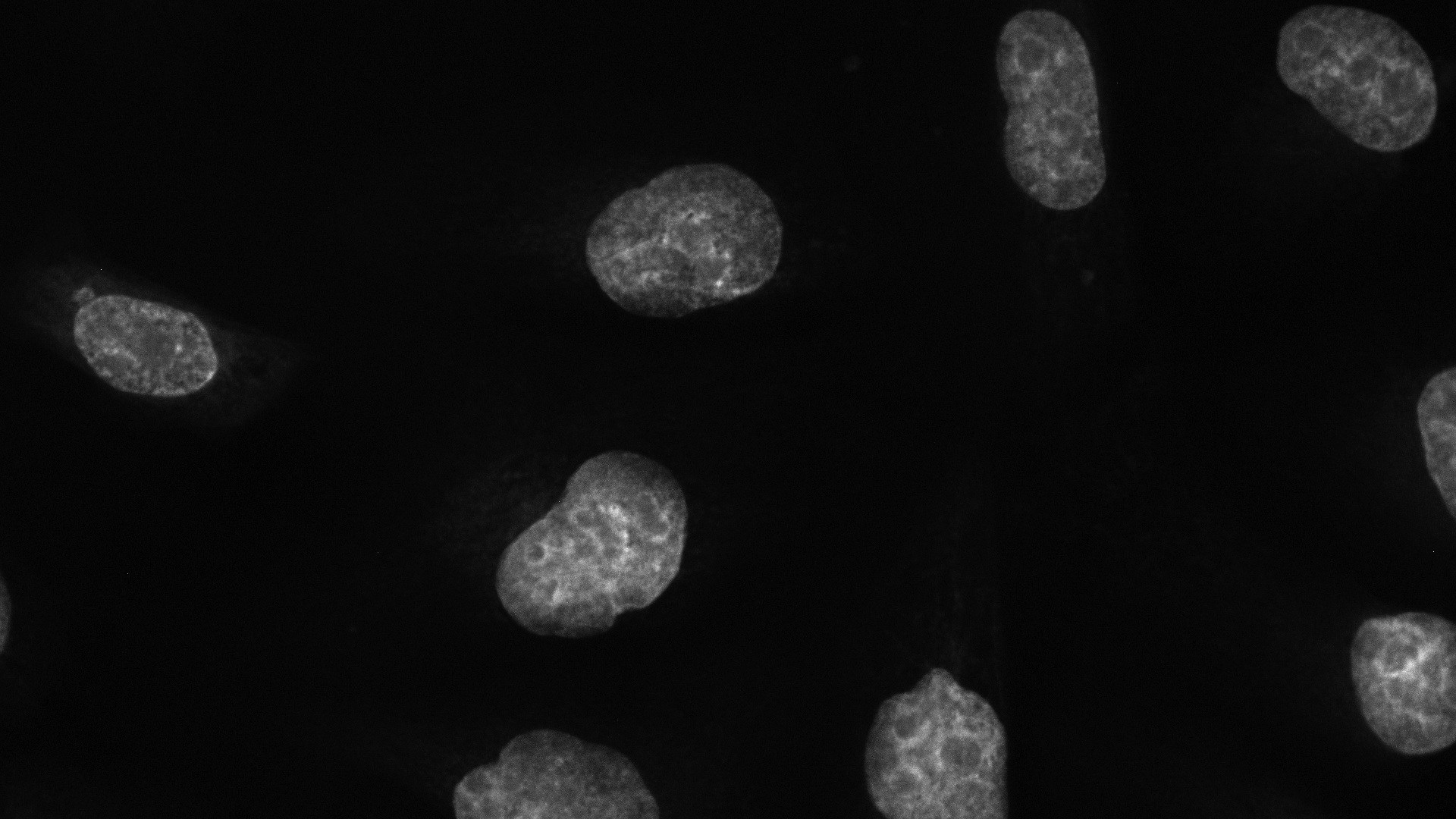

Supplement: Supplementary file 6 — Source data Fig. 5 [file 44319_2025_374_MOESM6_ESM.zip › Figure 5/SourceData5C/U2OS_MRE11_HU_DAPI.tif]

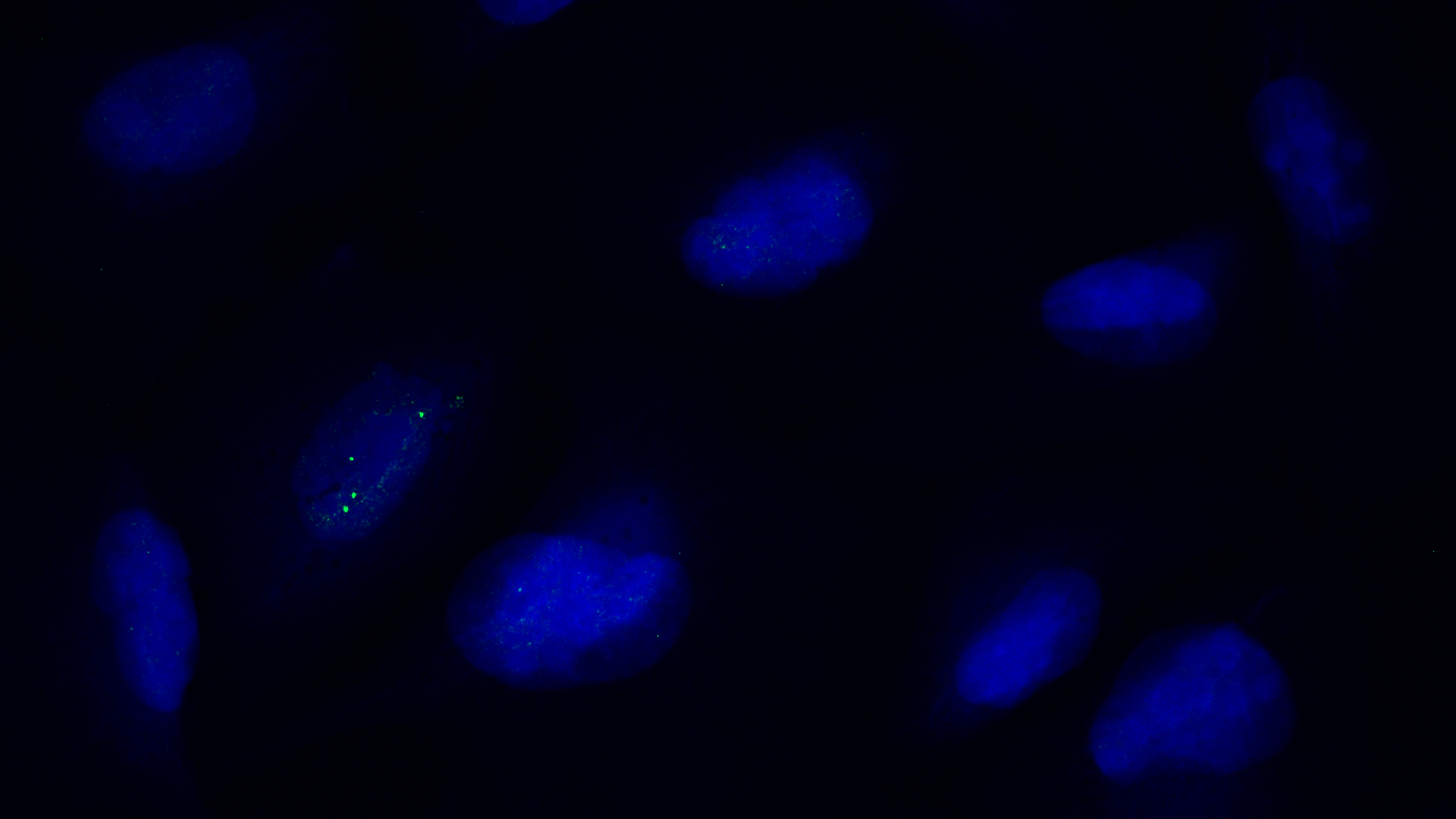

Supplement: Supplementary file 6 — Source data Fig. 5 [file 44319_2025_374_MOESM6_ESM.zip › Figure 5/SourceData5C/54L2KO1_MRE11_HU_merge.tif]

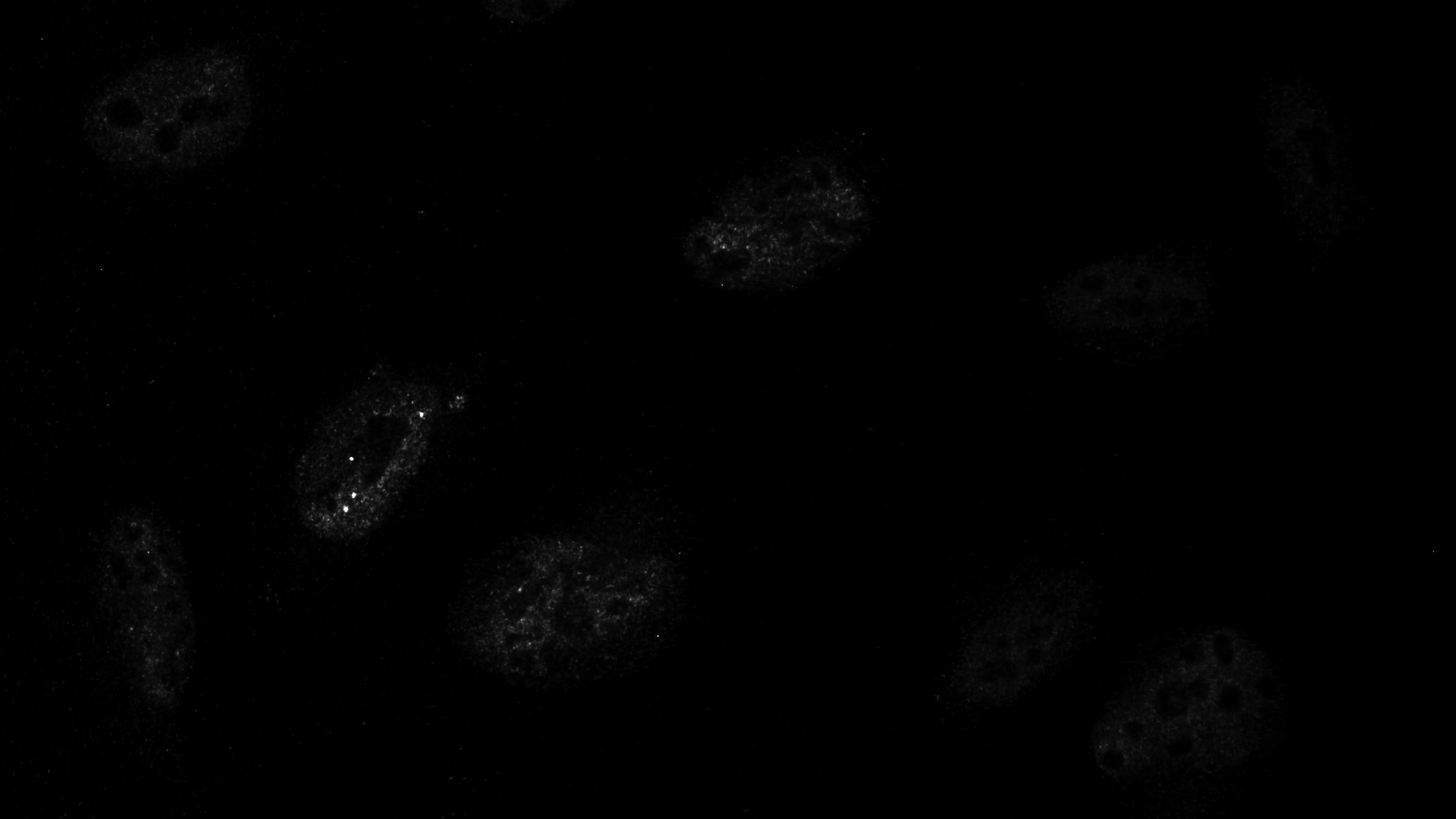

Supplement: Supplementary file 6 — Source data Fig. 5 [file 44319_2025_374_MOESM6_ESM.zip › Figure 5/SourceData5C/54L2KO1_MRE11_HU_Alexa546.tif]

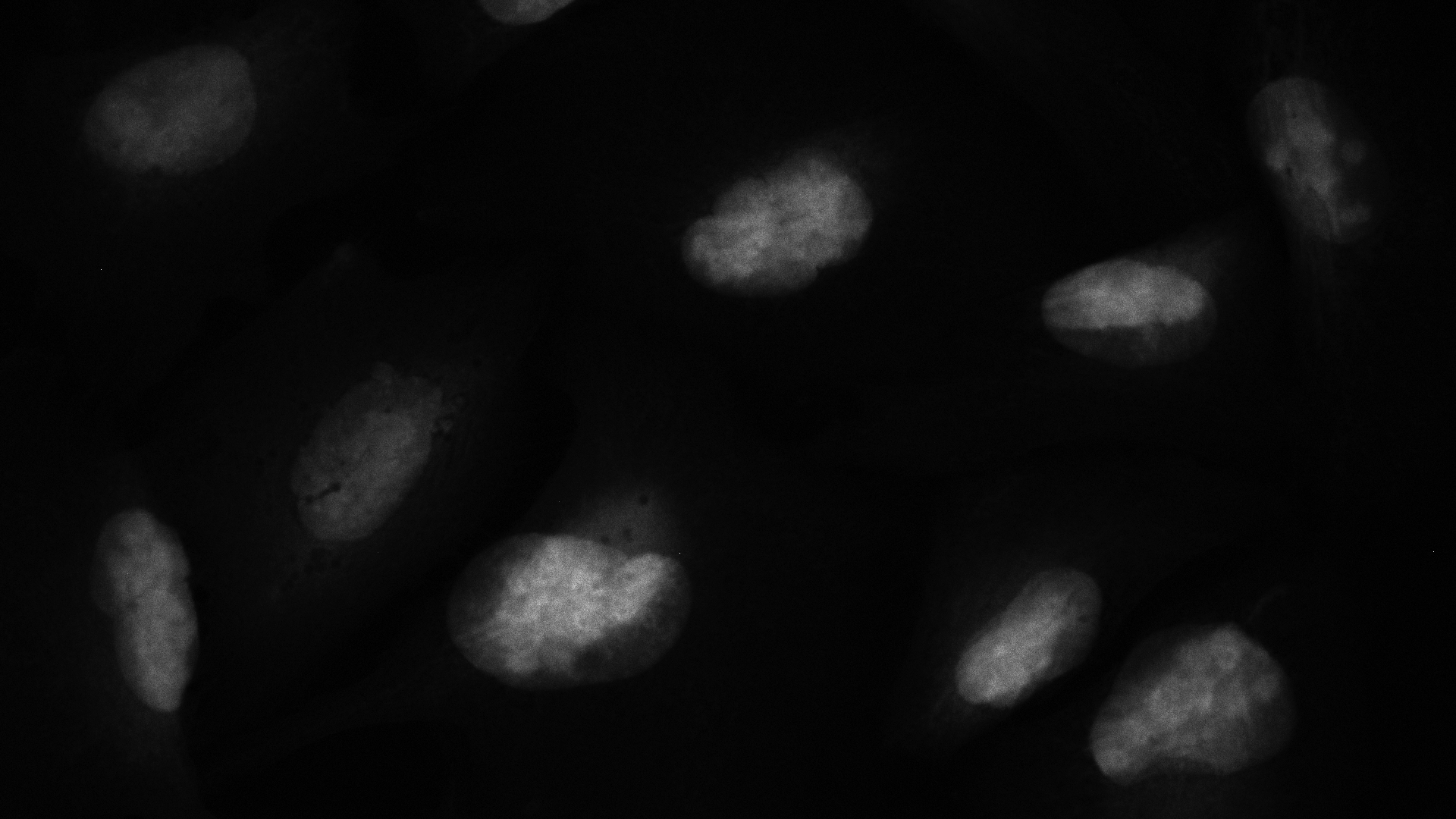

Supplement: Supplementary file 6 — Source data Fig. 5 [file 44319_2025_374_MOESM6_ESM.zip › Figure 5/SourceData5C/54L2KO1_MRE11_HU_DAPI.tif]

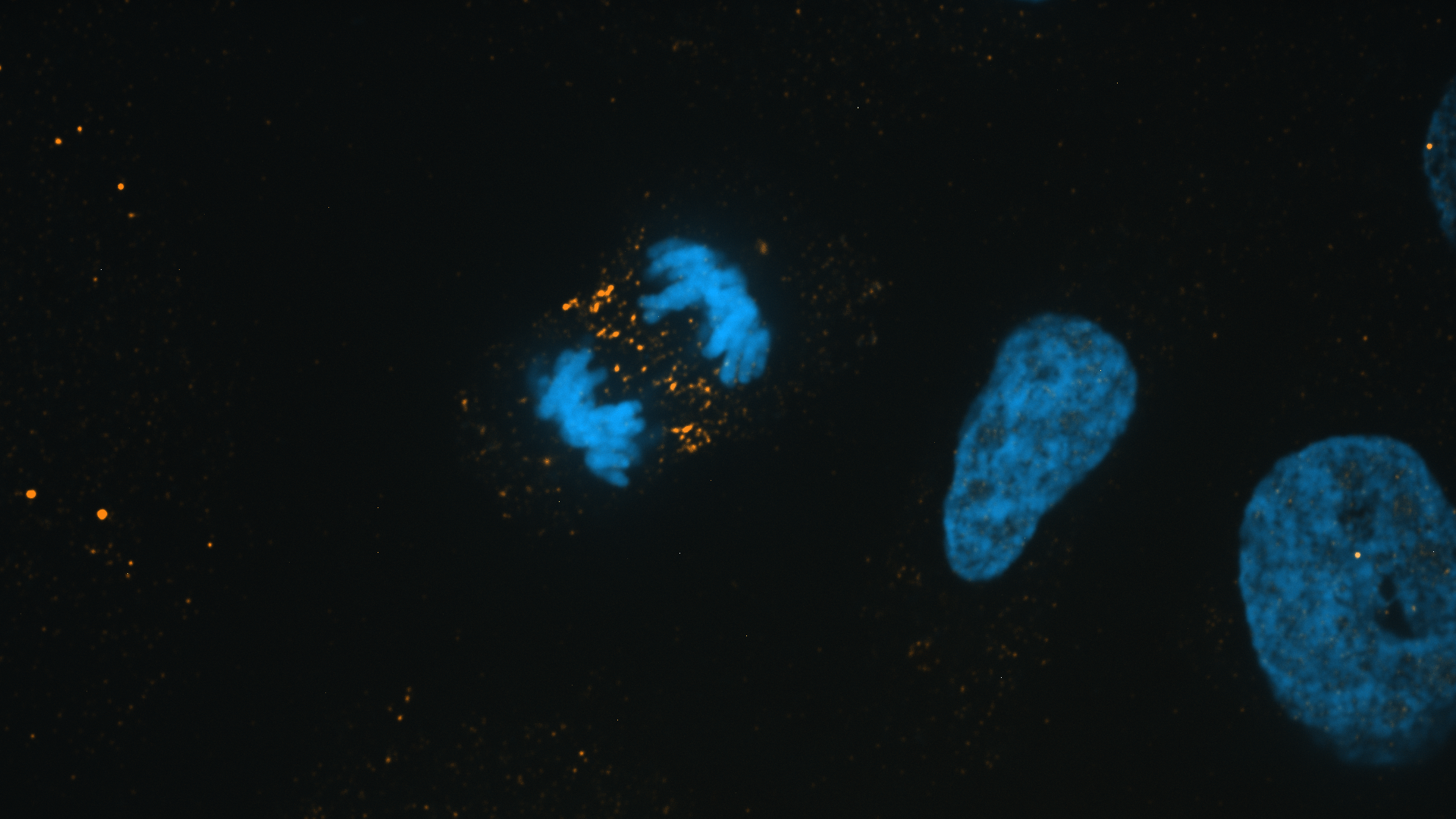

Supplement: Supplementary file 7 — Source data Fig. 6 [file 44319_2025_374_MOESM7_ESM.zip › Figure 6/SourceData6C/SourceData6C_RAD54L2KO.tif]

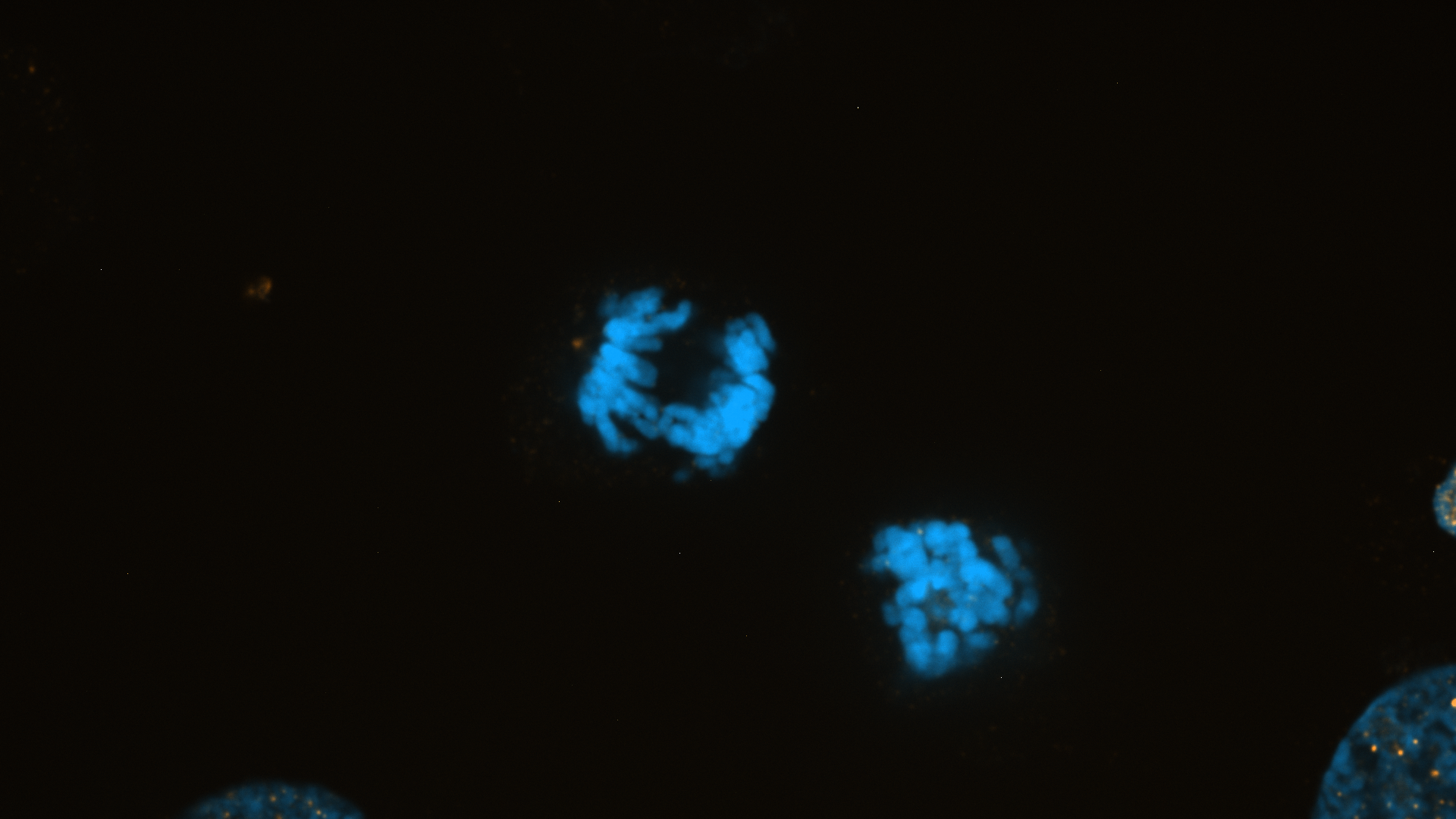

Supplement: Supplementary file 7 — Source data Fig. 6 [file 44319_2025_374_MOESM7_ESM.zip › Figure 6/SourceData6C/SourceData6C_WT.tif]

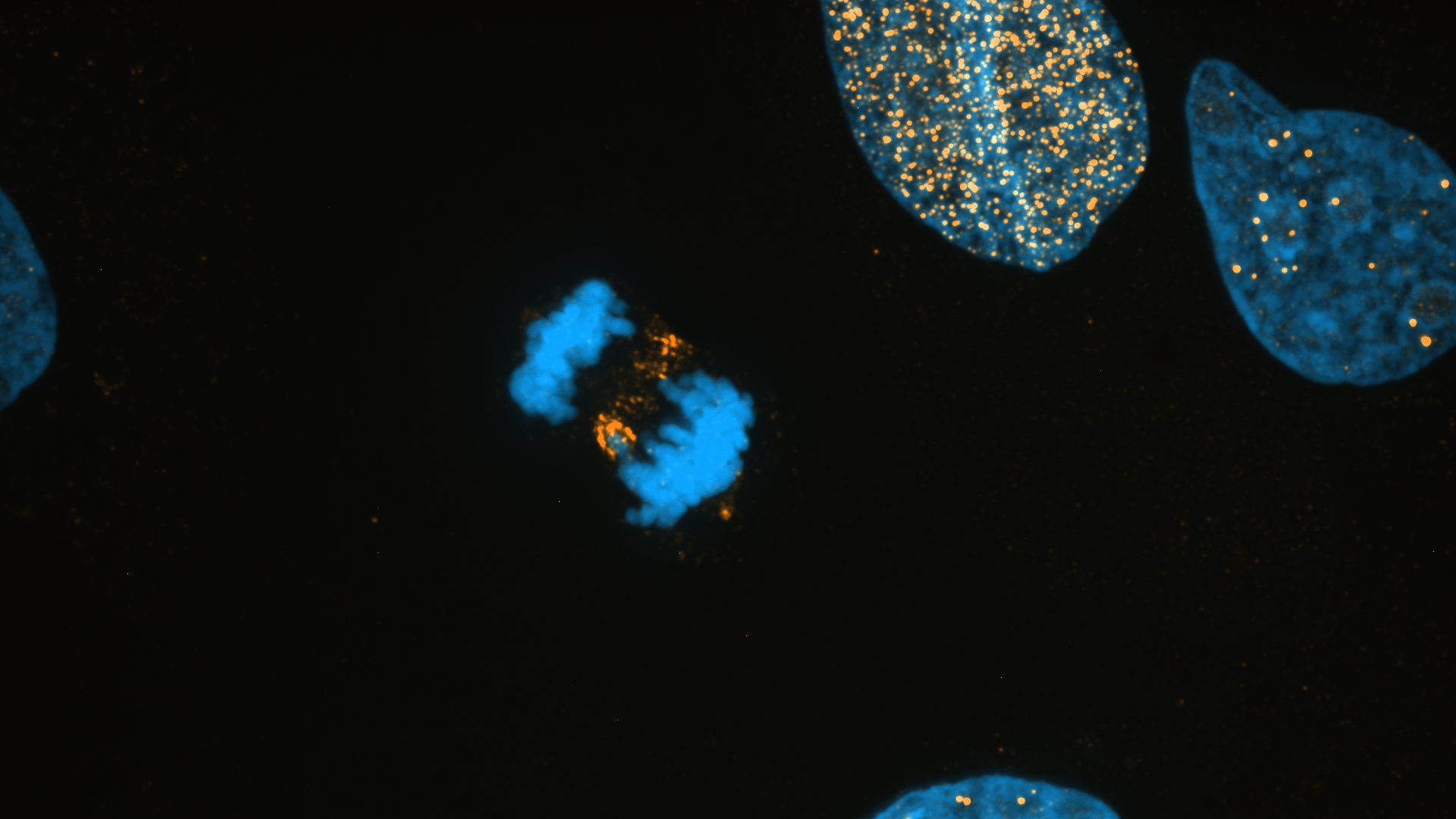

Supplement: Supplementary file 7 — Source data Fig. 6 [file 44319_2025_374_MOESM7_ESM.zip › Figure 6/SourceData6C/SourceData6C_siBLM.tif]

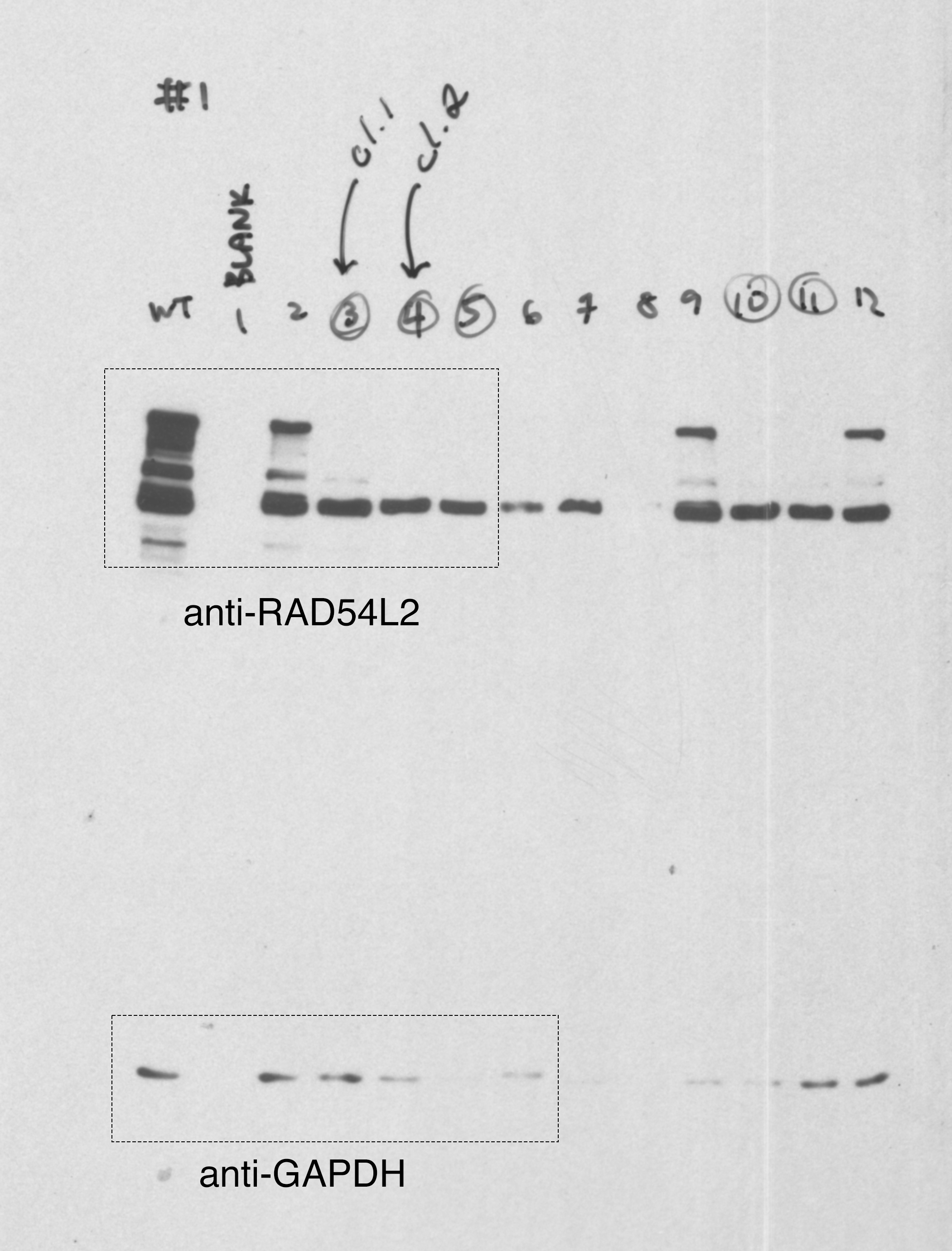

Supplement: Supplementary file 8 — EV Figure Source Data [file 44319_2025_374_MOESM8_ESM.zip › Source_Data_EV_Figures/Figure EV4/SourceDataEV4A/SourceDataForFigureEV4A.tif]

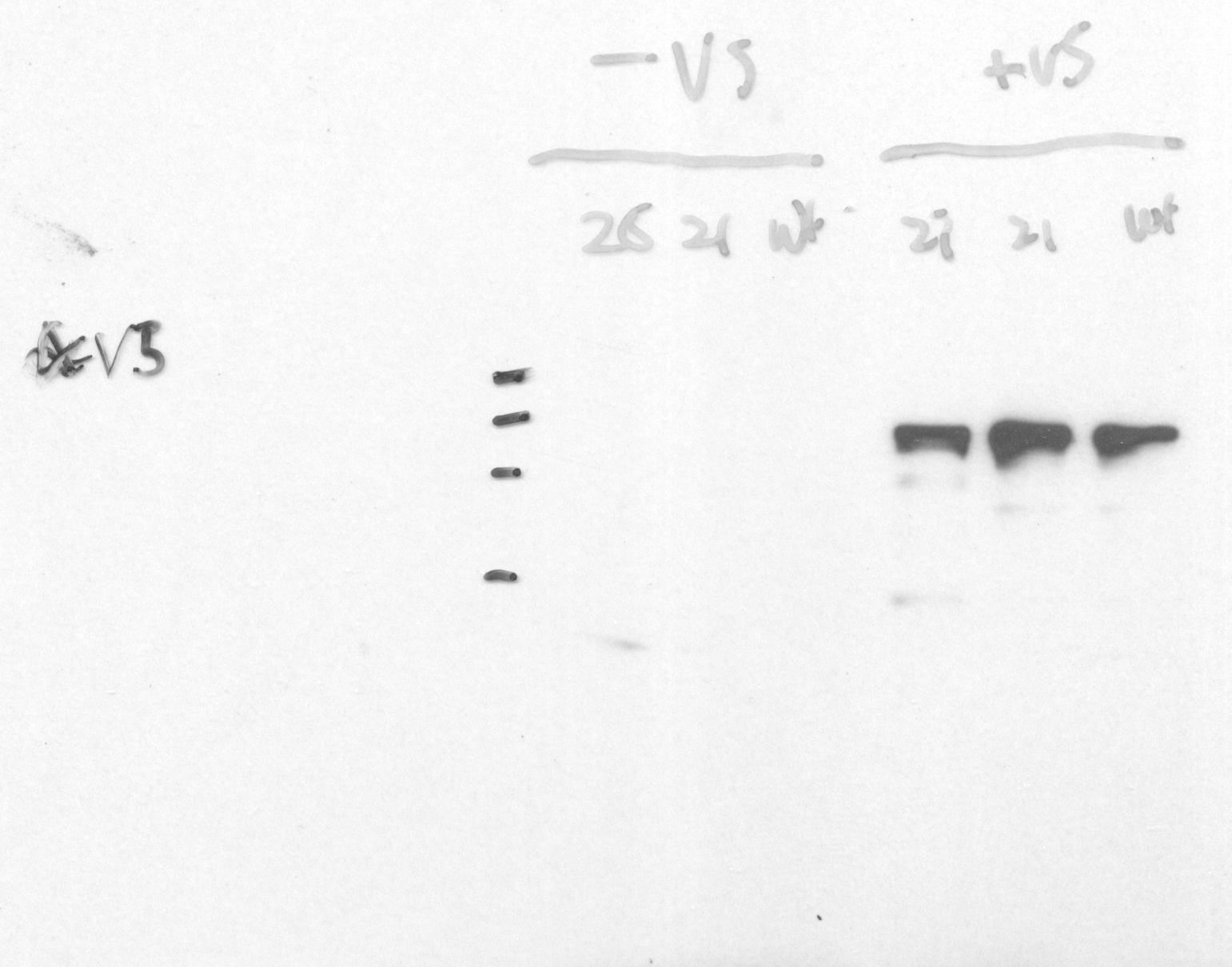

Supplement: Supplementary file 8 — EV Figure Source Data [file 44319_2025_374_MOESM8_ESM.zip › Source_Data_EV_Figures/Figure EV4/SourceDataEV4D/SourceDataForFigureEV4D_V5.tif]

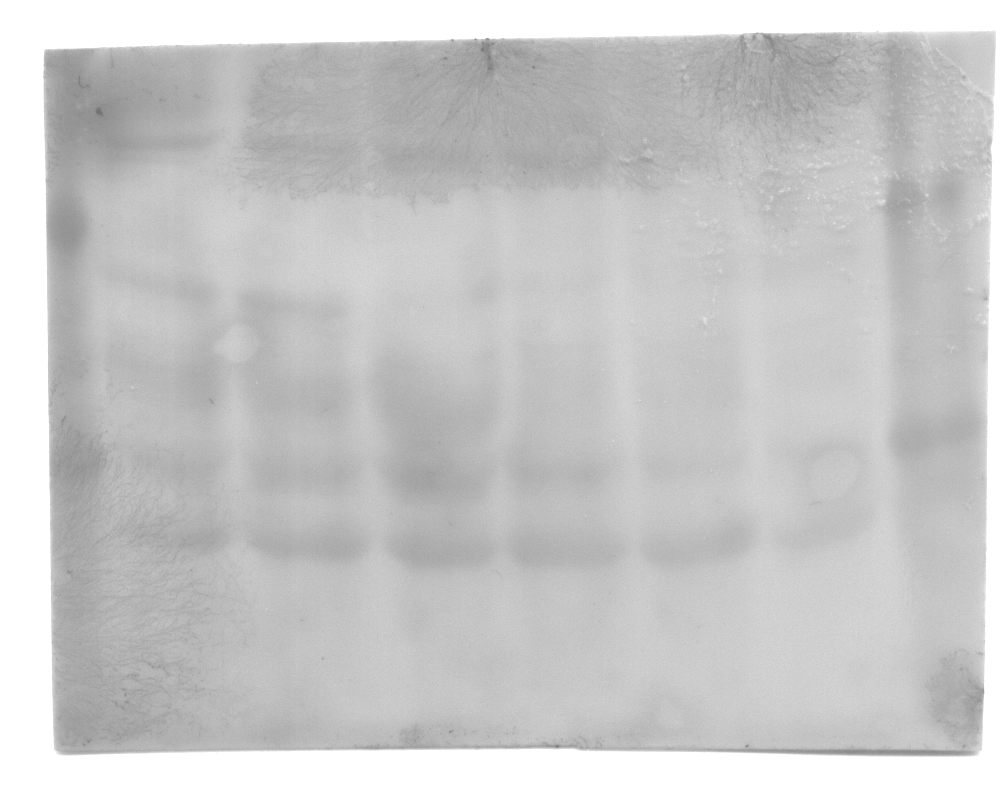

Supplement: Supplementary file 8 — EV Figure Source Data [file 44319_2025_374_MOESM8_ESM.zip › Source_Data_EV_Figures/Figure EV4/SourceDataEV4D/SourceDataForFigureEV4D_PonceauS.tif]

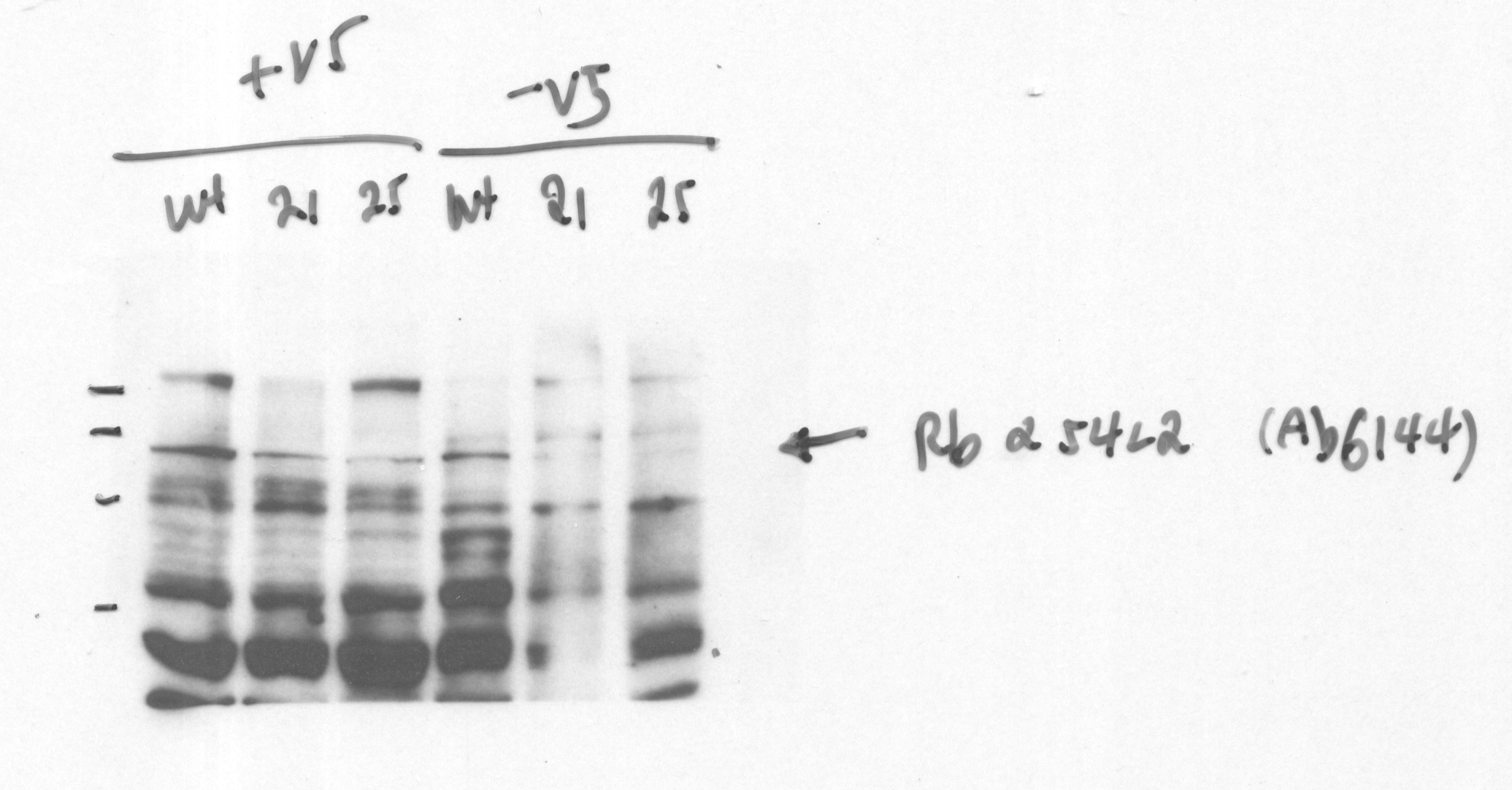

Supplement: Supplementary file 8 — EV Figure Source Data [file 44319_2025_374_MOESM8_ESM.zip › Source_Data_EV_Figures/Figure EV4/SourceDataEV4D/SourceDataForFigureEV4D_RAD54L2.tif]

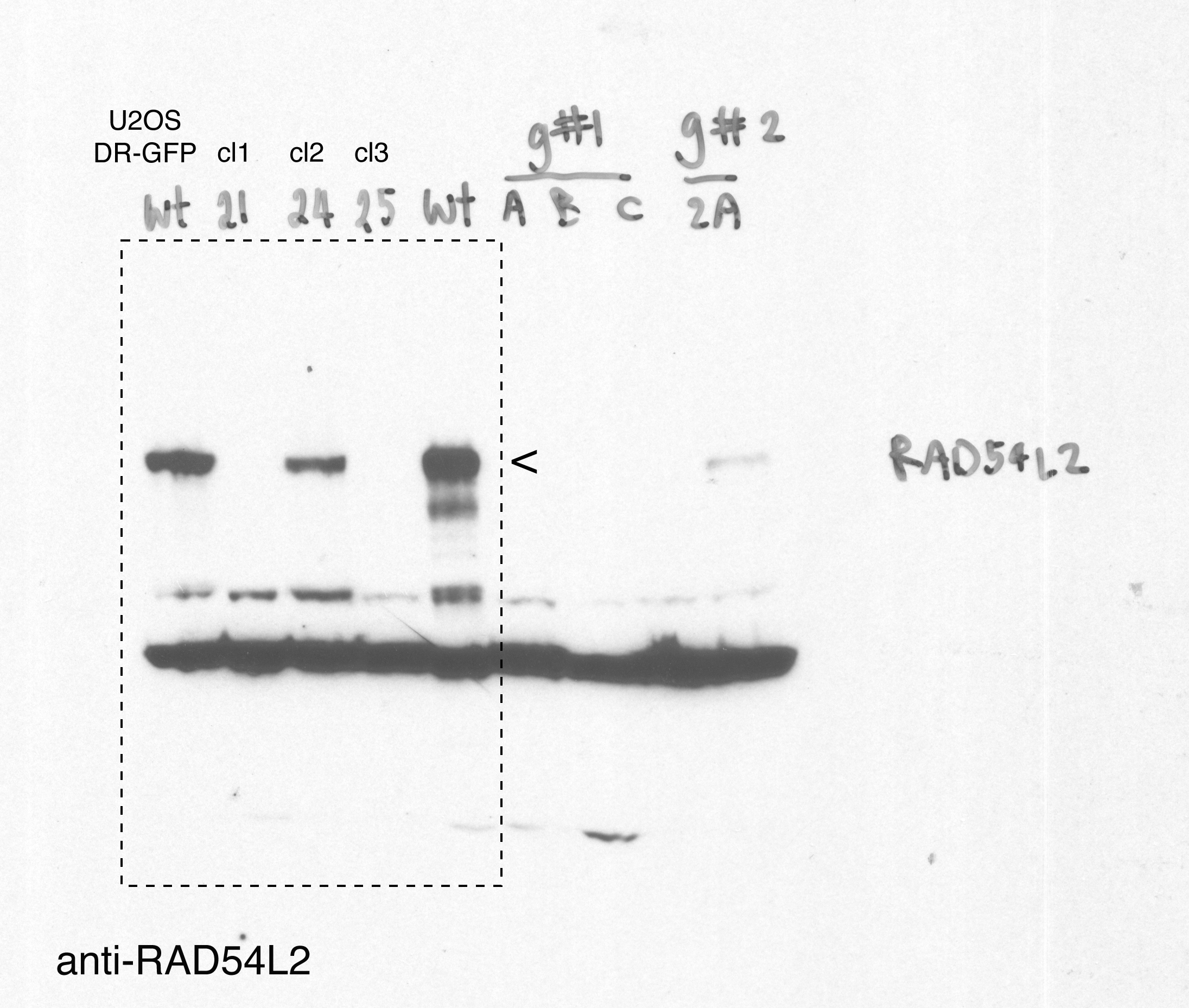

Supplement: Supplementary file 8 — EV Figure Source Data [file 44319_2025_374_MOESM8_ESM.zip › Source_Data_EV_Figures/Figure EV4/SourceDataEV4C/SourceDataForFigureEV4C_54L2.tif]

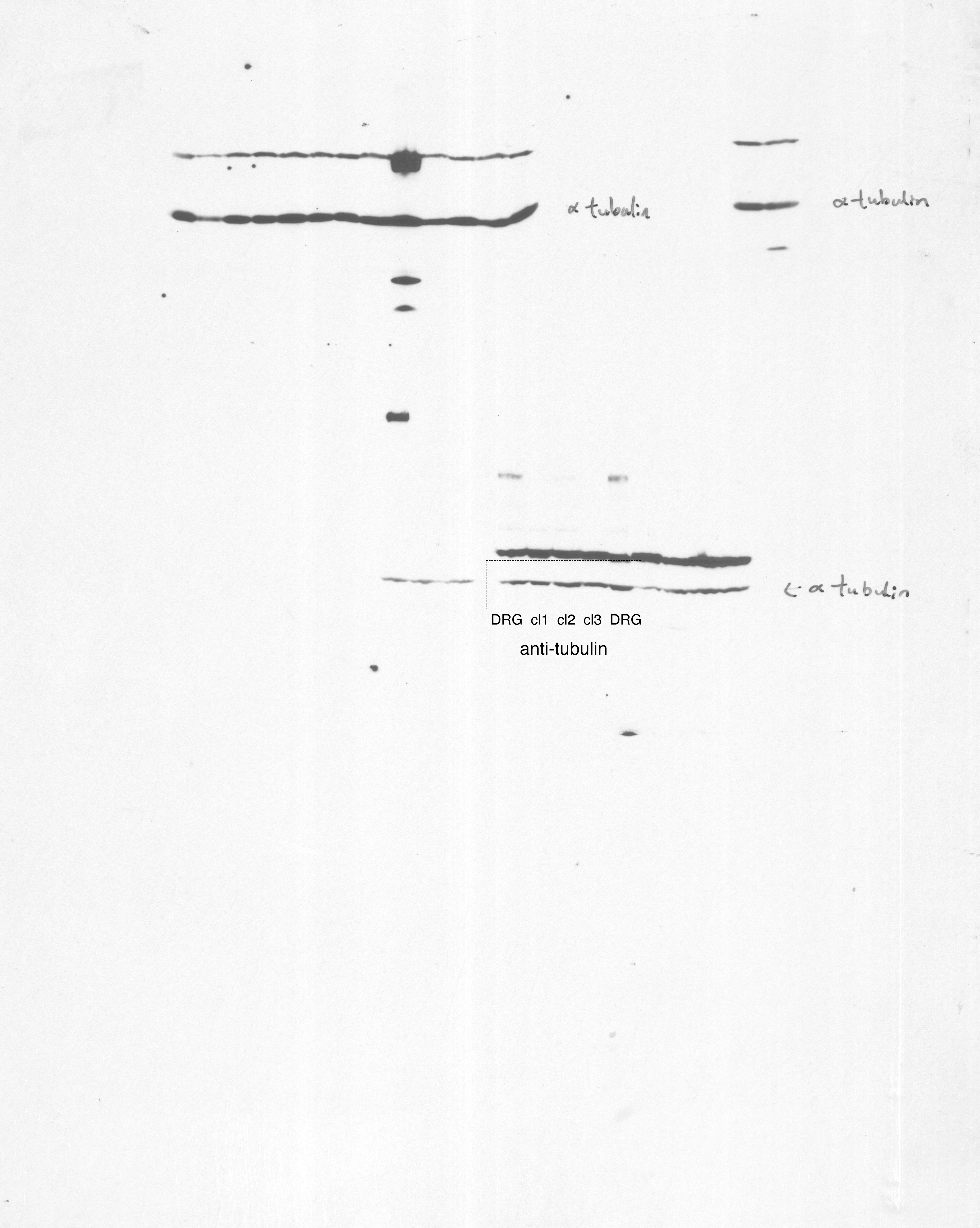

Supplement: Supplementary file 8 — EV Figure Source Data [file 44319_2025_374_MOESM8_ESM.zip › Source_Data_EV_Figures/Figure EV4/SourceDataEV4C/SourceDataForFigureEV4C_tubulin.tif]

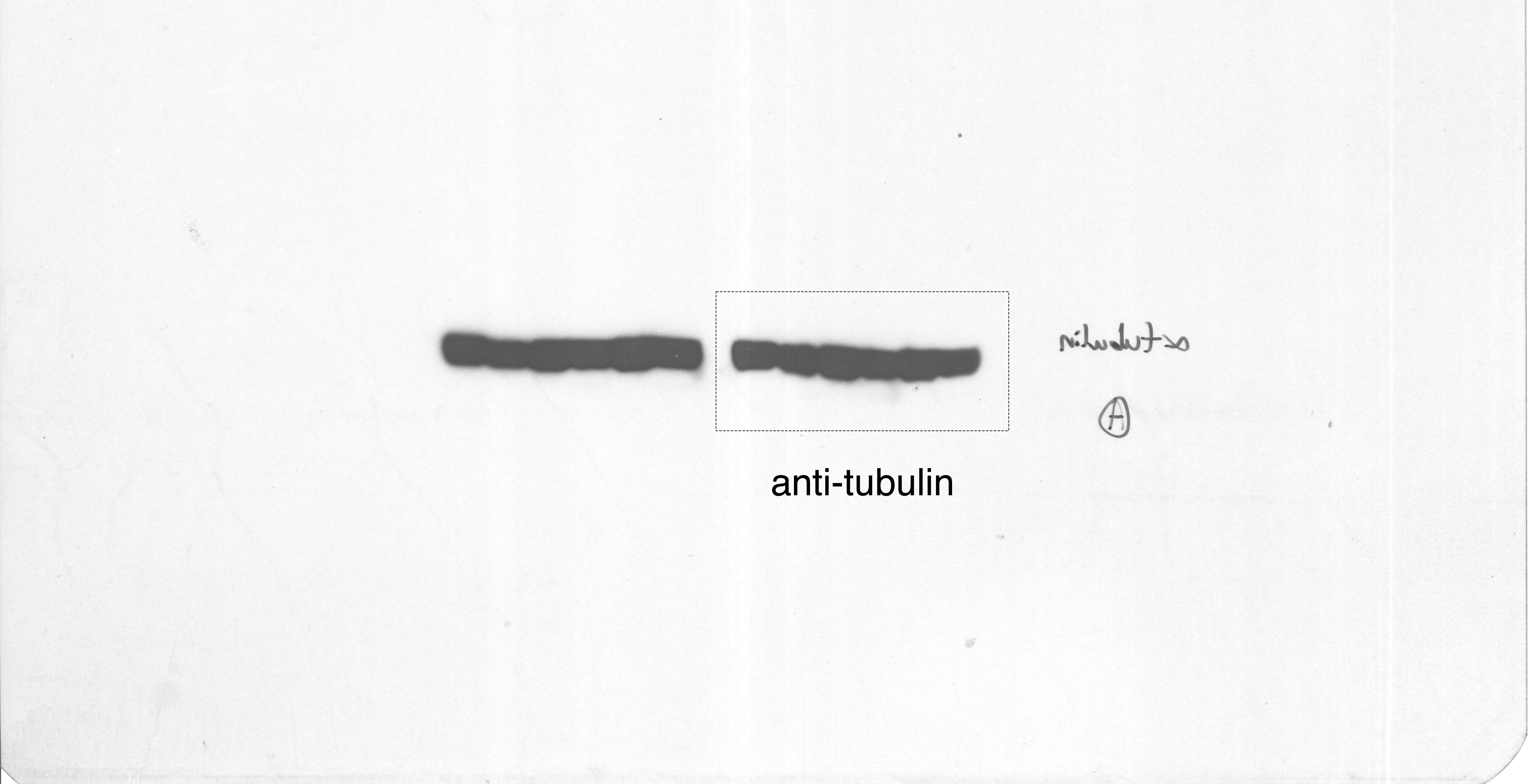

Supplement: Supplementary file 8 — EV Figure Source Data [file 44319_2025_374_MOESM8_ESM.zip › Source_Data_EV_Figures/Figure EV4/SourceDataEV4B/SourceDataForFigureEV4B_tubulin.tif]

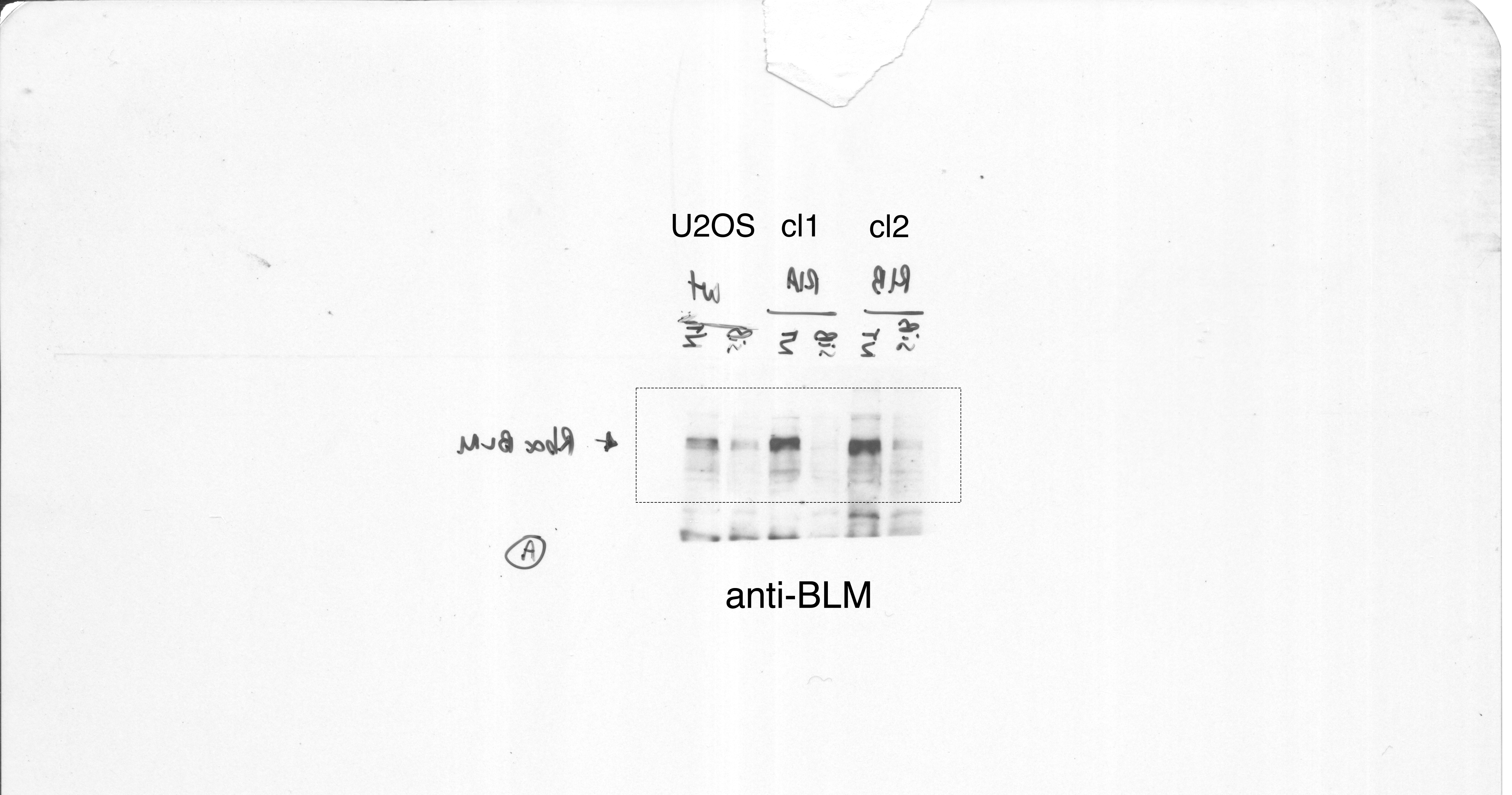

Supplement: Supplementary file 8 — EV Figure Source Data [file 44319_2025_374_MOESM8_ESM.zip › Source_Data_EV_Figures/Figure EV4/SourceDataEV4B/SourceDataForFigureEV4B_BLM.tif]

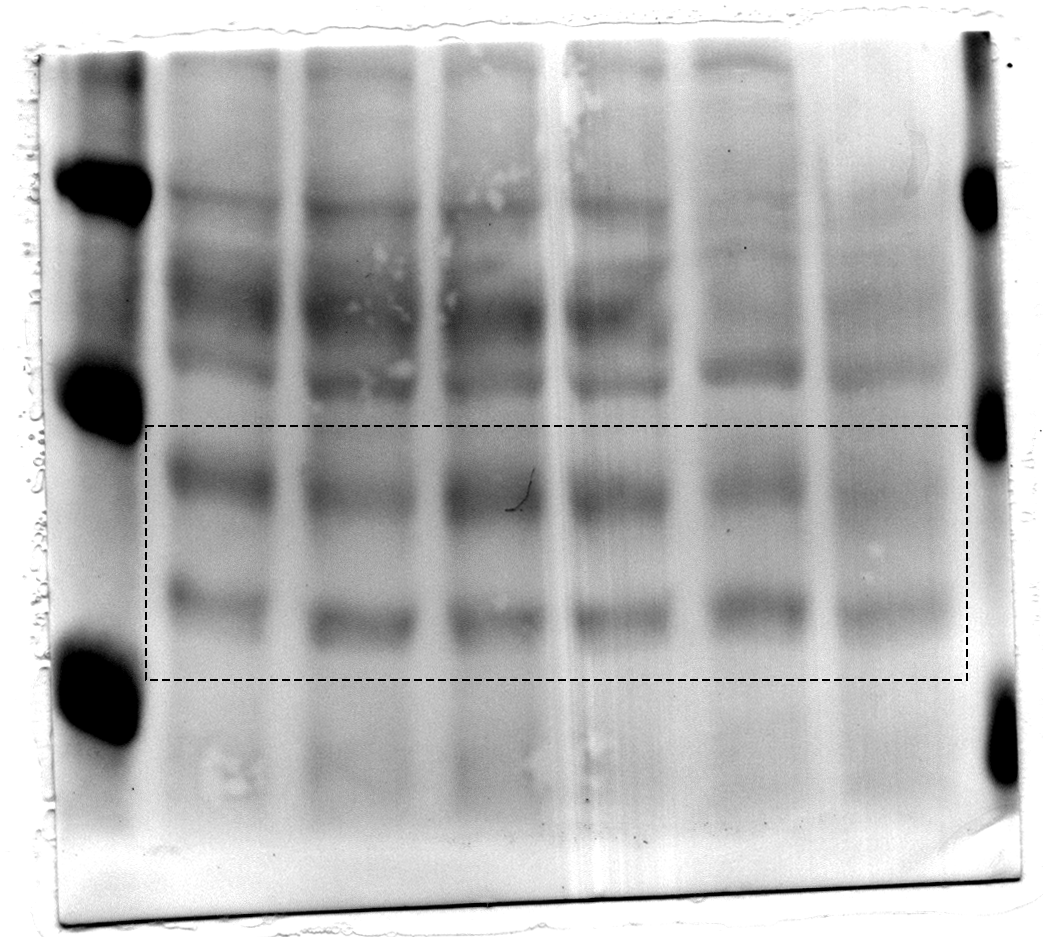

Supplement: Supplementary file 8 — EV Figure Source Data [file 44319_2025_374_MOESM8_ESM.zip › Source_Data_EV_Figures/Figure EV4/SourceDataEV4B/SourceDataForFigureEV4B_ponceauS.tif]

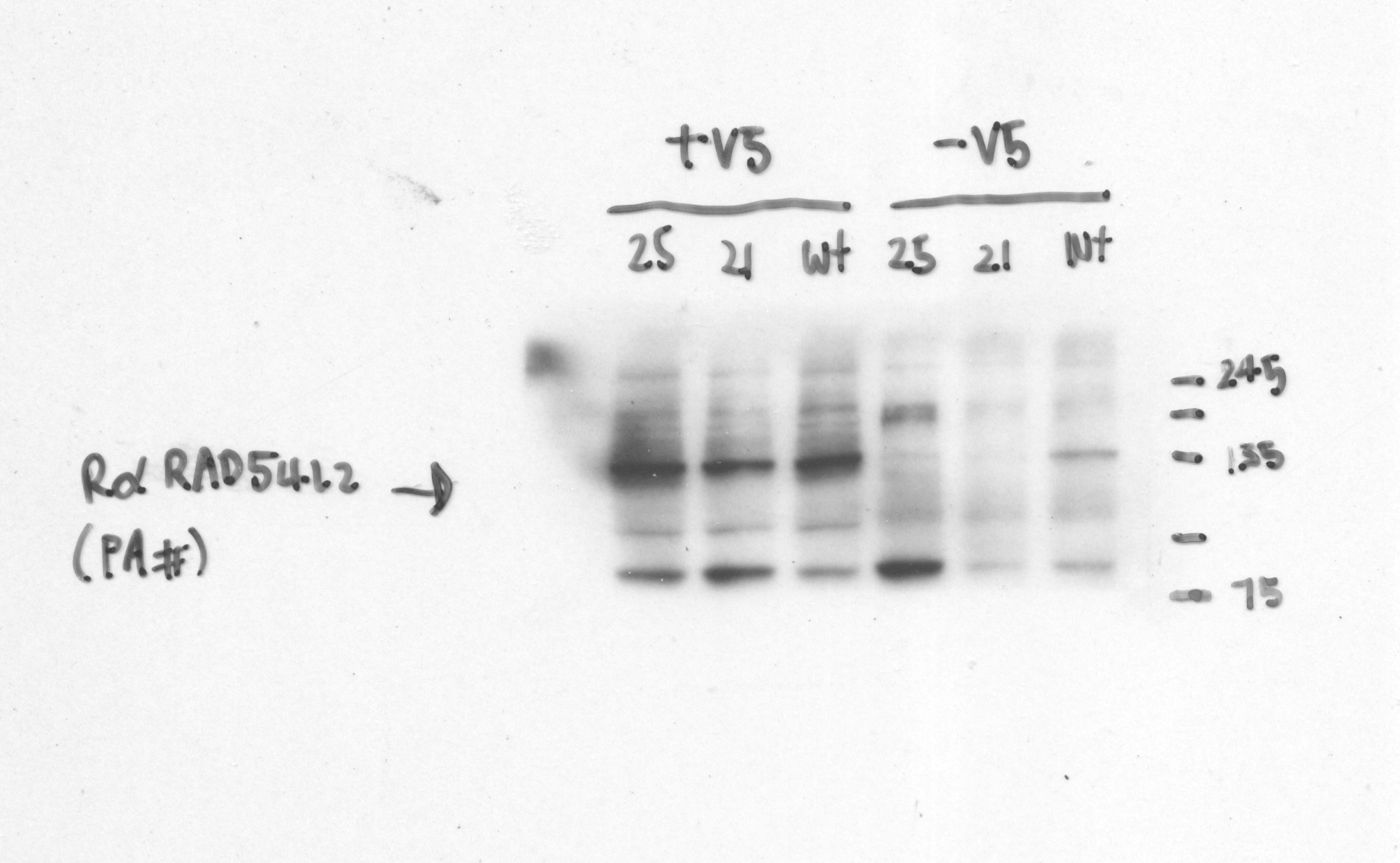

Supplement: Supplementary file 8 — EV Figure Source Data [file 44319_2025_374_MOESM8_ESM.zip › Source_Data_EV_Figures/Figure EV4/SourceDataEV4E/SourceDataForFigureEV4E_RAD54L2.tif]

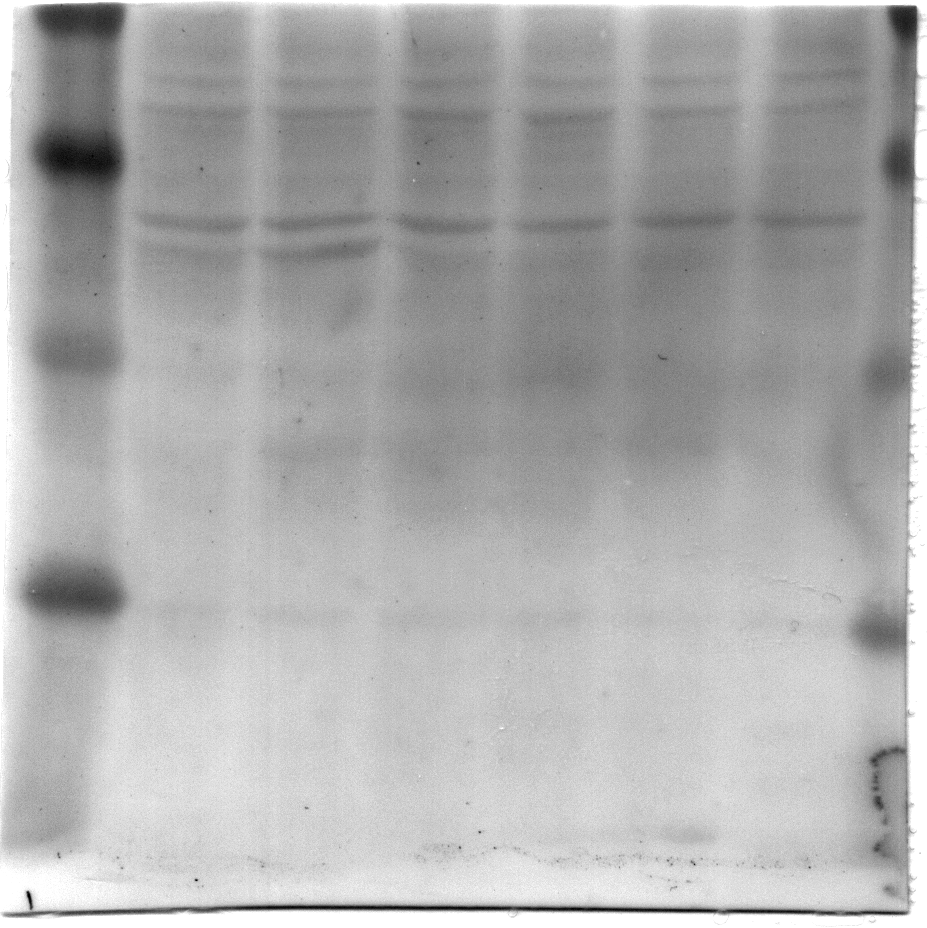

Supplement: Supplementary file 8 — EV Figure Source Data [file 44319_2025_374_MOESM8_ESM.zip › Source_Data_EV_Figures/Figure EV4/SourceDataEV4E/SourceDataForFigureEV4E_PonceauS.tif]

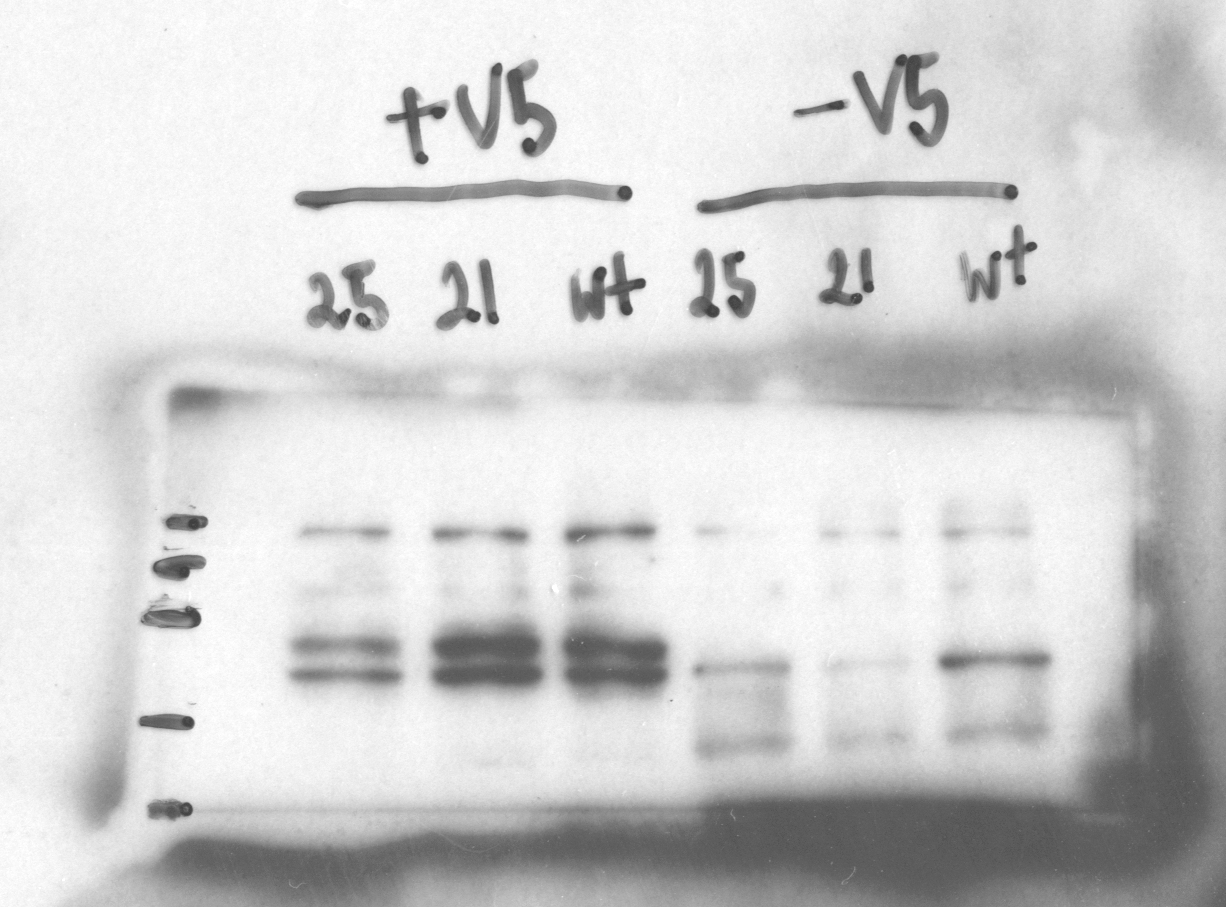

Supplement: Supplementary file 8 — EV Figure Source Data [file 44319_2025_374_MOESM8_ESM.zip › Source_Data_EV_Figures/Figure EV4/SourceDataEV4E/SourceDataForFigureEV4E_V5.tif]

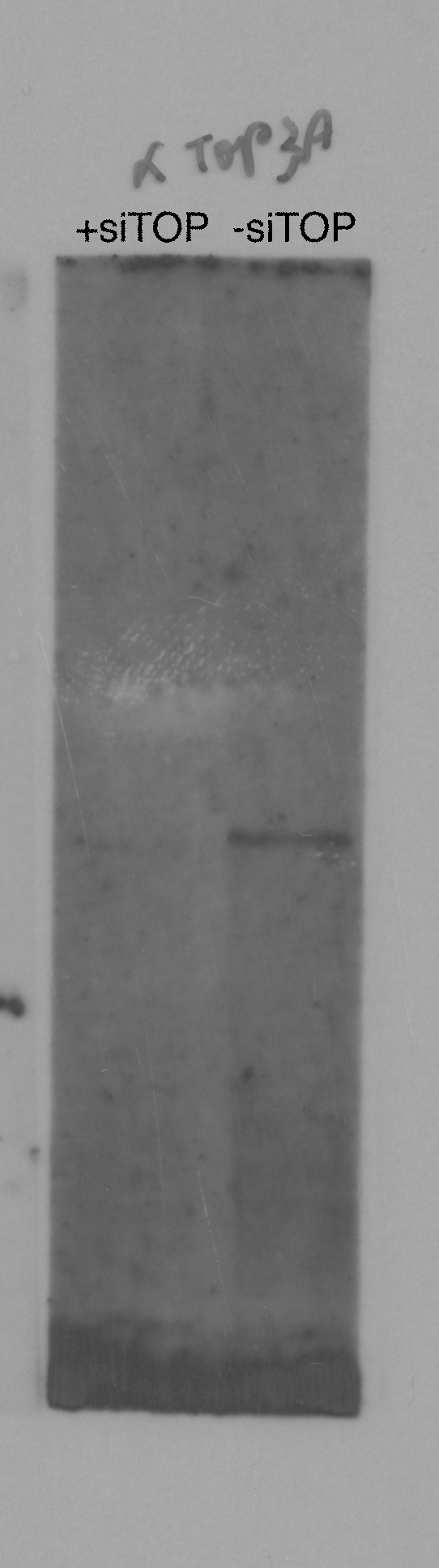

Supplement: Supplementary file 8 — EV Figure Source Data [file 44319_2025_374_MOESM8_ESM.zip › Source_Data_EV_Figures/Figure EV3/SourceDataEV3A/SourceDataForFigureEV3A.TOP3A.tif]

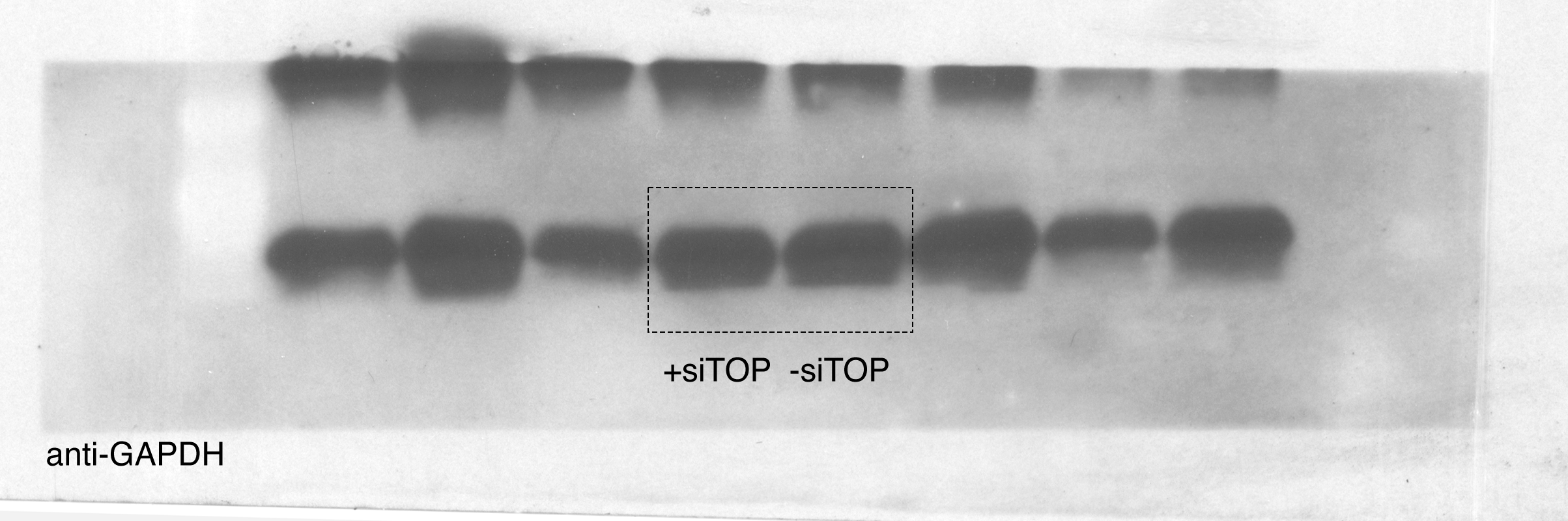

Supplement: Supplementary file 8 — EV Figure Source Data [file 44319_2025_374_MOESM8_ESM.zip › Source_Data_EV_Figures/Figure EV3/SourceDataEV3A/SourceDataForFigureEV3A.GAPDH.tif]

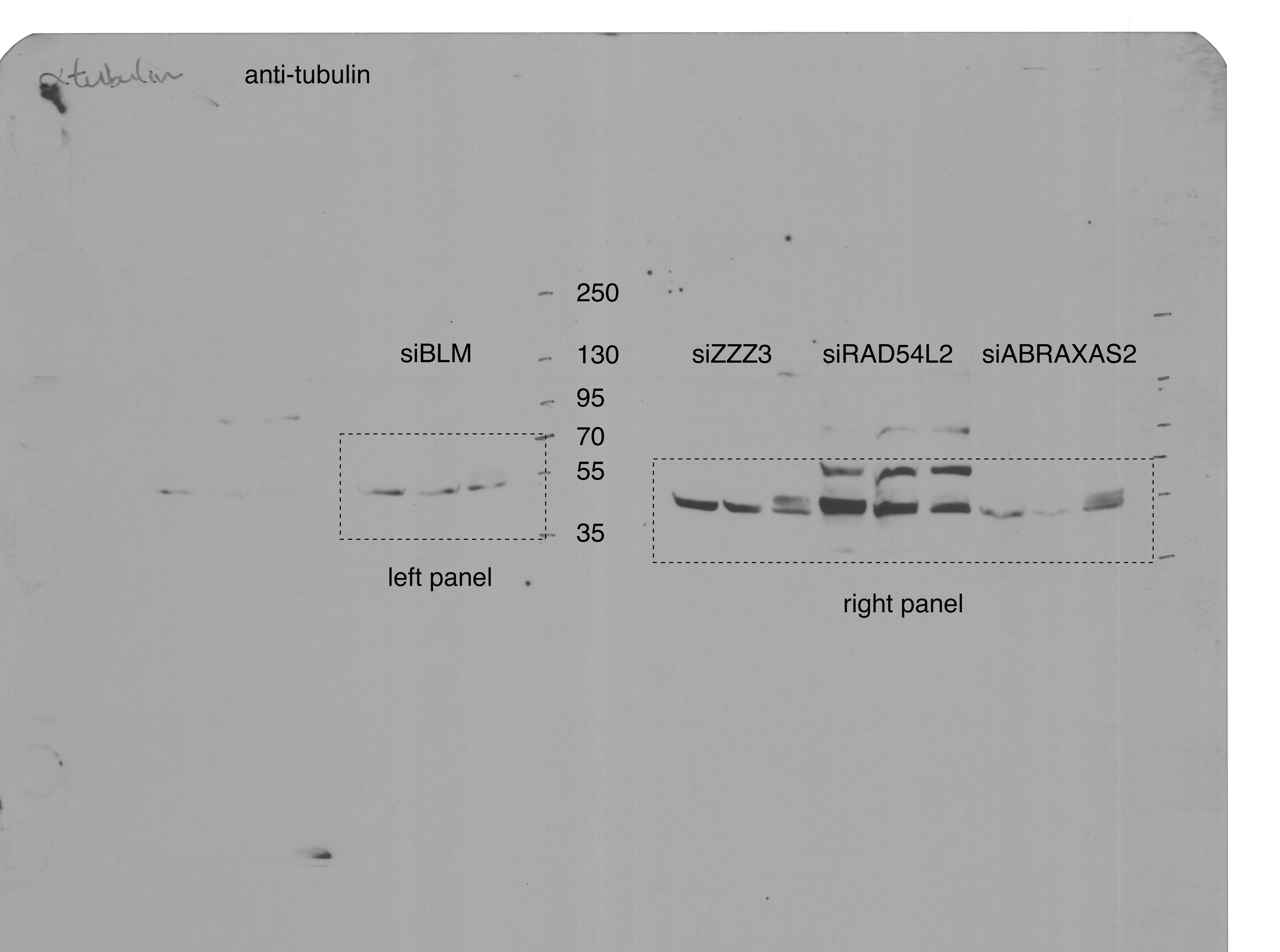

Supplement: Supplementary file 8 — EV Figure Source Data [file 44319_2025_374_MOESM8_ESM.zip › Source_Data_EV_Figures/Figure EV3/SourceDataEV3C/SourceDataForFigureEV3C.TUB.tif]

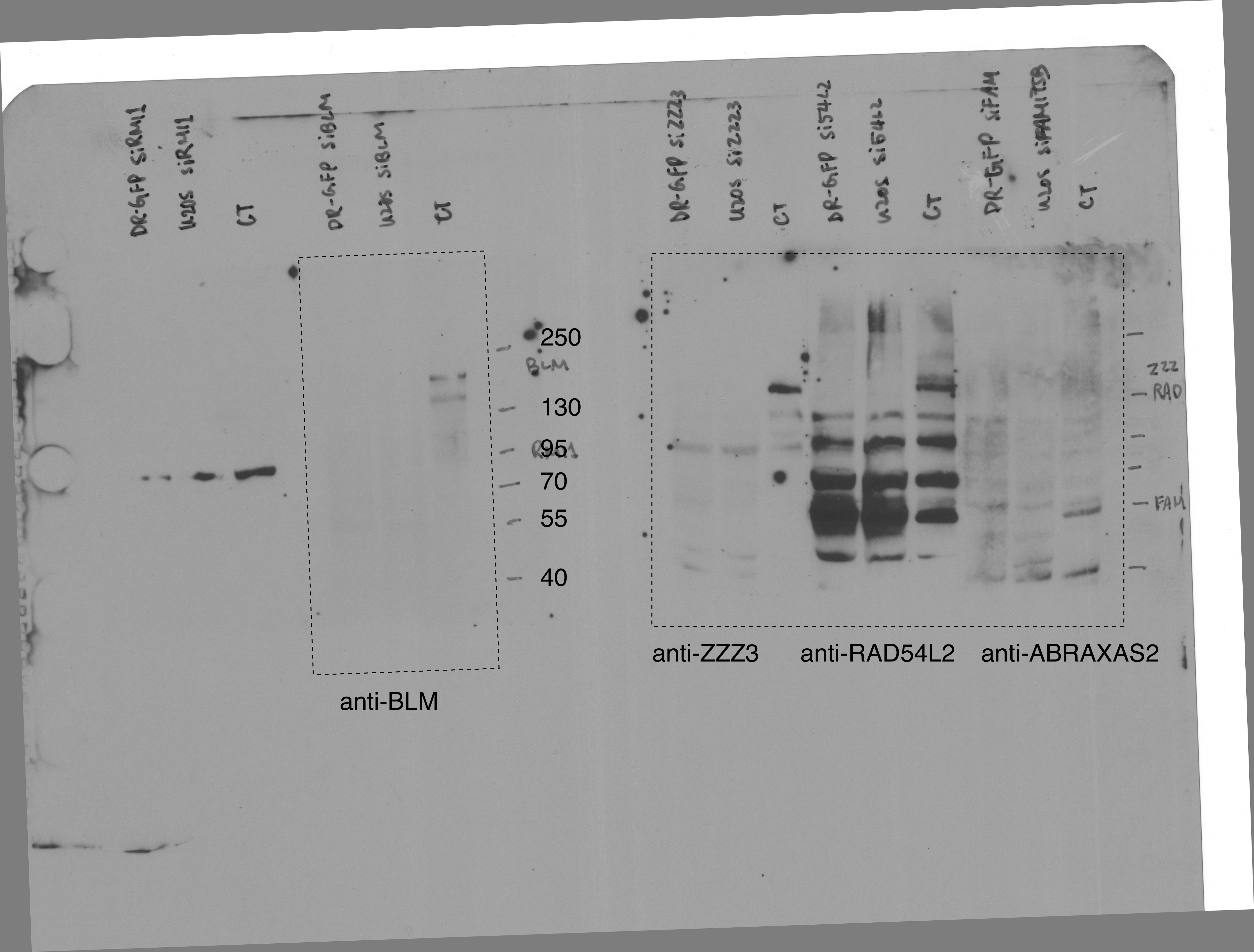

Supplement: Supplementary file 8 — EV Figure Source Data [file 44319_2025_374_MOESM8_ESM.zip › Source_Data_EV_Figures/Figure EV3/SourceDataEV3C/SourceDataForFigureEV3C.si.tif]

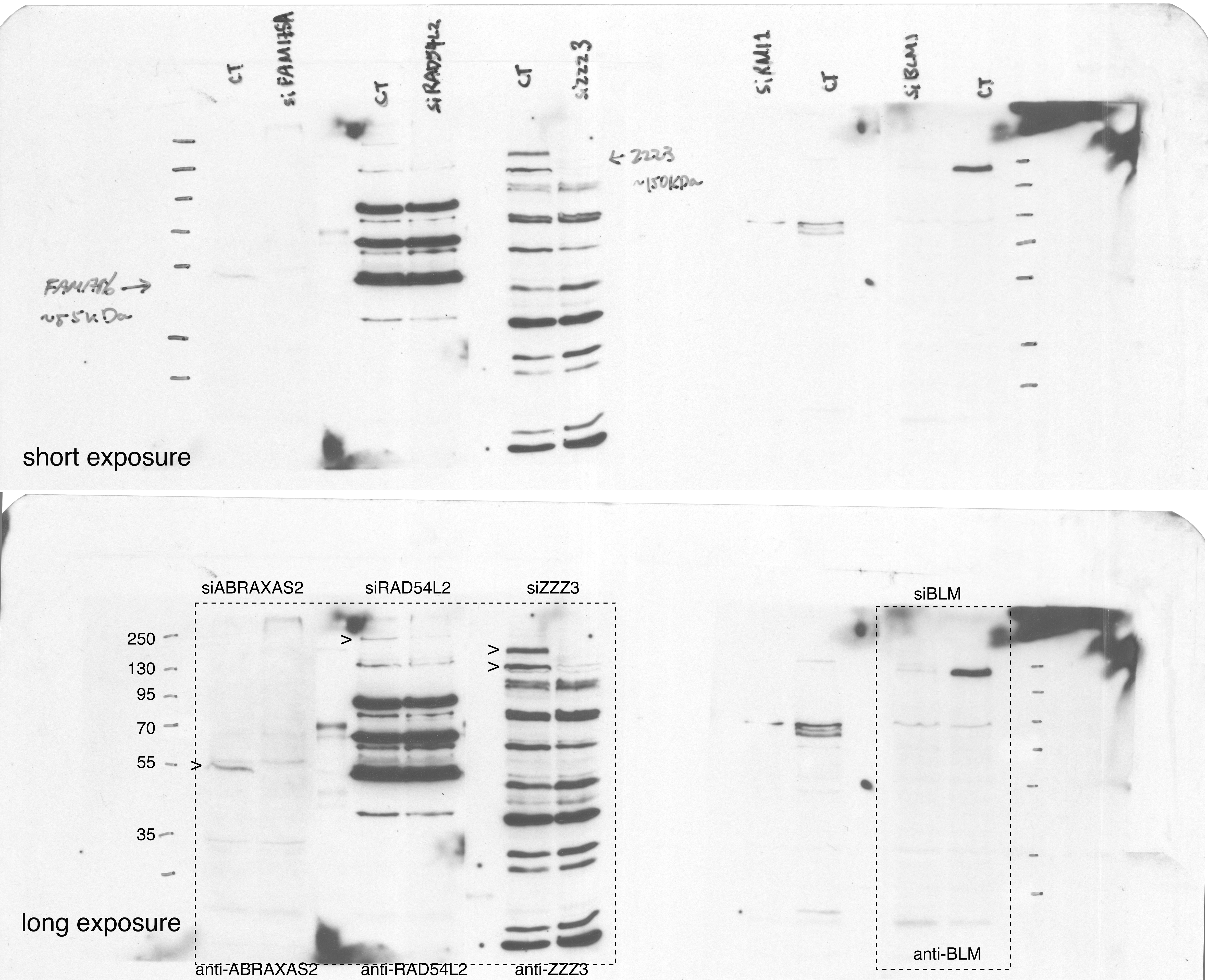

Supplement: Supplementary file 8 — EV Figure Source Data [file 44319_2025_374_MOESM8_ESM.zip › Source_Data_EV_Figures/Figure EV3/SourceDataEV3B/SourceDataForFigureEV3B.si.tif]

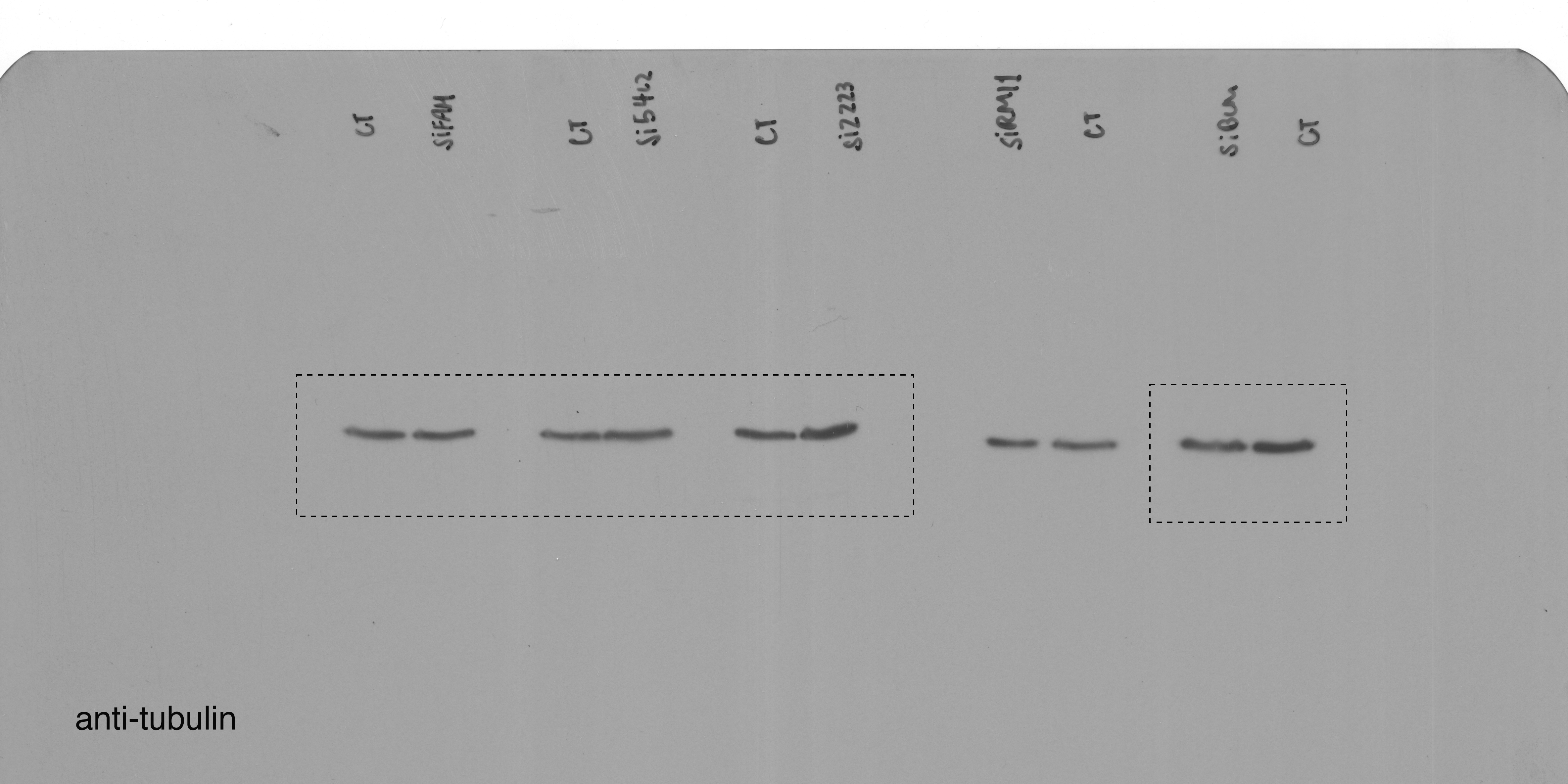

Supplement: Supplementary file 8 — EV Figure Source Data [file 44319_2025_374_MOESM8_ESM.zip › Source_Data_EV_Figures/Figure EV3/SourceDataEV3B/SourceDataForFigureEV3B.TUB.tif]

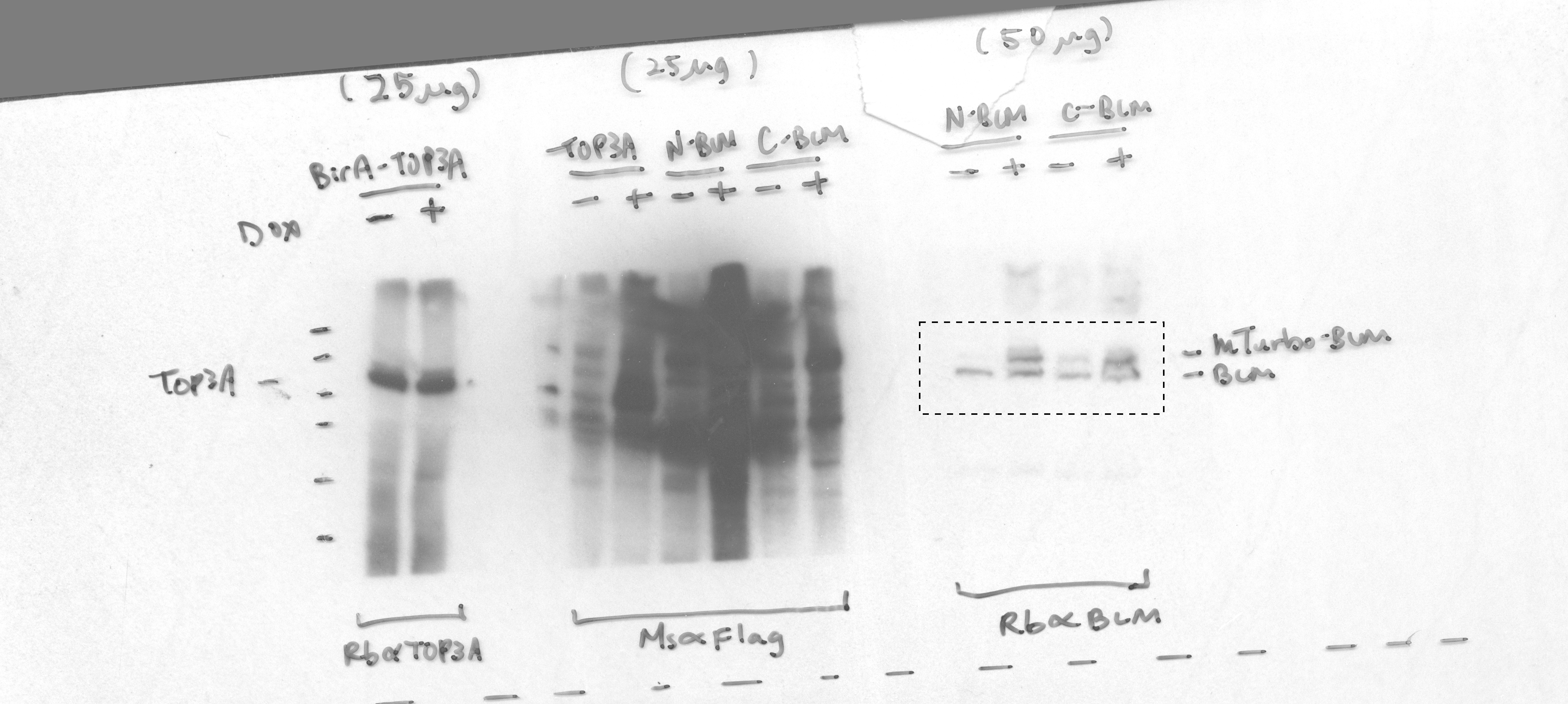

Supplement: Supplementary file 8 — EV Figure Source Data [file 44319_2025_374_MOESM8_ESM.zip › Source_Data_EV_Figures/Figure EV2/SourceDataEV2B/SourceDataForFigureEV2B.tif]

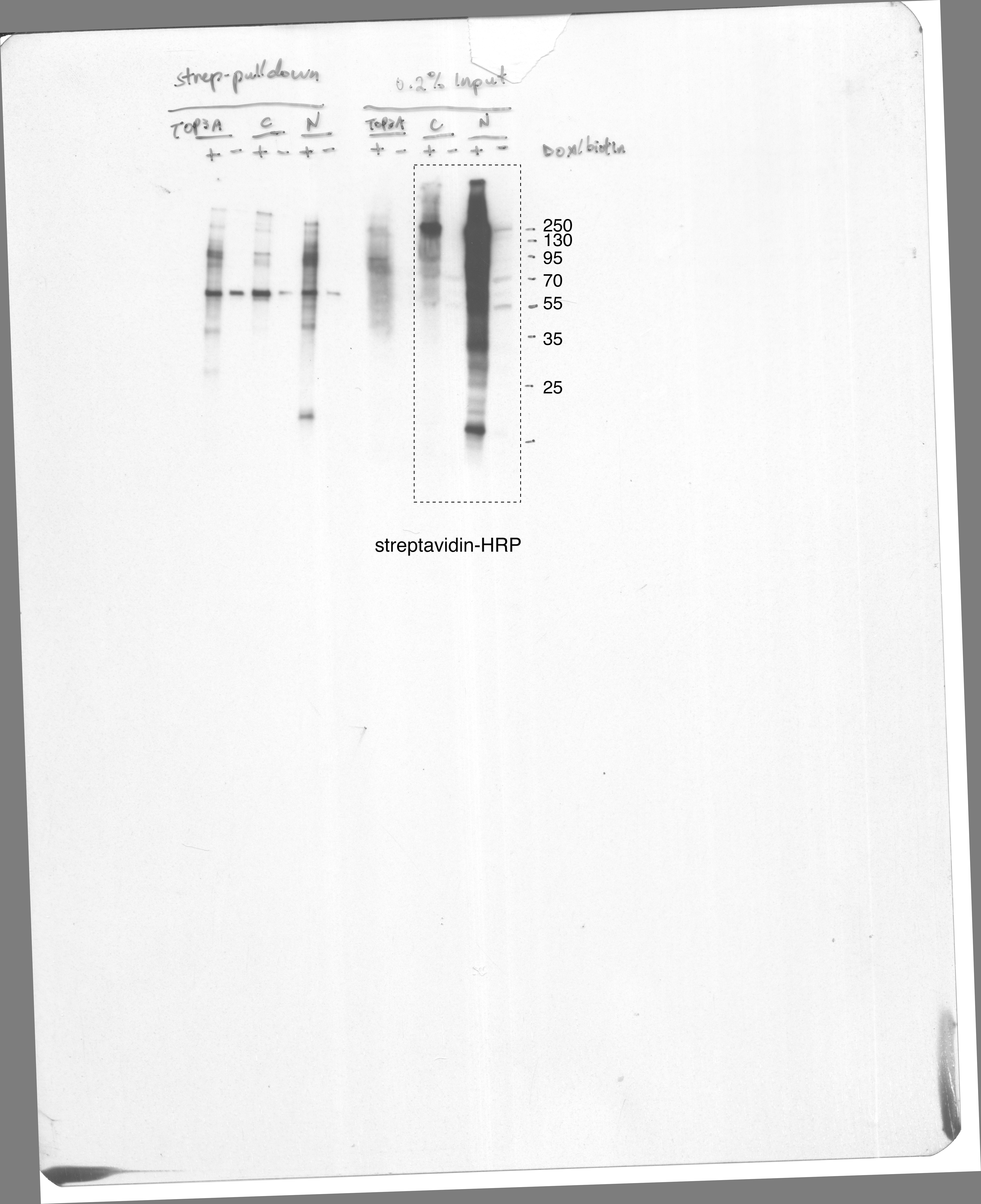

Supplement: Supplementary file 8 — EV Figure Source Data [file 44319_2025_374_MOESM8_ESM.zip › Source_Data_EV_Figures/Figure EV2/SourceDataEV2C/SourceDataForFigureEV2C.tif]

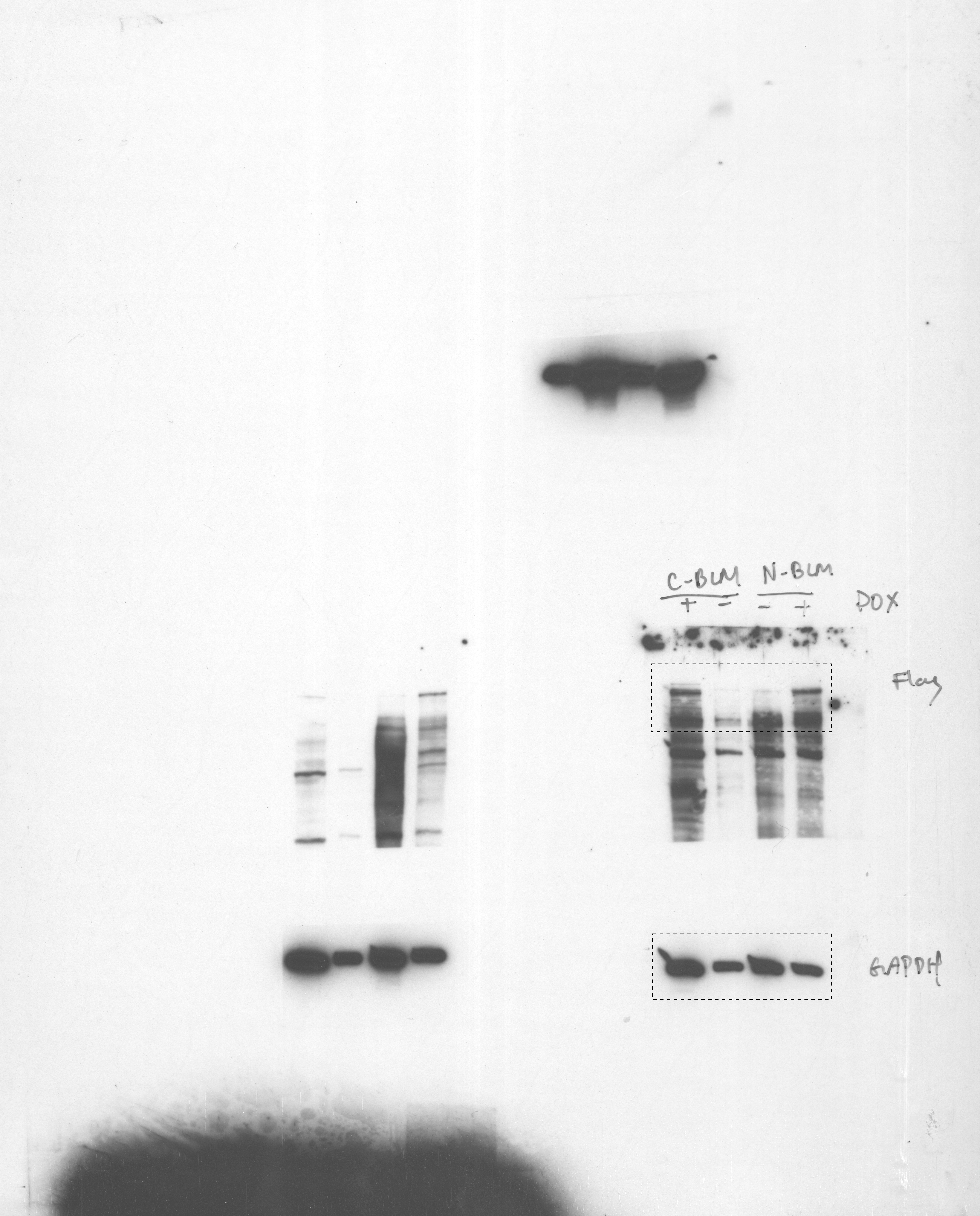

Supplement: Supplementary file 8 — EV Figure Source Data [file 44319_2025_374_MOESM8_ESM.zip › Source_Data_EV_Figures/Figure EV2/SourceDataEV2A/SourceDataForFigureEV2A.tif]

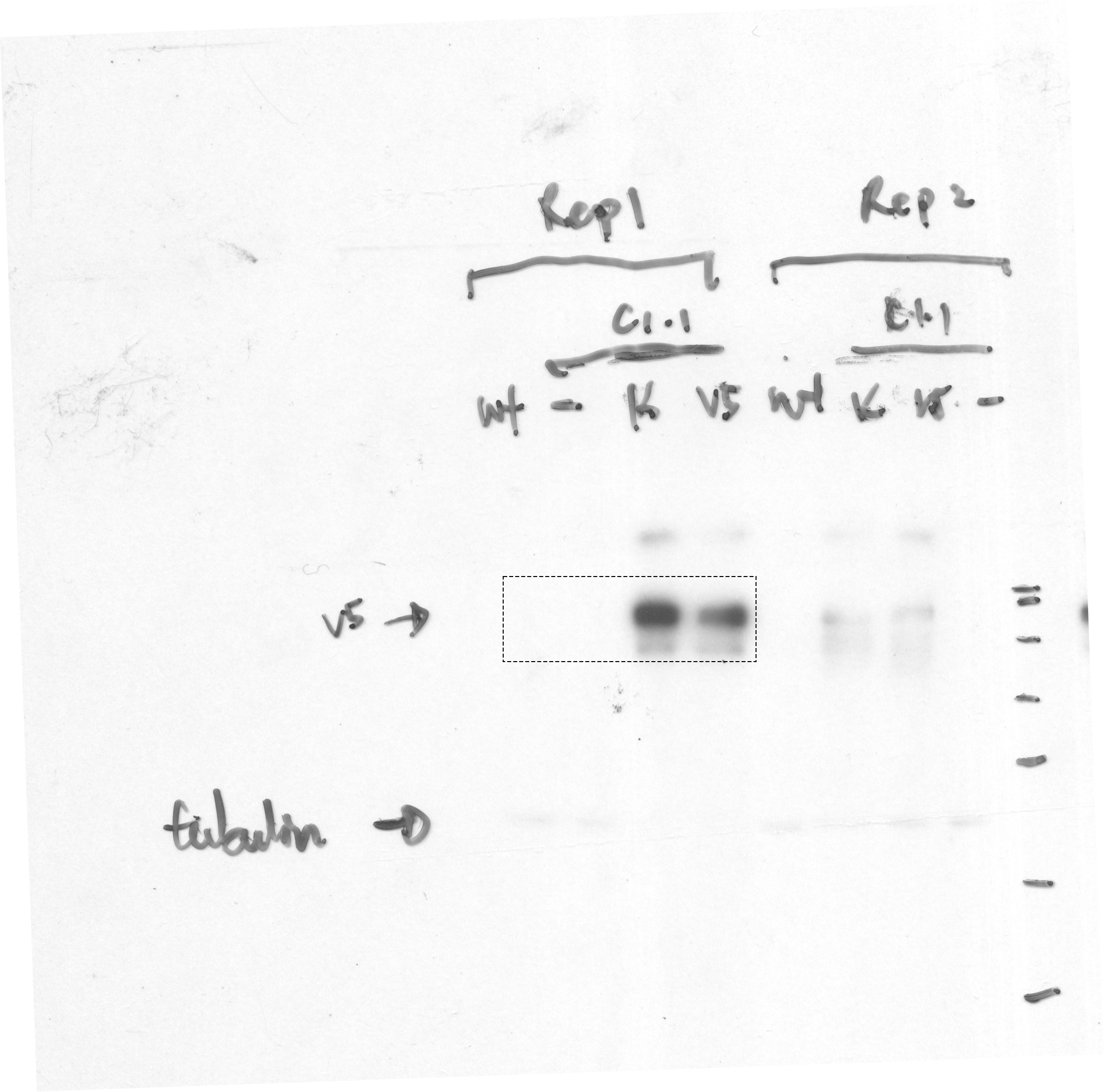

Supplement: Supplementary file 8 — EV Figure Source Data [file 44319_2025_374_MOESM8_ESM.zip › Source_Data_EV_Figures/Figure EV5/SourceDataEV5AB/SourceDataForFigureEV5AB_short_exp.tif]

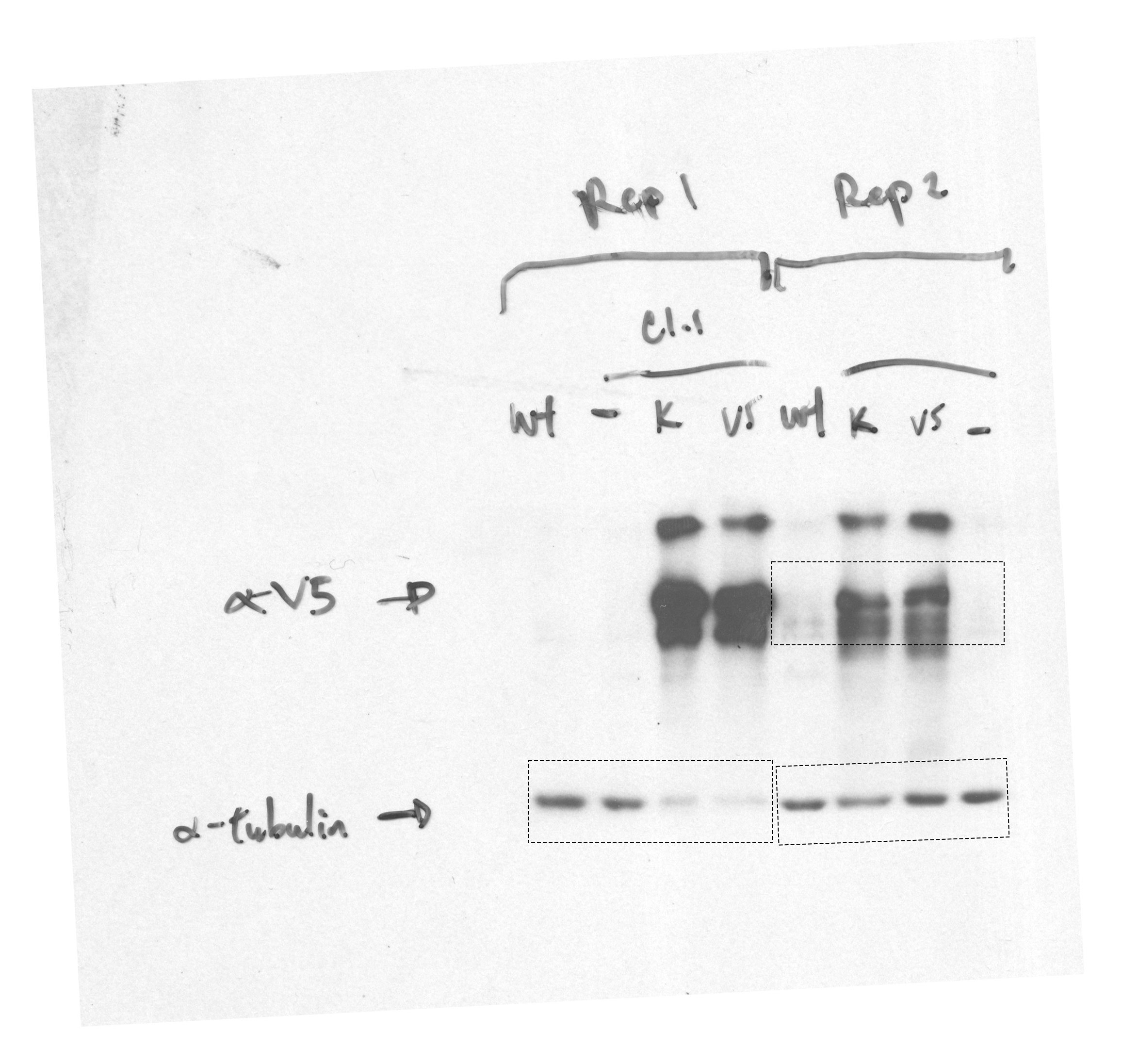

Supplement: Supplementary file 8 — EV Figure Source Data [file 44319_2025_374_MOESM8_ESM.zip › Source_Data_EV_Figures/Figure EV5/SourceDataEV5AB/SourceDataForFigureEV5AB_long_exp.tif]

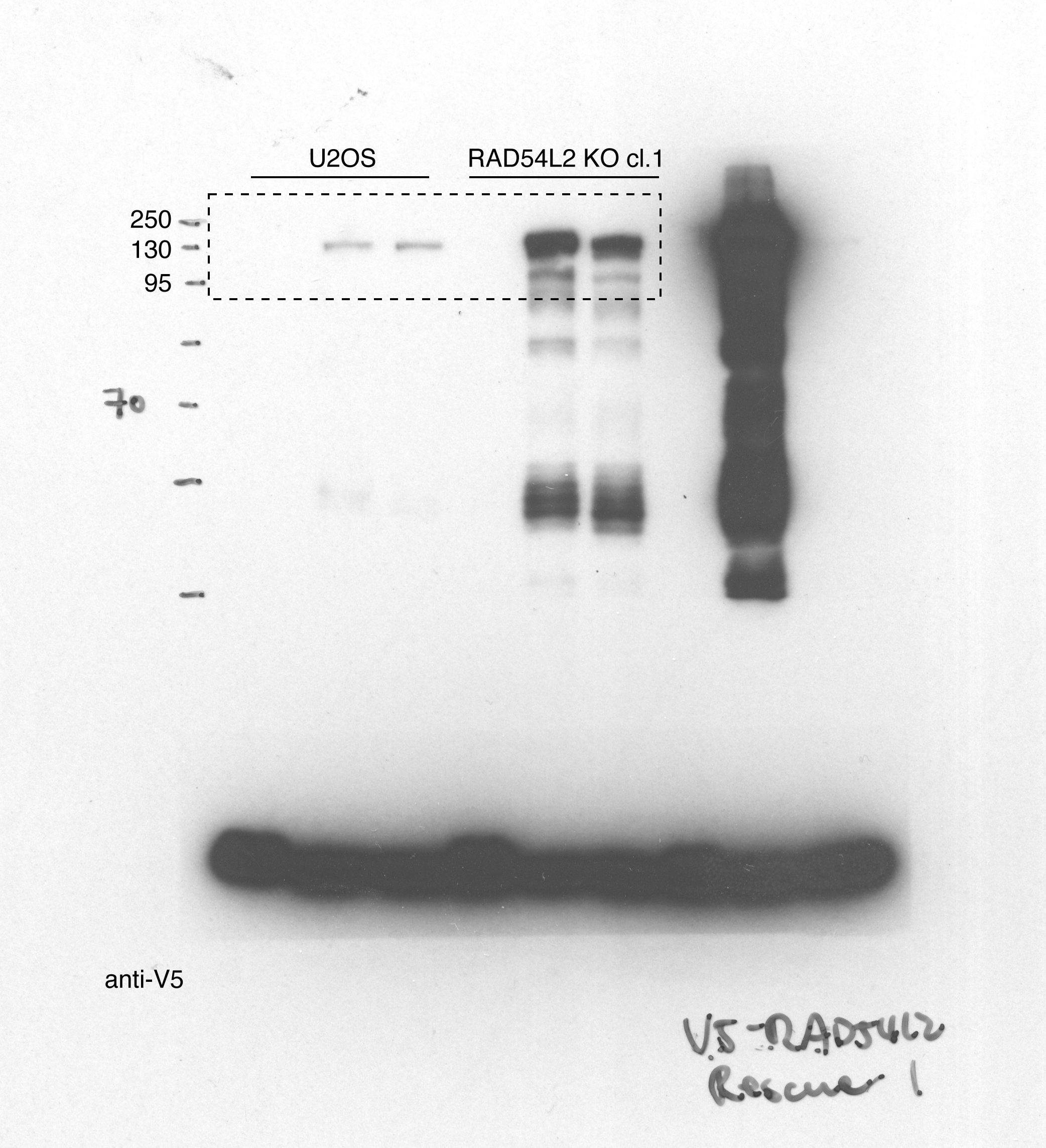

Supplement: Supplementary file 8 — EV Figure Source Data [file 44319_2025_374_MOESM8_ESM.zip › Source_Data_EV_Figures/Figure EV5/SourceDataEV5D/SourceDataForFigureEV5D.V5.tif]

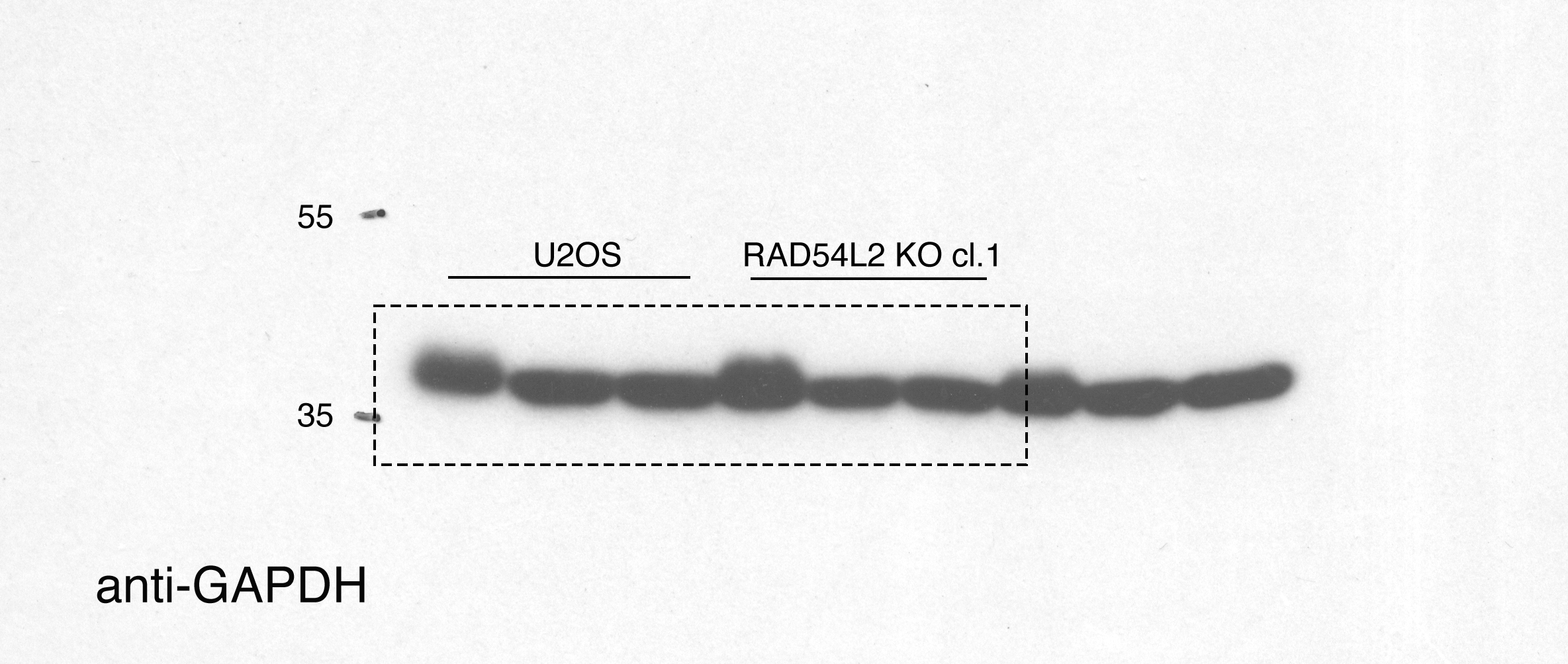

Supplement: Supplementary file 8 — EV Figure Source Data [file 44319_2025_374_MOESM8_ESM.zip › Source_Data_EV_Figures/Figure EV5/SourceDataEV5D/SourceDataForFigureEV5D.GAPDH.tif]
