# Supplementary material for: The BLM-TOP3A-RMI1-RMI2 proximity map reveals that RAD54L2 suppresses sister chromatid exchanges
Source: EMBO Rep. 2025 Jan 27;26(5):1290–314. doi: 10.1038/s44319-025-00374-z (PMC11894219; doi:10.1038/s44319-025-00374-z)
Supplement: Supplementary file 9 — Expanded View Figures [file 44319_2025_374_MOESM9_ESM.pdf]

Expanded View Figures

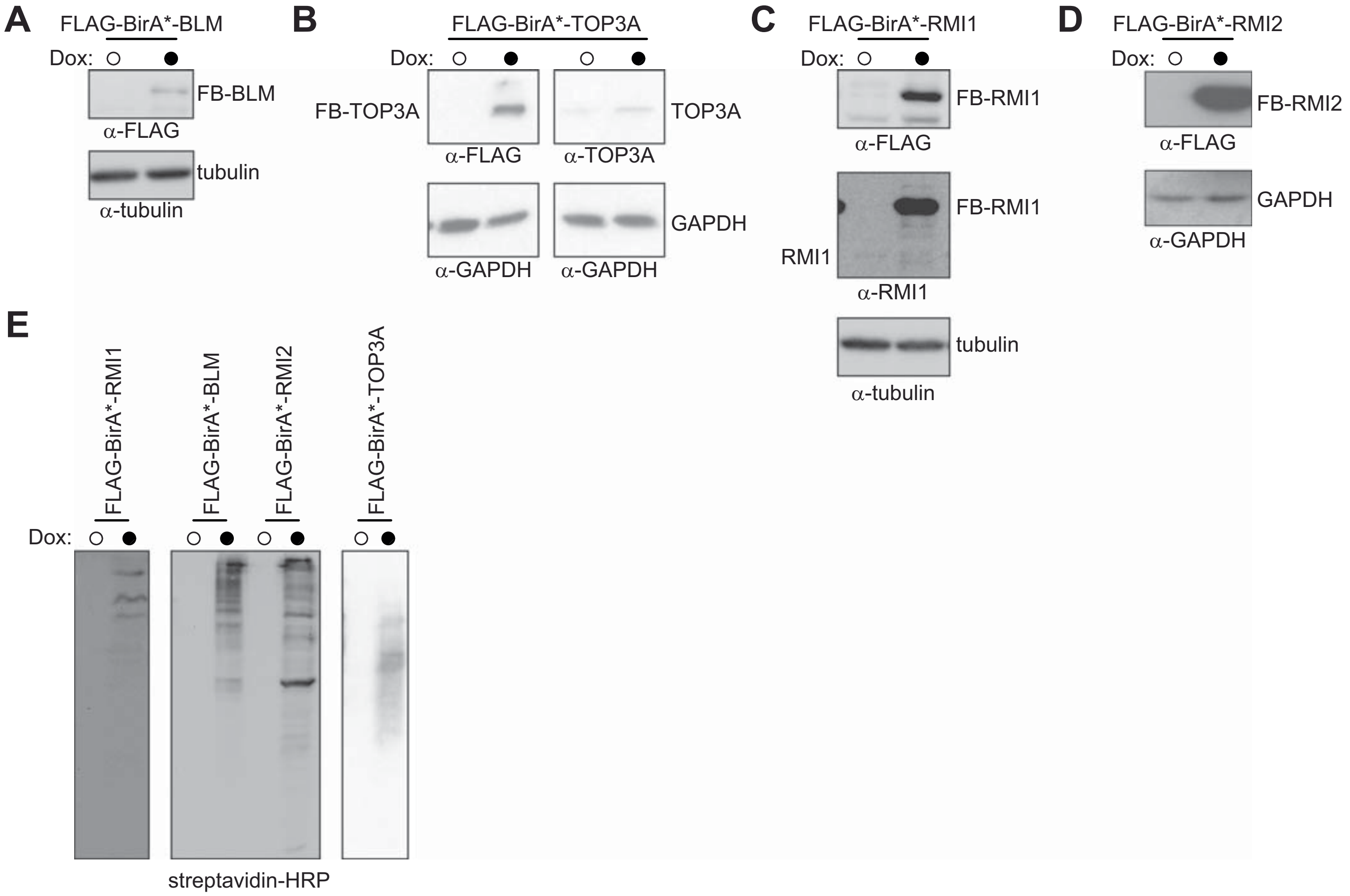

Figure EV1. Expression of active BTRR BirA* fusion proteins.

(**A**) The stable FLAG-BirA*-BLM Flp-In T-Rex HEK293 cell line was treated with doxycycline (closed circle) or vehicle (open circle) to induce expression of FLAG-BirA*-BLM (FB-BLM) and subjected to immunoblot analysis, probing with anti-FLAG or anti-tubulin antibodies, as indicated. (**B**) The stable FLAG-BirA*-TOP3A Flp-In T-Rex HEK293 cell line was treated with doxycycline (closed circle) or vehicle (open circle) to induce expression of FLAG-BirA*-TOP3A (FB-TOP3A) and subjected to immunoblot analysis, probing with anti-FLAG, anti-TOP3A, or anti-GAPDH antibodies, as indicated. (**C**) The stable FLAG-BirA*-RMI1 Flp-In T-Rex HEK293 cell line was treated with doxycycline (closed circle) or vehicle (open circle) to induce expression of FLAG-BirA*-RMI1 (FB-RMI1) and subjected to immunoblot analysis, probing with anti-FLAG, anti-RMI1, or anti-tubulin antibodies, as indicated. (**D**) The stable FLAG-BirA*-RMI2 Flp-In T-Rex HEK293 cell line was treated with doxycycline (closed circle) or vehicle (open circle) to induce expression of FLAG-BirA*-RMI2 (FB-RMI2) and subjected to immunoblot analysis, probing with anti-FLAG, or anti-GAPDH antibodies, as indicated. (**E**) Stable Flp-In T-Rex HEK293 cell lines were treated with doxycycline (closed circle) or vehicle (open circle) to induce expression of the indicated FLAG-BirA* fusion proteins. Extracts of the cells were fractionated by SDS-PAGE, and biotinylated proteins were detected with streptavidin-HRP. Source data are available online for this figure.

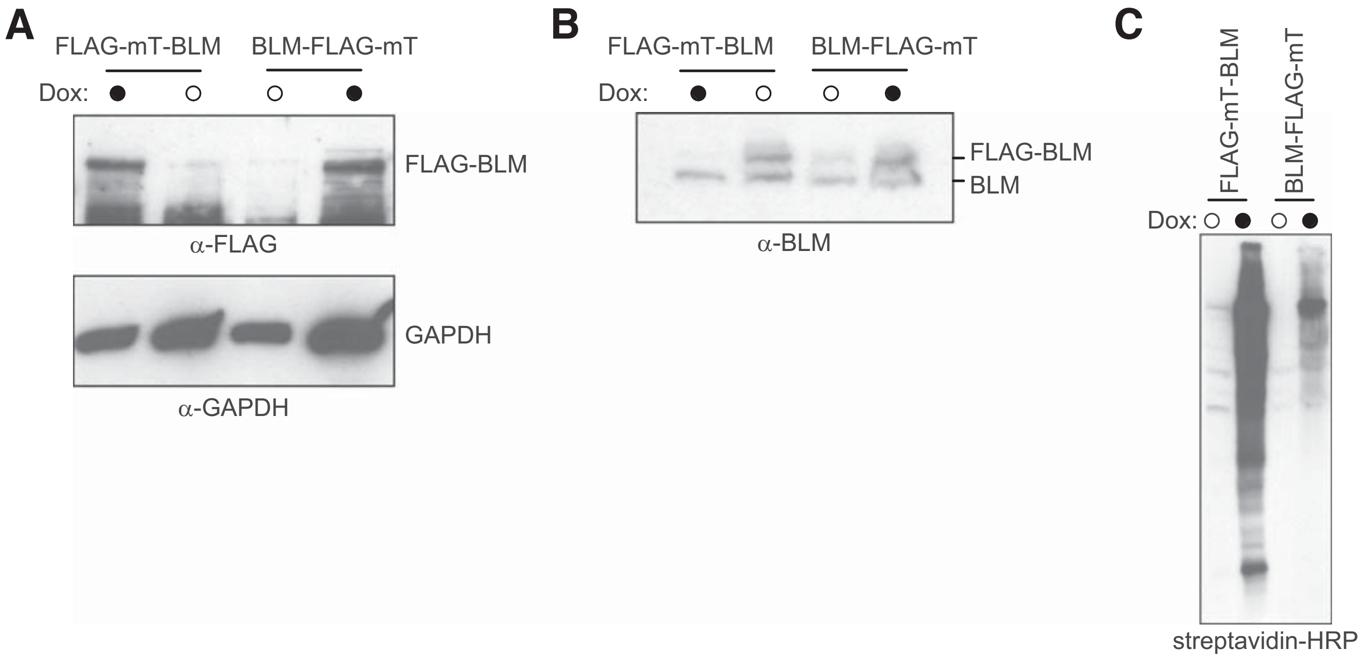

Figure EV2. Expression of active BLM miniTurbo fusions.

(**A**) The stable FLAG-miniTurbo-BLM and BLM-FLAG-miniTurbo cell lines were treated with doxycycline (closed circle) or vehicle (open circle) to induce expression of FLAG-BirA*-BLM (FB-BLM) and subjected to immunoblot analysis, probing with anti-FLAG or anti-GAPDH antibodies, as indicated. (**B**) The stable FLAG-miniTurbo-BLM and BLM-FLAG-miniTurbo cell lines were treated with doxycycline (closed circle) or vehicle (open circle) to induce expression of FLAG-BirA*-BLM (FB-BLM) and subjected to immunoblot analysis, probing with anti-BLM antibodies. (**C**) The stable FLAG-miniTurbo-BLM and BLM-FLAG-miniTurbo cell lines were treated with doxycycline (closed circle) or vehicle (open circle) to induce expression of FLAG-BirA*-BLM. Extracts of the cells were fractionated by SDS-PAGE, and biotinylated proteins were detected with streptavidin-HRP. Source data are available online for this figure.

© The Author(s)

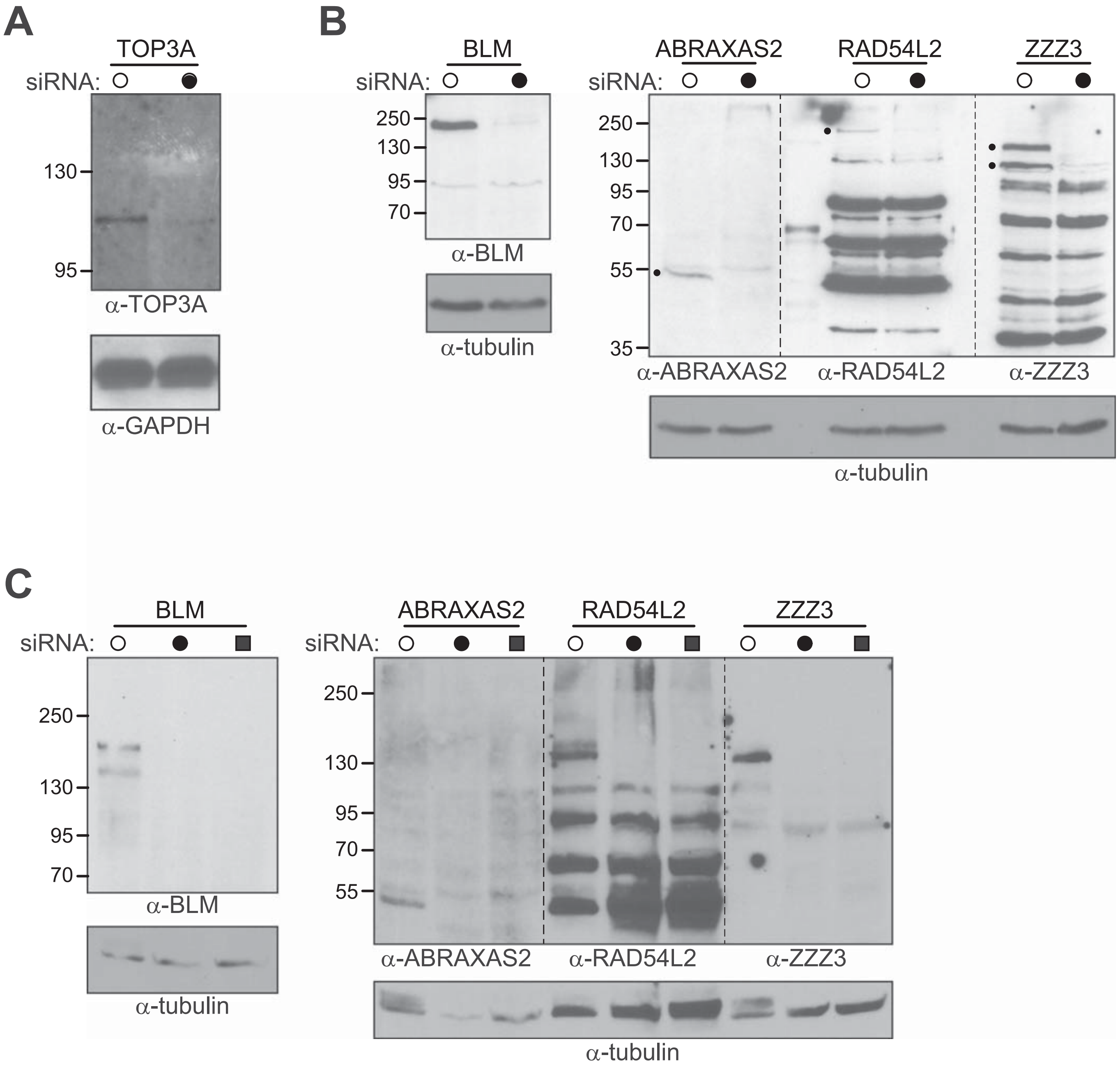

Figure EV3. Knockdown of TOP3α, BLM, ABRAXAS2, RAD54L2, and ZZZ3.

(**A**) Control (open circles) or *TOP3α* siRNA (closed circles) was transfected into U2OS cells. After 48 h, protein depletion was examined by immunoblot analysis, probing with the antibody against TOP3α or GAPDH (as a loading control). Corresponds to Fig. 3B. (**B**) Control (open circles) or the indicated siRNAs (closed circles) were transfected into U2OS cells. After 48 h, protein depletion was examined by immunoblot analysis, probing with the indicated antibodies. Anti-tubulin blots are included as loading controls. Small circles mark putative RAD54L2 and ZZZ3 polypeptides. Corresponds to Fig. 3C. (**C**) Control (open circles), the indicated siRNAs (closed circles), or the indicated siRNAs and the I-SceI expression plasmid (closed squares) were transfected into U2OS DR-GFP cells. After 48 h, protein depletion was examined by immunoblot analysis, probing with the indicated antibodies. Anti-tubulin blots are included as loading controls. Corresponds to Fig. 3D. Source data are available online for this figure.

© The Author(s)

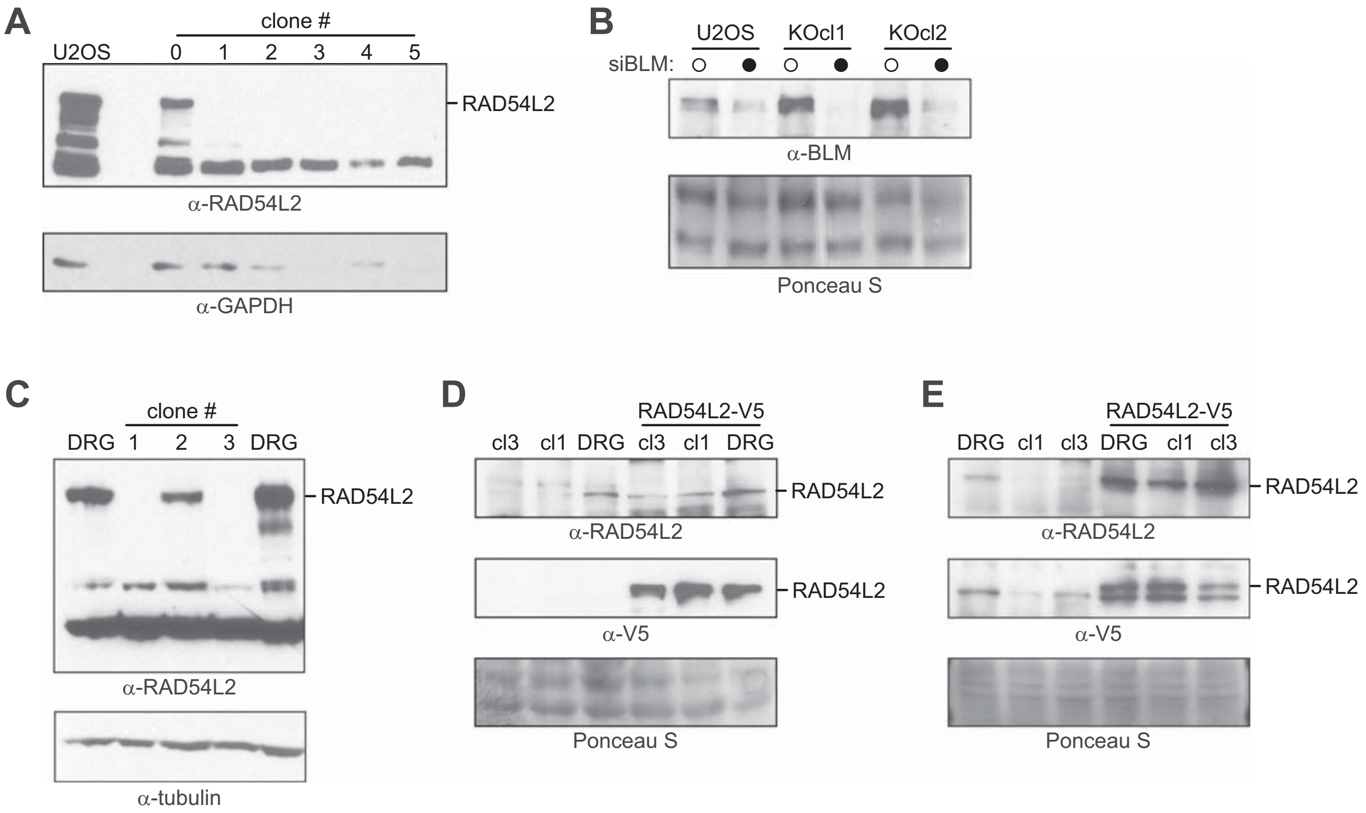

Figure EV4. CRISPR/Cas disruption of *RAD54L2*.

(**A**) U2OS cells were transfected with a plasmid expressing Cas9 and an sgRNA targeting *RAD54L2*, cloned, and examined by immunoblot analysis, probing with antibodies against RAD54L2 or against GAPDH (as a loading control). Parental cells (U2OS) were also examined. The position of RAD54L2 is indicated. Corresponds to Fig. 3E. (**B**). U2OS cells and two *RAD54L2* knockout lines (cl1, cl2) were treated with control (open circles) or *BLM* siRNAs (closed circles), and examined by immunoblot analysis, probing with antibodies against BLM. A section of the membrane stained with Ponceau S is shown as the loading control. Corresponds to Fig. 3E. (**C**) U2OS DR-GFP cells were transfected with a plasmid expressing Cas9 and an sgRNA targeting *RAD54L2*, cloned, and examined by immunoblot analysis, probing with antibodies against RAD54L2 or against tubulin (as a loading control). Parental cells (DRG) were also examined. The position of RAD54L2 is indicated. Corresponds to Fig. 3F. (**D**) U2OS DR-GFP (DRG) and two U2OS DR-GFP *RAD54L2* knockout lines (cl1, cl3) were examined by immunoblotting and probing with the indicated antibodies. A section of the membrane stained with Ponceau S is shown as the loading control. Where indicated, the cells were transfected with a plasmid carrying *RAD54L2* tagged with the V5 epitope (RAD54L2-V5). The position of RAD54L2 is indicated. Corresponds to Fig. 3F. (**E**) As in panel (**D**), for an independent experimental replicate. Corresponds to Fig. 3F. Source data are available online for this figure.

 © The Author(s)

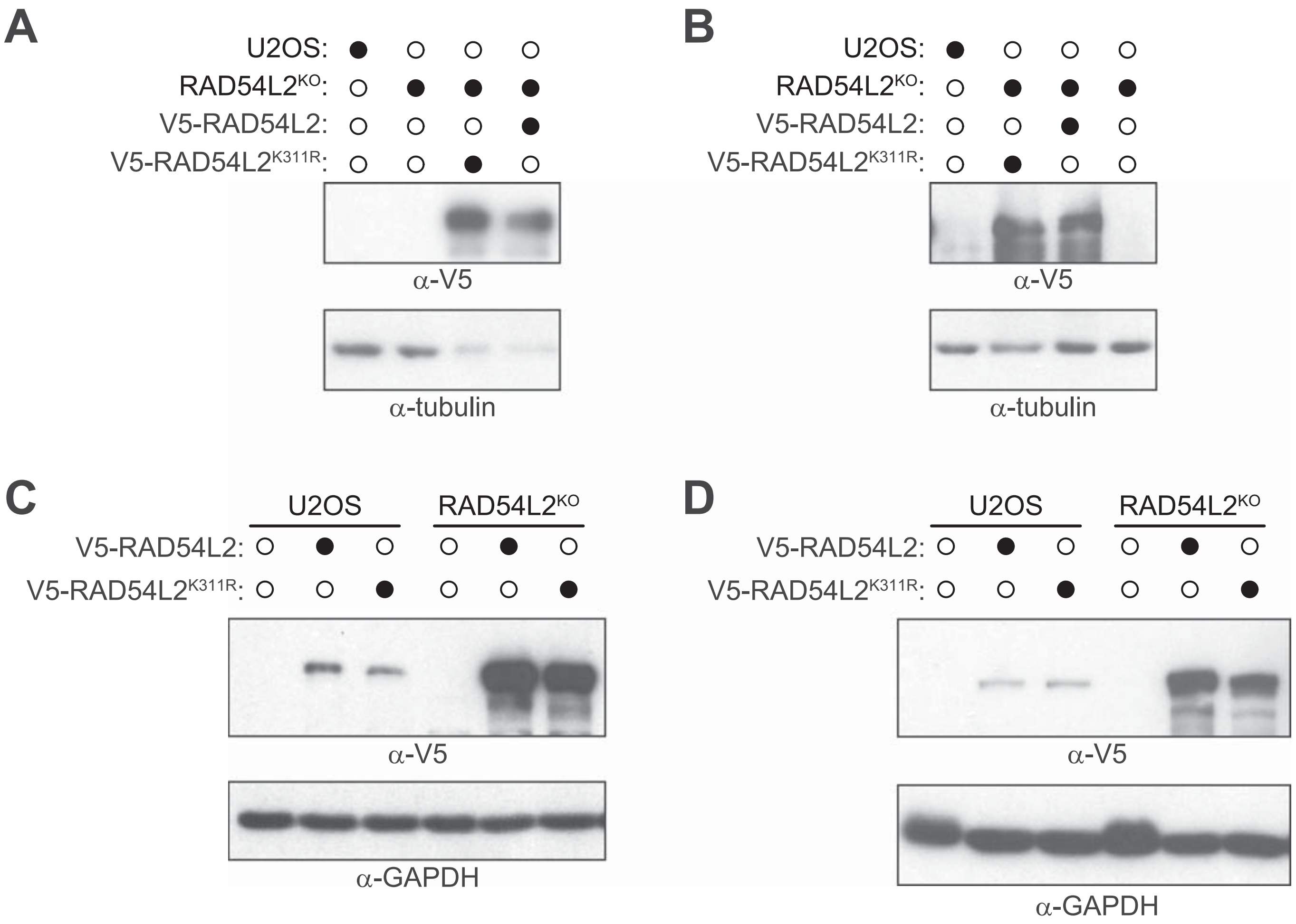

Figure EV5. Rescue of *RAD54L2* knockouts.

(**A**) *RAD54L2*-deficient U2OS cells (clone 1) were mock-transfected (open circles) or transfected with V5-tagged *RAD54L2* or *RAD54L2-K311R* (closed circles). Mock-transfected U2OS cells are also shown. Extracts of the cells were examined by immunoblot analysis, probing with antibodies against V5 or tubulin. Corresponds to Fig. 6A. (**B**) As in panel A, for the independent replicate. Corresponds to Fig. 6A. (**C**) U2OS cells or *RAD54L2*-deficient U2OS cells (clone 1) were mock-transfected (open circles) or transfected with V5-tagged *RAD54L2* or *RAD54L2-K311R* (closed circles). Extracts of the cells were examined by immunoblot analysis, probing with antibodies against V5 or GAPDH. Corresponds to Fig. 6B. (**D**) As in panel (**D**), for the independent replicate. Corresponds to Fig. 6B. Source data are available online for this figure.

© The Author(s)
